# TCGA-based identification of prognostic biomarkers and candidate traditional Chinese medicine compounds in papillary thyroid carcinoma: An observational study

**DOI:** 10.1097/MD.0000000000050562

**Published:** 2026-09-04

**Authors:** Dong Yin, Ke Meng, Qiuyang Wei, Likun Du

**Affiliations:** a Department of Endocrinology, Qingdao Traditional Chinese Medicine Hospital (Qingdao Hiser Hospital Affiliated to Qingdao University), Qingdao, Shandong, China; b Heilongjiang University of Chinese Medicine, Harbin, Heilongjiang, China; c Department of Endocrinology, First Affiliated Hospital of Heilongjiang University of Chinese Medicine, Harbin, Heilongjiang, China.

**Keywords:** immune infiltration, molecular docking, papillary thyroid carcinoma, prognostic biomarkers, TCGA, traditional Chinese medicine prediction

## Abstract

This study aimed to identify prognostic genes associated with papillary thyroid carcinoma (PTC) and explore candidate traditional Chinese medicine (TCM) compounds using integrated bioinformatics and molecular docking. In this observational study, PTC gene expression profiles and clinical data were obtained from The Cancer Genome Atlas. Differentially expressed genes were screened using differential-expression sequencing (DESeq2), followed by protein–protein interaction network analysis to identify hub genes. Their expression, diagnostic value, immune relevance, prognostic significance, protein-level validation, and single-cell distribution were assessed using gene expression profiling interactive analysis, receiver operating characteristic analysis, immune infiltration analysis, Kaplan–Meier survival analysis, the human protein atlas, and single-cell RNA-sequencing data. Candidate TCM compounds were predicted using symptom mapping (SymMap) and the TCM Systems Pharmacology Database and Analysis Platform, and molecular docking was performed to evaluate potential ligand–target interactions. Five hub genes, colony-stimulating factor 2, apolipoprotein E, fibronectin 1 (FN1), collagen type I alpha 1 chain (COL1A1), and intercellular adhesion molecule 1, were identified and found to be significantly upregulated in PTC tissues, with diagnostic value in receiver operating characteristic analysis. Immune infiltration analysis showed associations with macrophages, dendritic cells, and T helper 1 cells, whereas single-cell analysis demonstrated heterogeneous expression across immune and stromal cell populations, including fibroblasts. Higher FN1 and COL1A1 expression was associated with poorer outcomes. Immunohistochemistry supported the expression patterns, while single-cell analysis provided exploratory cell-type-level context for the cellular distribution of selected genes. Ginseng and Smilax glabra were predicted as common candidate TCMs, and docking suggested favorable binding between their active compounds and selected hub targets. Colony-stimulating factor 2, apolipoprotein E, FN1, COL1A1, and intercellular adhesion molecule 1 may be biologically relevant hub genes in PTC, while FN1 and COL1A1 may have prognostic value. Predicted TCM compounds provide preliminary computational evidence for possible compound–target interactions, requiring experimental and clinical validation.

## 1. Introduction

Papillary thyroid carcinoma (PTC) is recognized as the most prevalent malignant tumor within the endocrine system, constituting the highest incidence rate among endocrine neoplasms.^[1]^ Epidemiological studies indicate a consistent annual increase in PTC incidence by approximately 4% in recent years, with a markedly higher prevalence observed in female populations compared to males.^[2–4]^ Although ultrasound-guided fine-needle aspiration biopsy remains the gold standard for evaluating the malignancy of thyroid nodules, current literature reports that conventional diagnostic methods fail to accurately diagnose 15 to 30% of cases. Consequently, surgical intervention in these diagnostically ambiguous cases often results in significant surgical complications and heightened risks. Furthermore, the potential for excessive radioactive iodine therapy, reduced renal clearance, and drug-induced arrhythmias and bone loss further intensifies the risks associated with the treatment of thyroid cancer.^[5]^

In addition, traditional Chinese medicine (TCM) has attracted increasing attention as a source of multicomponent and multi-target therapeutic candidates. Database-based compound prediction and molecular docking can provide preliminary computational evidence for potential compound-target interactions. However, such findings should be interpreted as exploratory predictions and require further experimental validation. In this study, we integrated The Cancer Genome Atlas (TCGA)-based differential expression analysis, protein-protein interaction (PPI) network analysis, immune infiltration assessment, survival analysis, immunohistochemical validation, single-cell expression analysis, TCM compound prediction, and molecular docking to identify key genes associated with PTC prognosis and explore potential candidate compounds for future validation.

## 2. Materials and methods

### 2.1. Data source

This observational study was based on secondary bioinformatics analyses of publicly available, de-identified datasets. Using “papillary thyroid carcinoma” as the keyword, a search was conducted in TCGA database (https://portal.gdc.cancer.gov/) with the data category selected as “transcriptome profiling.” Relevant genomic data were downloaded, comprising a total of 571 samples, including 512 tumor samples and 59 normal samples. Relevant clinical information, including survival status, survival time, and pathological stage, was obtained for the available TCGA PTC cases and used for subsequent analyses.

As this study used exclusively publicly available, de-identified datasets, institutional ethical approval and informed consent were not required.

### 2.2. Differentially expressed gene (DEG) screening

The TCGA RNA-sequencing count data were normalized and analyzed using the differential-expression sequencing (DESeq2) package in R. DEGs between PTC tumor tissues and normal thyroid tissues were identified based on the criteria of |log2(fold change)| > 2 and adjusted *P* value < .05. The adjusted *P* values were calculated using the Benjamini–Hochberg method to control the false discovery rate. Heatmaps and volcano plots were generated to visualize the expression patterns and distribution of DEGs.

### 2.3. Differential gene enrichment analysis

The Database for Annotation, Visualization and Integrated Discovery tool was employed to perform Gene Ontology (GO) functional analysis on both upregulated and downregulated DEGs, covering biological processes (BP), cellular components, and molecular functions. Additionally, Kyoto Encyclopedia of Genes and Genomes (KEGG) pathway enrichment analysis was conducted. The selection criteria were set as an adjusted *P* value < .05 and a false discovery rate < 0.05. The top 10 enriched GO terms and KEGG pathways were visualized for both upregulated and downregulated genes.

### 2.4. PPI network construction and hub gene identification

The Search Tool for the Retrieval of Interacting Genes/Proteins database was used as an online tool to assess PPI networks, with the analysis criteria set to include only experimentally validated interactions and a combined score ≥ 0.4 as the threshold for significant interactions. The PPI network was visualized using Cytoscape v 3.9.1 software, and hub genes within the network were identified using the maximal clique centrality algorithm.

### 2.5. Hub gene expression and diagnostic value analysis

The Gene Expression Profiling Interactive Analysis online platform (http://gepia.cancer-pku.cn/index.html) was used to compare the expression levels of the selected hub genes between PTC tumor tissues and normal thyroid tissues. Receiver operating characteristic curve analysis was performed to evaluate the diagnostic performance of each hub gene in distinguishing PTC tumor samples from normal samples. The area under the curve was calculated to assess diagnostic accuracy.

### 2.6. Hub gene immune infiltration analysis

Immune cell infiltration was assessed using single-sample gene set enrichment analysis (ssGSEA) implemented in the gene set variation analysis package (version 1.46.0) in R version 4.2.1. TCGA–thyroid carcinoma RNA-sequencing data processed using the Spliced Transcripts Alignment to a Reference workflow were obtained in transcripts per million format. Normal samples were excluded, and expression values were transformed as log2(transcripts per million + 1). Marker gene sets representing 24 immune cell types were derived from Bindea et al^[6]^ and used to calculate ssGSEA enrichment scores for individual tumor samples. Spearman correlation analysis was performed to evaluate the associations between hub gene expression levels and the corresponding immune cell infiltration scores. The correlation results were visualized as lollipop plots using the ggplot2 package (version 3.4.4).

### 2.7. Hub gene prognostic analysis

The prognostic value of the identified hub genes in PTC was evaluated using clinical survival data and the Kaplan–Meier (KM)-plotter database. Patients were divided into high- and low-expression groups according to the median expression level of each hub gene. KM survival curves were generated to assess the association between hub gene expression and overall survival. Survival differences between groups were evaluated using the log-rank test, and *P* < .05 was considered statistically significant.

### 2.8. Immunohistochemical analysis

The Human Protein Atlas (HPA) analysis platform, which visualizes all human proteins in cells, tissues, and organs using various omics techniques, was employed for immunohistochemical analysis. Following prognostic analysis, genes associated with the survival of PTC patients were confirmed, and their immunohistochemical expression in PTC tissues was analyzed using the HPA database.

### 2.9. Single-cell expression analysis of hub genes

Single-cell expression data were obtained from the GSE148673 dataset, which includes 46,501 single cells from 21 tumors, covering triple-negative breast cancer, pancreatic ductal adenocarcinoma, undifferentiated thyroid carcinoma, invasive ductal carcinoma, and glioblastoma. This dataset distinguishes cancer cells from normal cell types with an accuracy of 98%. Corresponding single-cell data in.h5 format and annotation results were downloaded from tumor immune single-cell hub. The data were processed and analyzed using the R packages Model-based AnalysEs of Single-cell Transcriptome and RegulOme and Seurat, with cell clustering performed using the t-distributed stochastic neighbor embedding method.

### 2.10. Prediction of TCMs and components

Hub genes were mapped to corresponding TCMs using the symptom mapping (SymMap) database (http://www.symmap.org/). The TCM Systems Pharmacology Database and Analysis Platform (https://www.tcmsp-e.com/#/home) was utilized to screen for effective active components, setting criteria of oral bioavailability ≥ 30% and drug-likeness ≥ 0.18. TCMs associated with each hub gene were compiled, and those related to all 5 hub genes were identified. The active components of these TCMs were then selected as core active components.

### 2.11. Molecular docking validation

Molecular docking was performed to explore potential interactions between hub gene-encoded proteins and the predicted active compounds. The 2D structures of the active compounds were retrieved from the PubChem database (https://pubchem.ncbi.nlm.nih.gov/) and converted into 3D structures using ChemBio 3D 14.0.0, followed by energy minimization. The 3D structures of target proteins were downloaded from the protein data bank database (https://www.rcsb.org/). Python-based Molecular Visualization was used to remove small-molecule ligands and water molecules from the protein structures, and the final docking results were visualized using Python-based Molecular Visualization. Binding energies were calculated to estimate the predicted ligand-target affinity. Lower binding energies were considered to indicate more favorable predicted interactions; however, molecular docking was interpreted only as preliminary computational evidence and could not confirm biological activity or therapeutic efficacy.

## 3. Results

### 3.1. Identification of DEGs in PTC

**Figure 1.**
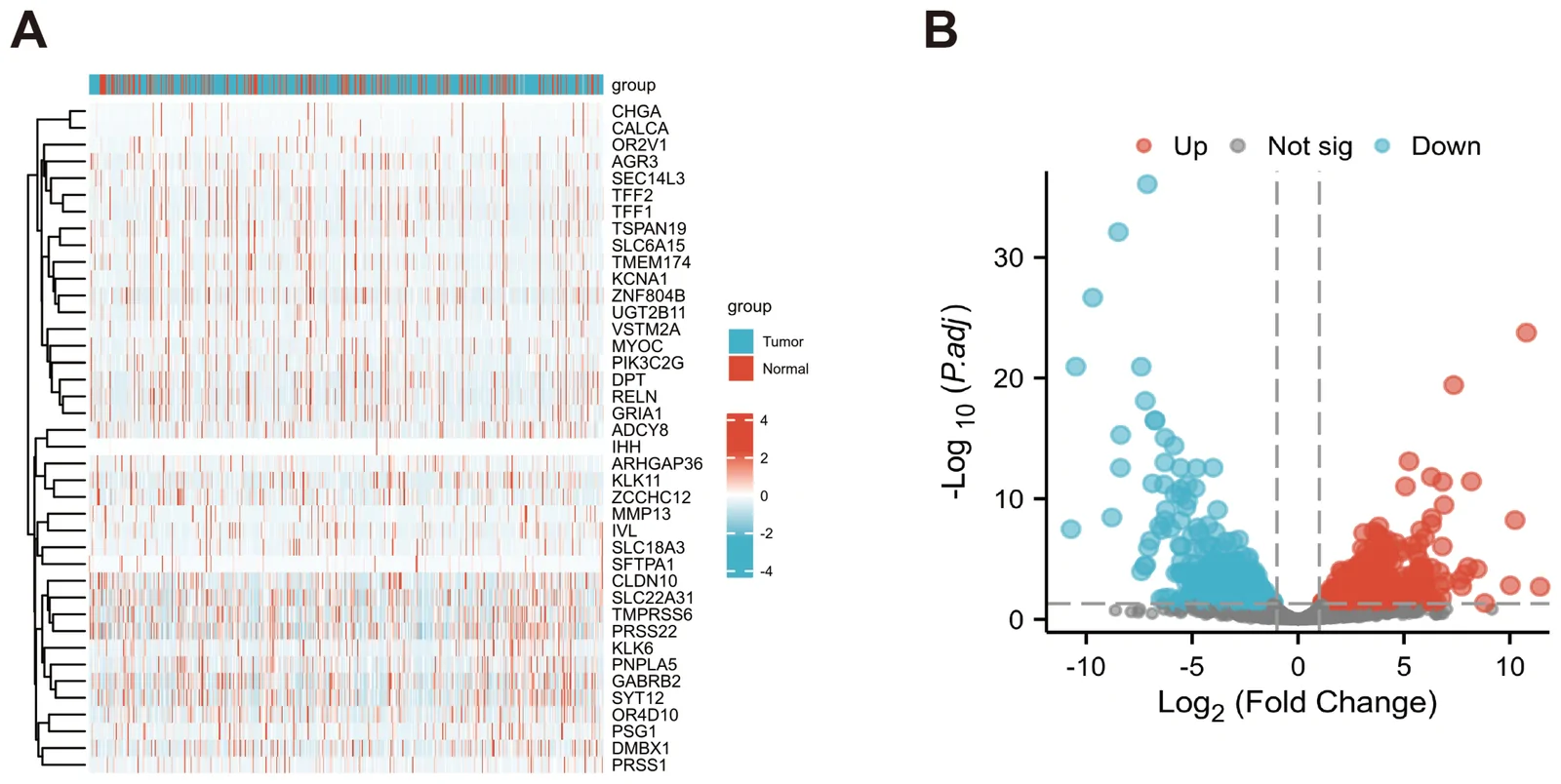
DEGs. (A) Heatmap of DEGs and (B) volcano plot of DEGs. Red indicates upregulated genes, and blue indicates downregulated genes. DEG = differentially expressed gene.

Differential expression analysis identified 1963 genes with significant expression changes, including 1327 upregulated and 636 downregulated genes. A heatmap was generated to visually represent the top 20 most significantly upregulated and downregulated genes, as illustrated in Figure 1.

### 3.2. Enrichment analysis of DEGs in PTC

**Figure 2.**
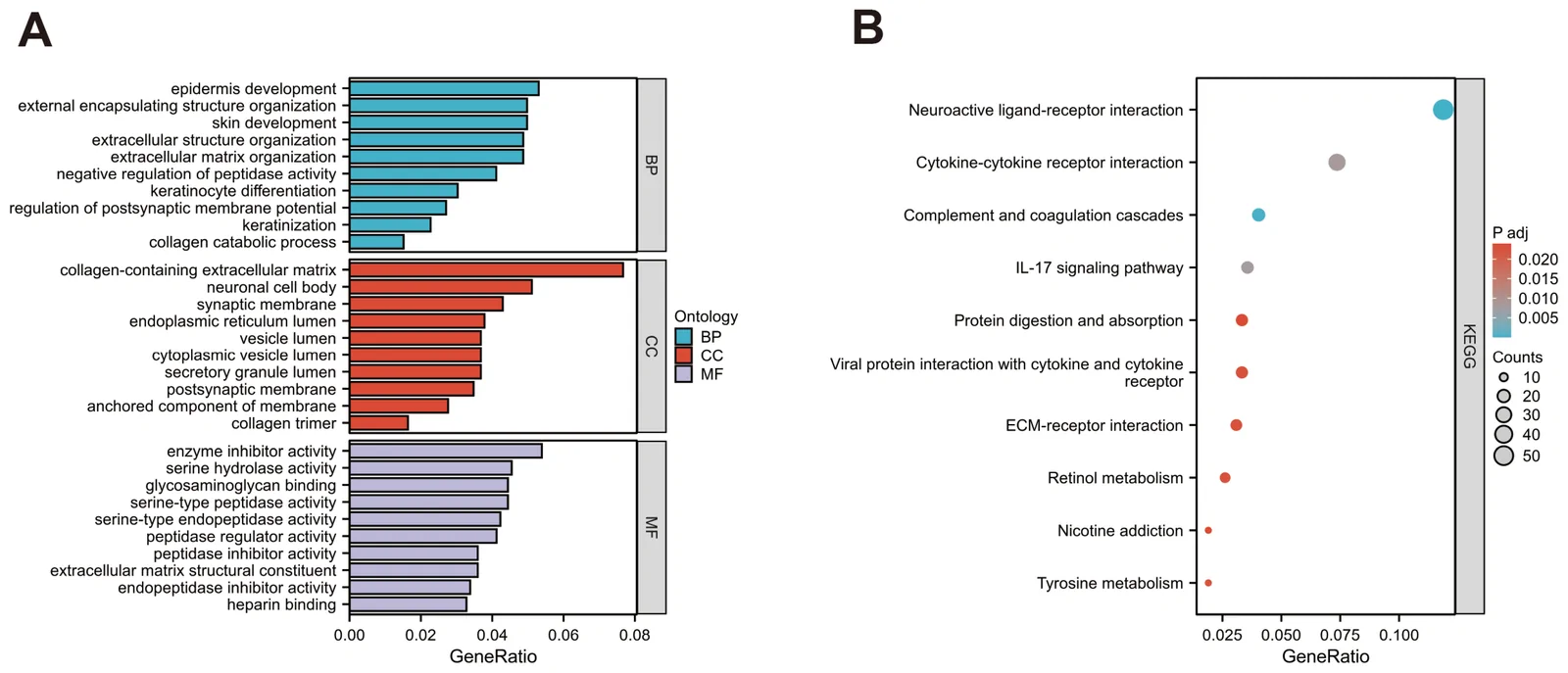
Enrichment analysis of DEGs. (A) GO functional analysis results and (B) KEGG pathway enrichment analysis results. BP = biological process, CC = cellular component, DEG = differentially expressed gene, ECM = extracellular matrix, GO = gene ontology, IL = interleukin, KEGG = Kyoto Encyclopedia of Genes and Genomes, MF = molecular function.

GO analysis of DEGs revealed that these genes are primarily involved in BP such as extracellular structure organization, extracellular matrix (ECM) organization, and collagen catabolic processes. They are mainly localized in cellular components such as the collagen-containing ECM and collagen trimer. The molecular functions associated with these genes include hydrolase activity, ECM structural constituent, and endopeptidase inhibitor activity, as shown in Figure 2A. KEGG pathway analysis indicated that the DEGs are predominantly involved in the regulation of pathways such as neuroactive ligand-receptor interaction, cytokine-cytokine receptor interaction, and complement and coagulation cascades, as depicted in Figure 2B.

### 3.3. Hub gene selection

**Figure 3.**
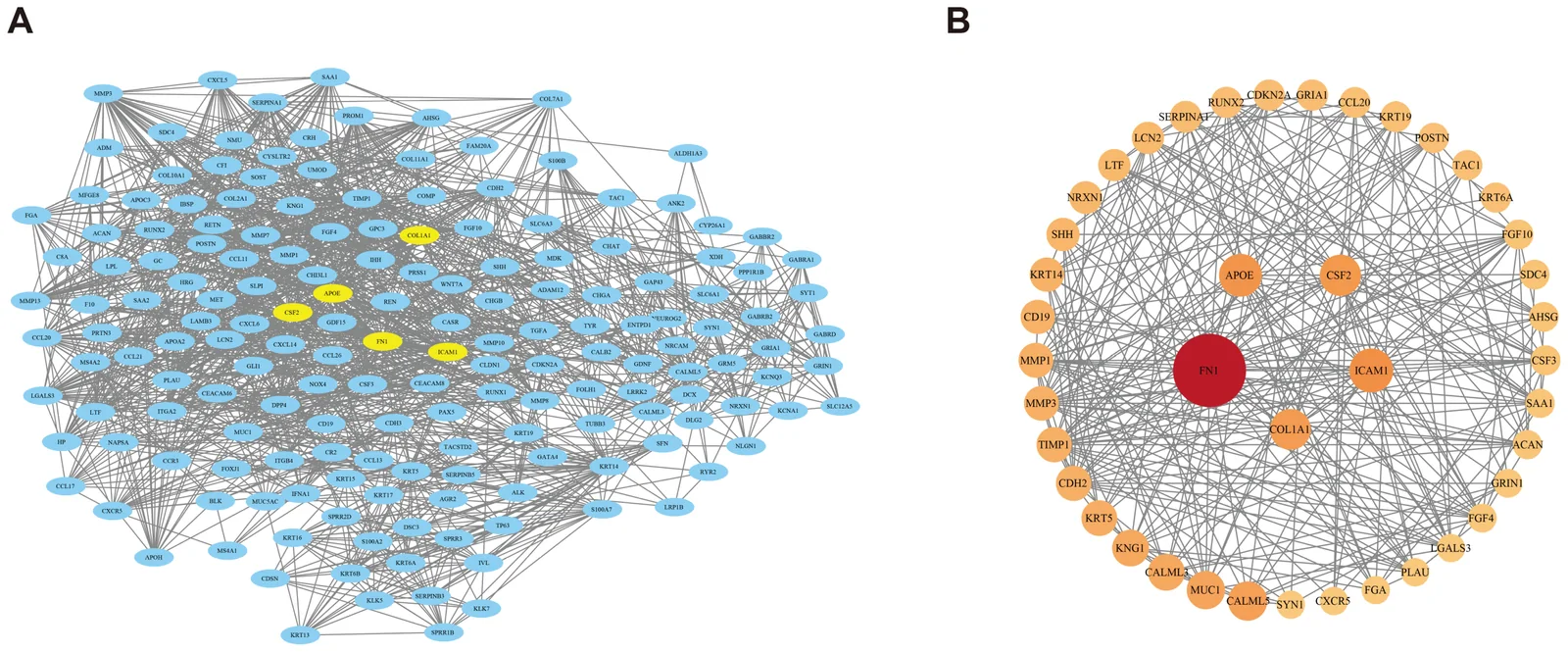
PPI network and hub genes. (A) PPI network and (B) identification of the top 5 hub genes using the MCC algorithm. APOE = apolipoprotein E, COL1A1 = collagen type I alpha 1 chain, CSF2 = colony-stimulating factor 2, FN1 = fibronectin 1, ICAM = intercellular adhesion molecule, MCC = maximal clique centrality, PPI = protein-protein interaction.

The interaction network of DEGs revealed a total of 1030 nodes and 1148 edges. After importing the PPI network into Cytoscape software, the maximal clique centrality algorithm was used to identify hub genes within the network, as shown in Figure 3A. The top 5 hub genes were selected: colony-stimulating factor 2 (CSF2), apolipoprotein E (APOE), fibronectin 1 (FN1), collagen type I alpha 1 chain (COL1A1), and intercellular adhesion molecule (ICAM)1, as illustrated in Figure 3B.

### 3.4. Validation of hub gene expression

**Figure 4.**
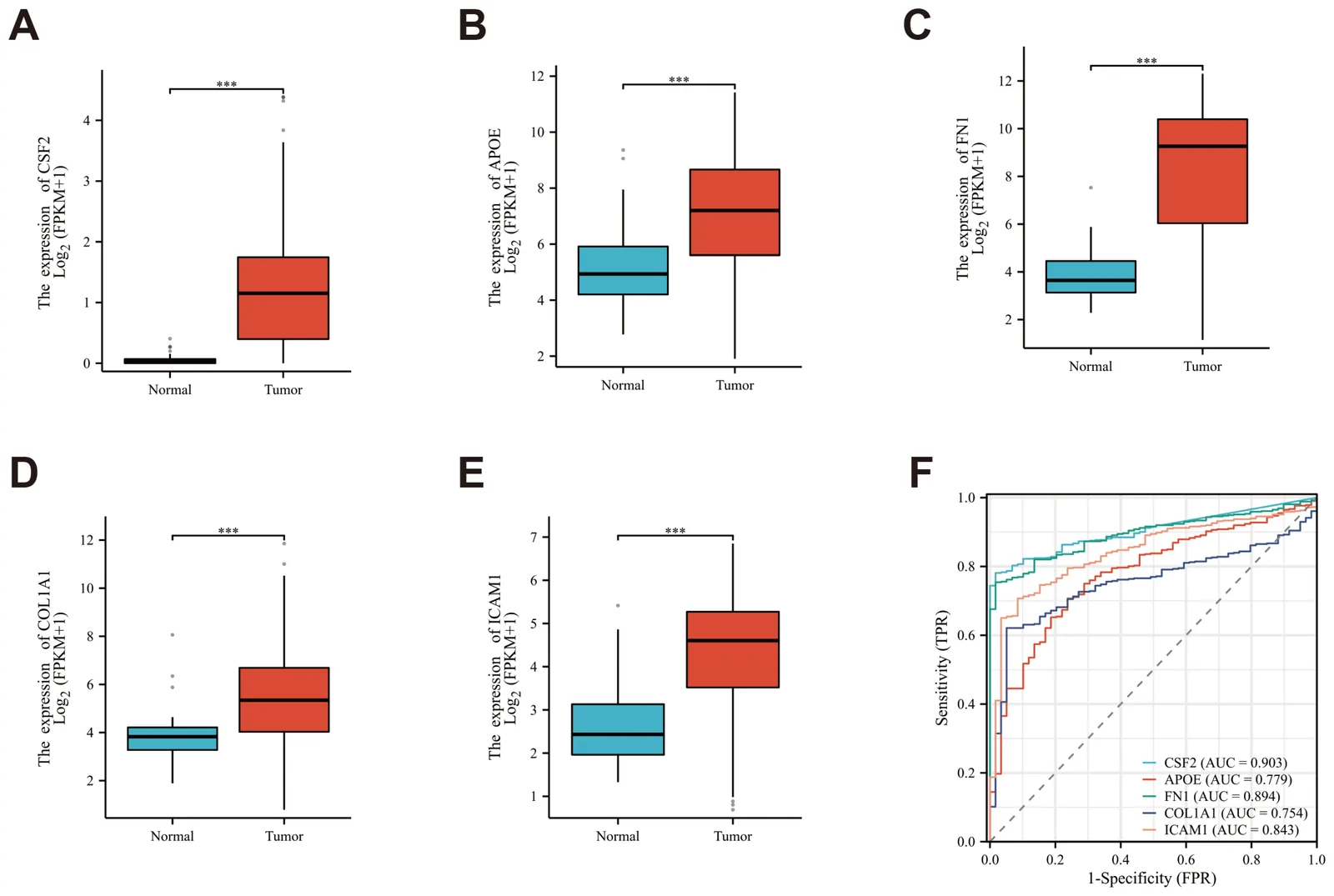
Hub gene expression and ROC analysis. (A–E) Expression levels of CSF2, APOE, FN1, COL1A1, and ICAM1 in tumor and normal tissues. Red indicates tumor tissue, and blue indicates normal tissue. *P < .05. (F) Diagnostic value of hub genes as assessed by ROC curves. APOE = apolipoprotein E, AUC = area under the curve, COL1A1 = collagen type I alpha 1 chain, CSF2 = colony-stimulating factor 2, FN1 = fibronectin 1, ICAM = intercellular adhesion molecule, ROC = receiver operating characteristic.

The expression levels of the 5 hub genes (CSF2, APOE, FN1, COL1A1, and ICAM1) were significantly elevated in tumor tissues compared to normal tissues, as shown in Figure 4. The area under the curve values of the receiver operating characteristic curves were > 0.7 for all 5 hub genes, suggesting that these genes had potential diagnostic value in distinguishing PTC tumor tissues from normal thyroid tissues.

### 3.5. Immune infiltration analysis

**Figure 5.**
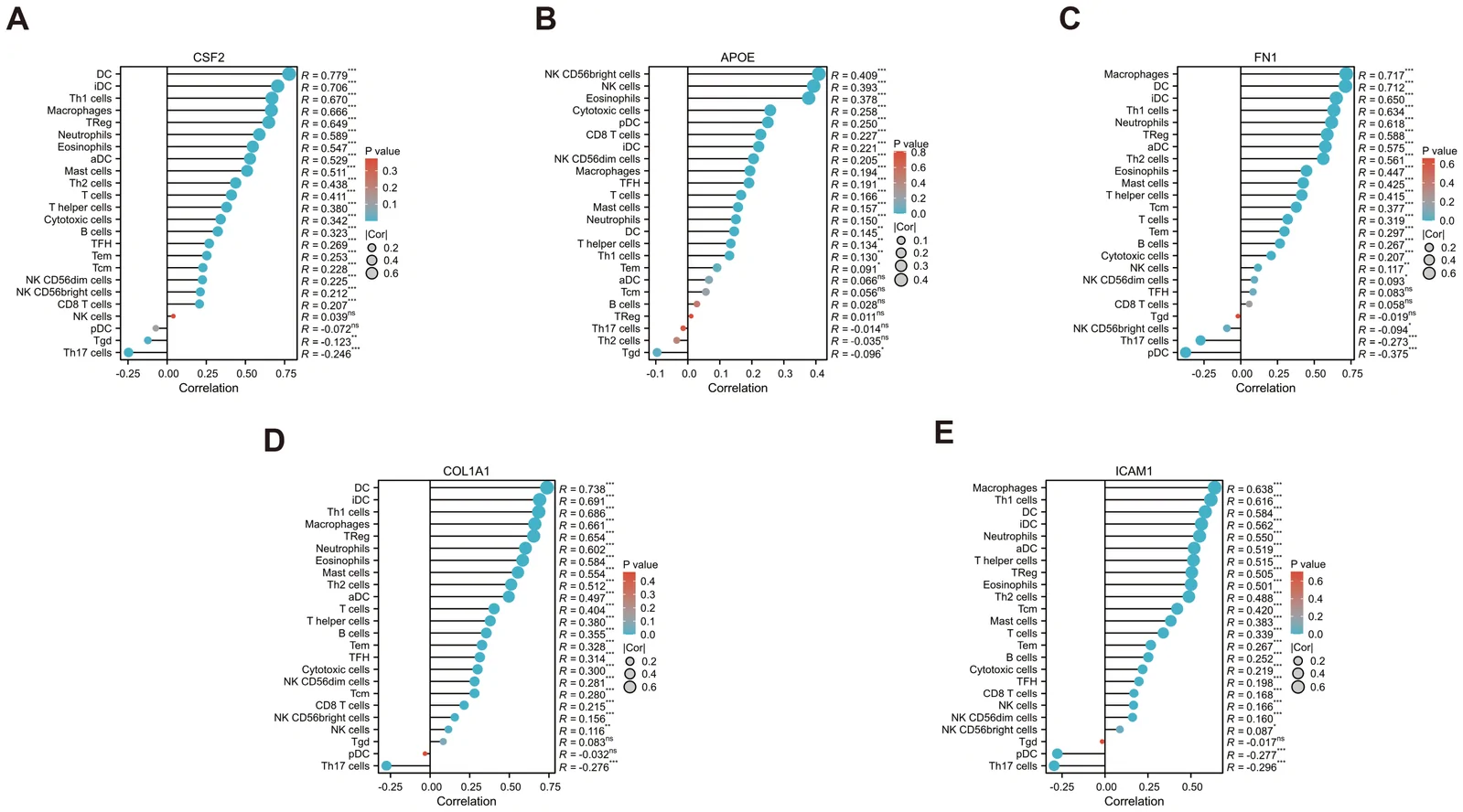
Immune infiltration analysis of hub genes. APOE = apolipoprotein E, COL1A1 = collagen type I alpha 1 chain, CSF2 = colony-stimulating factor 2, FN1 = fibronectin 1, ICAM = intercellular adhesion molecule.

ssGSEA-based immune infiltration analysis revealed associations between hub gene expression and the infiltration scores of several immune cell populations. CSF2 expression was positively correlated with the ssGSEA-derived infiltration scores of dendritic cells and T helper 1 (Th1) cells, whereas APOE expression was associated with natural killer cells and eosinophils. FN1 expression was associated with macrophages and dendritic cells, and COL1A1 expression showed associations with dendritic cells and Th1 cells. ICAM1 expression was associated with macrophages and Th1 cells. These correlations are illustrated in Figure 5.

### 3.6. Survival curve analysis

**Figure 6.**
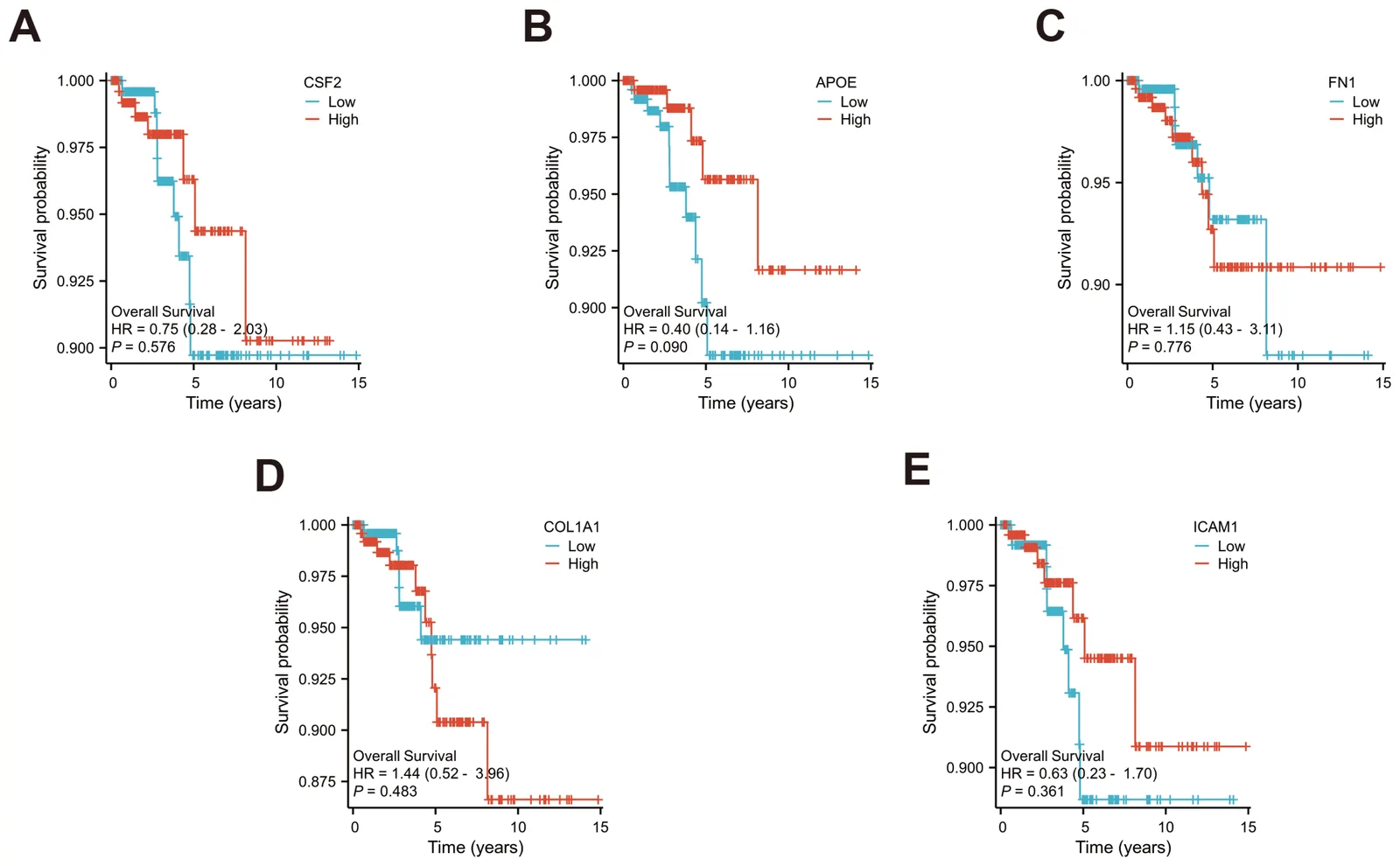
KM survival analysis of hub genes in PTC. APOE = apolipoprotein E, COL1A1 = collagen type I alpha 1 chain, CSF2 = colony-stimulating factor 2, FN1 = fibronectin 1, HR = hazard ratio, ICAM = intercellular adhesion molecule, KM = Kaplan–Meier, PTC = papillary thyroid carcinoma.

KM survival analysis showed that the expression levels of the hub genes were associated with overall survival in patients with PTC. Among them, higher expression levels of FN1 and COL1A1 were more closely associated with poorer patient outcomes, suggesting their potential prognostic relevance in PTC, as shown in Figure 6.

### 3.7. HPA immunohistochemical analysis

**Figure 7.**
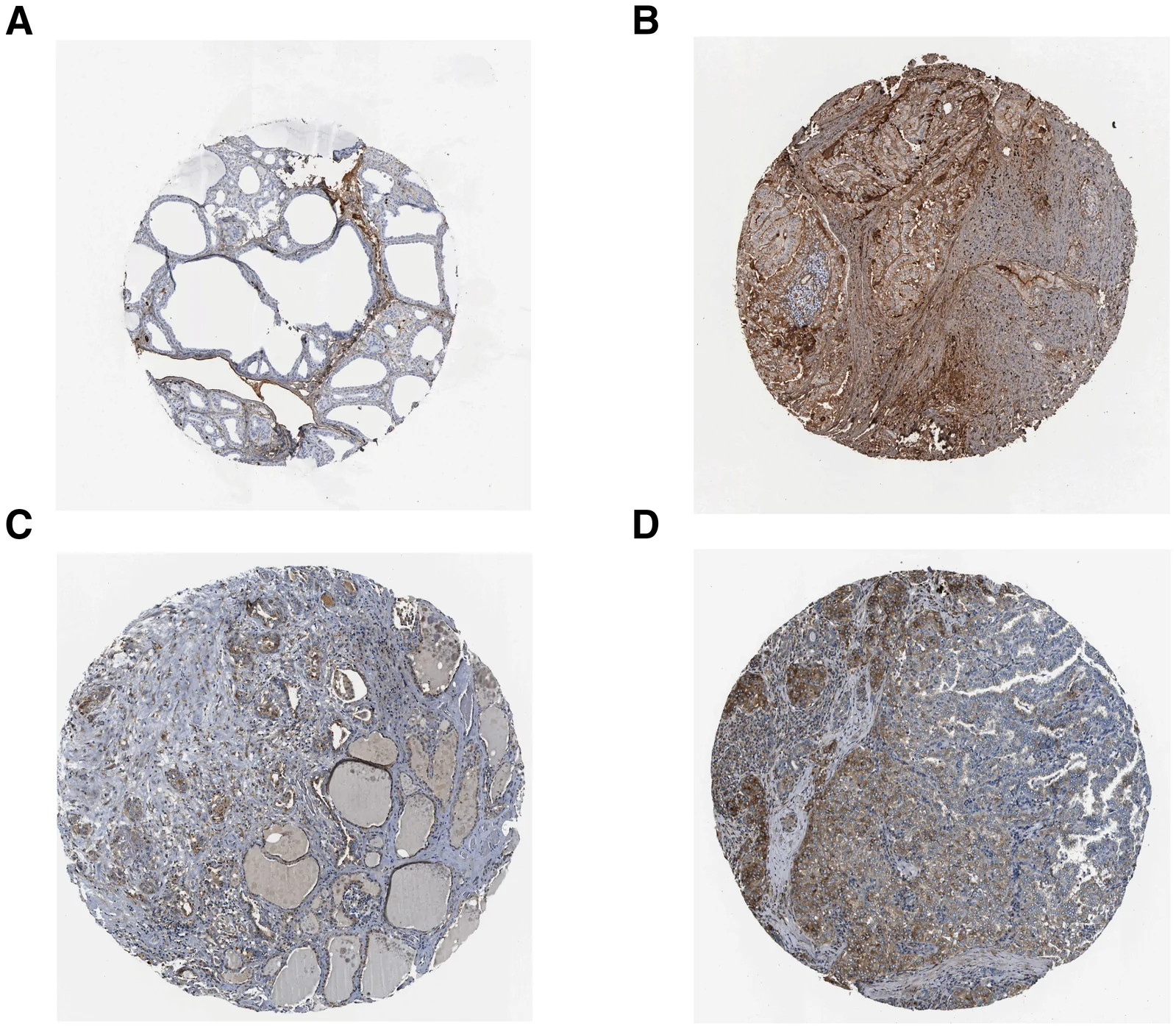
HPA immunohistochemistry of FN1 and COL1A1. (A) FN1 expression in normal thyroid tissue; (B) FN1 expression in PTC tissue; (C) COL1A1 expression in normal thyroid tissue; (D) COL1A1 expression in PTC tissue. COL1A1 = collagen type I alpha 1 chain, FN1 = fibronectin 1, HPA = Human Protein Atlas, PTC = papillary thyroid carcinoma.

A search for the relevant genes in the HPA revealed that FN1 and COL1A1 are primarily localized in the cytoplasm and cell membrane. Immunohistochemical results from the database provided protein-level support for the expression of FN1 and COL1A1 in PTC tissues, as shown in Figure 7.

### 3.8. Single-cell expression analysis of hub genes

**Figure 8.**
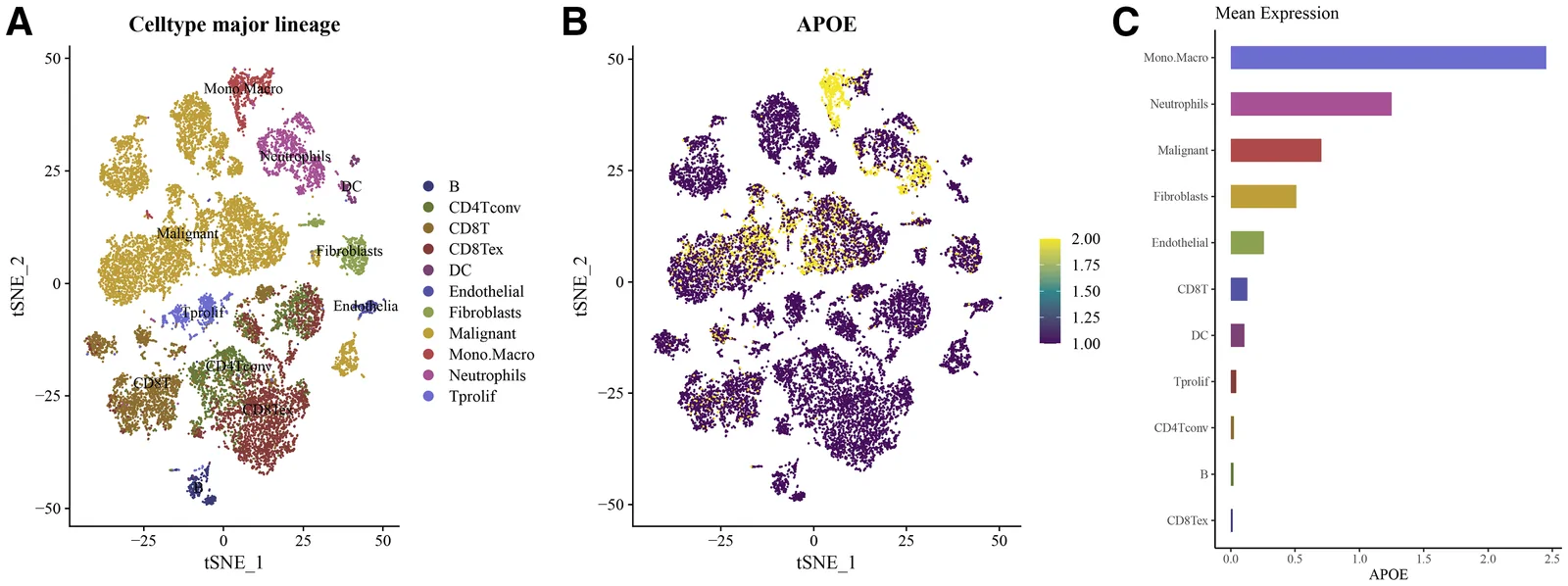
APOE expression levels in single-cell data. (A) t-SNE plot of single-cell clustering, with different colors representing different cell types; (B) t-SNE plot showing the distribution of APOE expression across different cells; (C) bar chart showing APOE expression abundance across different cell types. APOE = apolipoprotein E, t-SNE = t-distributed stochastic neighbor embedding.

**Figure 9.**
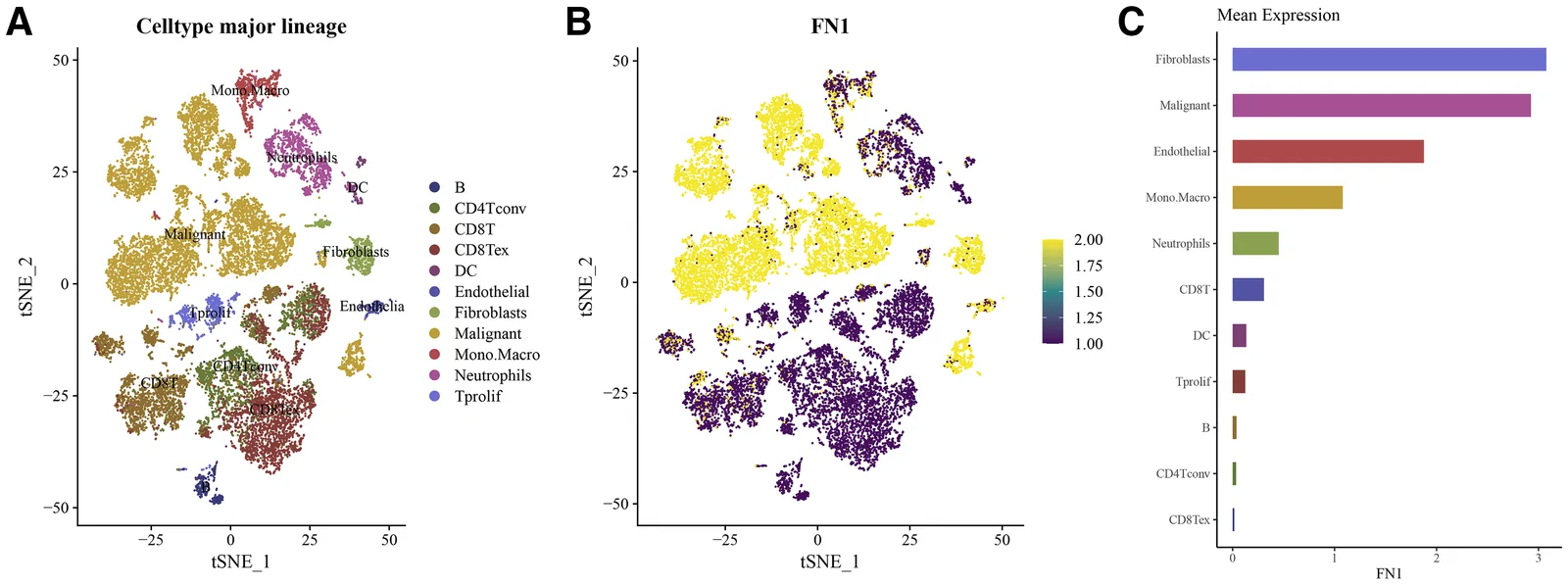
FN1 expression levels in single-cell data. (A) t-SNE plot of single-cell clustering, with different colors representing different cell types; (B) t-SNE plot showing the distribution of FN1 expression across different cells; (C) bar chart showing FN1 expression abundance across different cell types. FN1 = fibronectin 1, t-SNE = t-distributed stochastic neighbor embedding.

The single-cell expression analysis showed heterogeneous expression patterns of the hub genes across different cell types. APOE expression was mainly observed in CD8^+^ exhausted T cells and B cells (Fig. 8), whereas FN1 was highly expressed in fibroblasts and endothelial cells (Fig. 9). The single-cell expression patterns of the remaining hub genes are provided in Supplemental Digital Content 1, Supplemental Digital Content 1. Specifically, CSF2 showed its highest mean expression in fibroblasts; COL1A1 was predominantly expressed in fibroblasts and malignant cells; and ICAM1 showed relatively high expression in monocytes/macrophages, neutrophils, and fibroblasts. These findings provide exploratory cell-type-level context for the expression patterns of the identified hub genes.

### 3.9. Prediction of TCMs and component screening

Hub genes were input into the SymMap database, resulting in the identification of corresponding TCMs. APOE was associated with 66 TCMs, COL1A1 with 391 TCMs, CSF2 with 160 TCMs, FN1 with 95 TCMs, and ICAM1 with 425 TCMs. The full gene-specific TCM lists are provided in Supplemental Digital Content 2, Supplemental Digital Content 2. The intersection of these TCMs revealed 2 common TCMs associated with the hub genes: Ginseng (Ren Shen) and *Smilax glabra* (Tu Fu Ling). Using the TCM Systems Pharmacology Database and Analysis Platform database, 2 key active components were identified: Celabenzine and Diosgenin.

### 3.10. Molecular docking validation results

Molecular docking analysis was performed to explore the potential interactions between the hub gene-encoded proteins and the predicted active compounds from Ginseng and *S glabra*. In the computational docking model, lower binding energies indicated more favorable predicted ligand-target interactions. The docking results showed that the predicted active compounds had binding energies below 0 with the selected target proteins, suggesting potential binding affinity.

**Figure 10.**
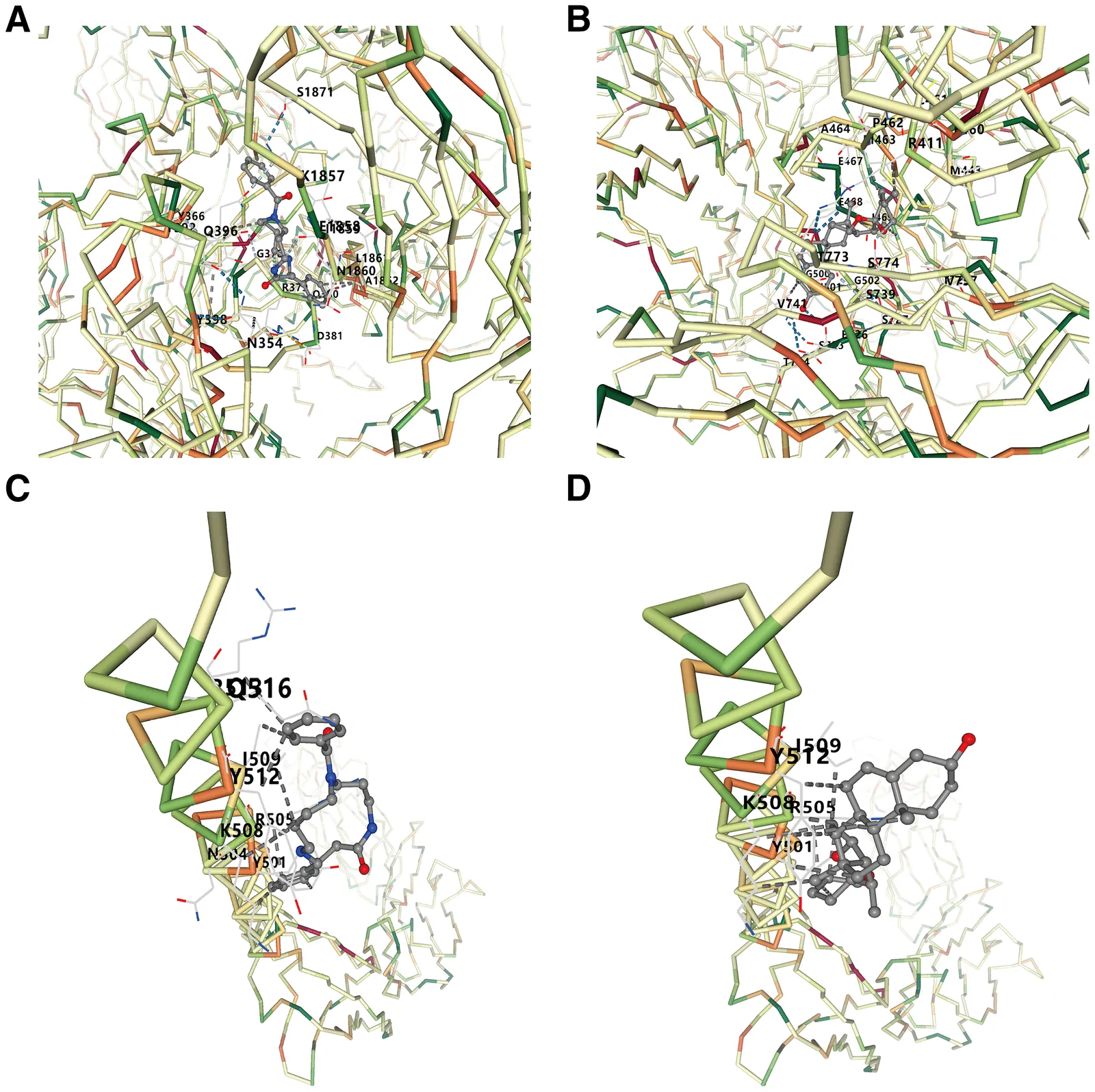
Molecular docking diagrams of hub genes and candidate compounds. (A) Predicted docking conformation of FN1 with the candidate active compound from Ginseng; (B) predicted docking conformation of FN1 with the candidate active compound from *S glabra*; (C) predicted docking conformation of ICAM1 with the candidate active compound from Ginseng; (D) predicted docking conformation of ICAM1 with the candidate active compound from *S glabra*. FN1 = fibronectin 1, ICAM = intercellular adhesion molecule.

Specifically, the binding scores of APOE with the predicted active compounds from Ginseng and *S glabra* were −7.6 and −8.0, respectively. The binding scores for COL1A1 were −6.2 and −6.3, and those for CSF2 were −6.7 and −8.3, respectively. The detailed docking scores and additional docking conformations for APOE, COL1A1, and CSF2 are provided in Supplemental Digital Content 3, Supplemental Digital Content 3. In addition, FN1 showed binding scores of −7.6 and −7.4 with the predicted active compounds from Ginseng and *S glabra*, respectively (Figs. 10A and 10B). ICAM1 showed binding scores of −9.0 and −8.7, respectively, indicating relatively favorable predicted interactions in the docking model (Figs. 10C and 10D). These findings provide preliminary computational evidence for possible compound-target interactions; however, they do not confirm biological activity or therapeutic efficacy and require further experimental validation.

## 4. Discussion

PTC is one of the most prevalent endocrine malignancies, contributing to approximately 40,000 deaths annually worldwide and representing a significant public health concern.^[7,8]^ Identifying biomarkers associated with PTC progression and assessing their diagnostic and therapeutic potential are crucial for enhancing patient prognosis and informing treatment strategies. In this study, CSF2, APOE, FN1, COL1A1, and ICAM1 were identified as key hub genes involved in PTC progression using data from the TCGA database. Further validation through survival analysis and immunohistochemical studies demonstrated that FN1 and COL1A1 are strongly correlated with the prognosis of PTC, underscoring their potential as prognostic biomarkers.

GO enrichment analysis indicates that the DEGs are primarily localized within the collagen-rich ECM and are involved in BP such as collagen catabolism and ECM organization. The tumor microenvironment of PTC is characterized by dense ECM composed of blood vessels, lymphatic vessels, and various cytokines and growth factors, all of which play critical roles in driving tumor initiation and progression.^[9]^ Research has demonstrated that the accumulation of fibrotic stroma, mainly produced by fibroblasts, accelerates the proliferation of connective tissue within tumors, leading to increased expression of α-smooth muscle actin, fibroblast activation protein, and other ECM-related proteins, thereby enhancing tumor invasiveness.^[10]^ Harach et al reported that significant fibrosis is present in 79% of invasive thyroid carcinomas, while the degree of fibrosis is markedly reduced in localized tumors.^[11]^ The immune microenvironment in thyroid cancer, similar to other tumors, is composed of various immune cells that critically influence tumorigenesis, growth, and prognosis. Studies have found that the extent of immune cell infiltration in thyroid tumor tissues is positively correlated with tumor size and the degree of lymph node metastasis, underscoring the significant role of immune cell infiltration and ECM in PTC progression.^[12,13]^

CSF2 is a hematopoietic growth factor that stimulates stem cells to produce granulocytes and monocytes.^[14]^ Research indicates that CSF2 and its associated signaling pathways play a dual role in modulating immune responses during tumor development and progression. On one hand, CSF2 can induce protective immunity in damaged tissues, thereby exerting an antiproliferative effect on tumors. Conversely, it can also lead to immune overactivation, which is associated with poor prognoses in various cancers.^[15,16]^ APOE, a key cholesterol transporter in the brain, is involved in several critical cellular processes, including neuroinflammation and amyloid-beta degradation and clearance.^[17]^ Studies have shown that APOE is overexpressed in various cancers, with higher levels of expression correlating with shorter patient survival. Additionally, APOE, as a highly specific and effective protein found in exosomes derived from M2 macrophages, can activate the phosphoinositide 3-kinase/protein kinase B signaling pathway. This activation facilitates intercellular transfer, leading to cytoskeletal remodeling and potentially contributing to tumor progression.^[18,19]^

FN1 is a glycoprotein involved in critical processes such as embryogenesis, wound healing, and blood coagulation. Extensive research has shown that FN1 plays a pivotal role in regulating interferon-gamma production through the natural killer cell p46-related protein receptor on natural killer cells, thereby controlling tumor architecture and metastasis. FN1 is implicated in the progression of multiple cancers, including oral squamous cell carcinoma, renal cancer, and thyroid cancer.^[20–22]^ COL1A1, a key component of type I collagen, is essential for the structural integrity of the ECM and connective tissues. Elevated expression of COL1A1 has been observed in various tumor tissues and cells, indicating its significant involvement in tumorigenesis.^[23]^ Furthermore, studies by Homan et al suggest that COL1A1 may influence tumor progression by affecting immune cell infiltration via its interaction with the ECM.^[24–27]^ ICAM1, expressed by tumor cells, regulates tumor progression through the promotion of tumor immune cell adhesion and signal transduction pathways. This regulation is integral to the development and spread of cancers such as oral squamous cell carcinoma, lung cancer, gastric cancer, and breast cancer.^[28,29]^

Contemporary pharmacological research has revealed that ginseng and its active components exhibit a broad spectrum of biological activities and potential therapeutic applications. Notably, ginsenosides have been reported to exert biphasic immunomodulatory effects, enhancing both nonspecific and specific immune responses in experimental models. These findings suggest that ginsenosides may have potential value as candidate compounds for immune-related disorders.^[30–32]^ Additionally, *S glabra*, a herb extensively used in traditional medicine, is renowned for its detoxifying, dampness-eliminating, and joint-relieving properties.^[33,34]^ Recent studies have further elucidated the bioactive properties of flavonoids, phenylpropanoids, and steroidal compounds in *S glabra*, suggesting their potential pharmacological effects in lowering uric acid, modulating inflammation and pain, exerting antibacterial activity, and affecting tumor-related BP.^[35–37]^ In this study, molecular docking results suggested that Celabenzine and Diosgenin exhibited favorable predicted binding affinities for ICAM1. These findings indicate possible compound-target interactions involving ICAM1; however, they do not directly demonstrate that these compounds can inhibit thyroid cancer cell invasion or metastasis.

ICAM1 is involved in cell adhesion, inflammatory signaling, and tumor-related BP. In PTC, increased ICAM1 expression has been associated with aggressive clinicopathological features, supporting its potential relevance to PTC progression.^[38]^ In addition, ICAM1-targeted antibody-drug conjugates have demonstrated antitumor activity in experimental models of papillary and anaplastic thyroid carcinoma, further supporting ICAM1 as a biologically relevant target for investigation.^[39]^ Functional studies in thyroid cancer cells have also shown that targeting ICAM1 through miR-335-5p can inhibit cell invasion and metastasis.^[40]^

In the present study, molecular docking suggested a potential interaction between Celabenzine and ICAM1. However, this finding represents a computational prediction only. Direct experimental evidence demonstrating that Celabenzine regulates ICAM1 expression or suppresses PTC progression is currently lacking. Therefore, the biological significance of the predicted Celabenzine–ICAM1 interaction requires validation in appropriate in vitro and in vivo models.

Diosgenin has been reported to exert anti-migratory and anti-invasive effects in experimental cancer models. In prostate cancer cells, diosgenin inhibited migration and invasion and affected signaling pathways including extracellular signal-regulated kinase, c-Jun N-terminal kinase, phosphoinositide 3-kinase/protein kinase B, and nuclear factor kappa B (NF-κB).^[41]^ Additional experimental evidence indicates that diosgenin can suppress protein kinase B/inhibitor of nuclear factor kappa-B kinase activation and NF-κB-regulated gene expression associated with proliferation and invasion.^[42]^ In vascular smooth muscle cells, diosgenin reduced tumor necrosis factor-alpha-induced adhesion molecule expression, including ICAM1, through modulation of mitogen-activated protein kinase, Akt, and NF-κB signaling.^[43]^ Diosgenin has also demonstrated anti-inflammatory activity involving CK2, c-Jun N-terminal kinase, NF-κB, activator protein 1, and inflammatory mediators in macrophage-based experimental models.^[44]^ These observations, together with broader evidence regarding the anticancer mechanisms of diosgenin,^[45]^ provide biological context for the docking findings of the present study. Nevertheless, these previous studies were conducted in experimental systems other than PTC, and the direct effects of diosgenin on PTC, particularly through ICAM1-mediated mechanisms, remain to be established experimentally.

ICAM1 may also contribute to immune cell trafficking within the thyroid tumor microenvironment. In the diffuse sclerosing variant of PTC, high endothelial venule-like vessels and adhesion-related mechanisms have been associated with lymphocyte recruitment, providing disease-specific context for the potential involvement of ICAM1 in immune cell interactions.^[46]^ Thus, the predicted interactions identified in the present docking analysis should be regarded as hypothesis-generating findings requiring further experimental validation.

Several limitations should be considered. First, this study was based primarily on publicly available datasets and computational analyses; therefore, the findings require independent experimental and clinical validation. Second, the single-cell analysis was used to provide exploratory cell-type-level context and was based on GSE148673, which includes multiple tumor types rather than a dedicated PTC cohort. Third, the molecular docking results represent predicted ligand-target interactions and cannot establish biological activity or therapeutic efficacy. Accordingly, the generalizability of these findings beyond the analyzed public datasets and computational models should be interpreted cautiously.

This study identified CSF2, APOE, FN1, COL1A1, and ICAM1 as hub genes associated with PTC through TCGA-based bioinformatics analysis. Among them, FN1 and COL1A1 showed potential prognostic relevance and were further supported by immunohistochemical analysis, while single-cell analysis provided exploratory cell-type-level context for the identified hub genes. Immune infiltration analysis suggested that these hub genes may be related to the tumor immune microenvironment of PTC. In addition, TCM compound prediction and molecular docking provided preliminary computational evidence for possible compound-target interactions. Further experimental and clinical validation is required to confirm the biological functions of the identified genes and the pharmacological relevance of the predicted compounds.

## Acknowledgments

This study was supported by the Heilongjiang University of Chinese Medicine “Outstanding Innovative Talent Support Program.”

## Author contributions

**Validation:** Qiuyang Wei.

**Visualization:** Ke Meng.

**Writing – original draft:** Dong Yin.

**Writing – review & editing:** Likun Du.






