# Supplementary material for: TCGA-based identification of prognostic biomarkers and candidate traditional Chinese medicine compounds in papillary thyroid carcinoma: An observational study
Source: Medicine (Baltimore). 2026 Sep 4;105(36):e50562. doi: 10.1097/MD.0000000000050562 (PMC13549628; doi:10.1097/MD.0000000000050562)
Supplement: Supplementary file 1 [file medi-105-e50562-s001.pdf]

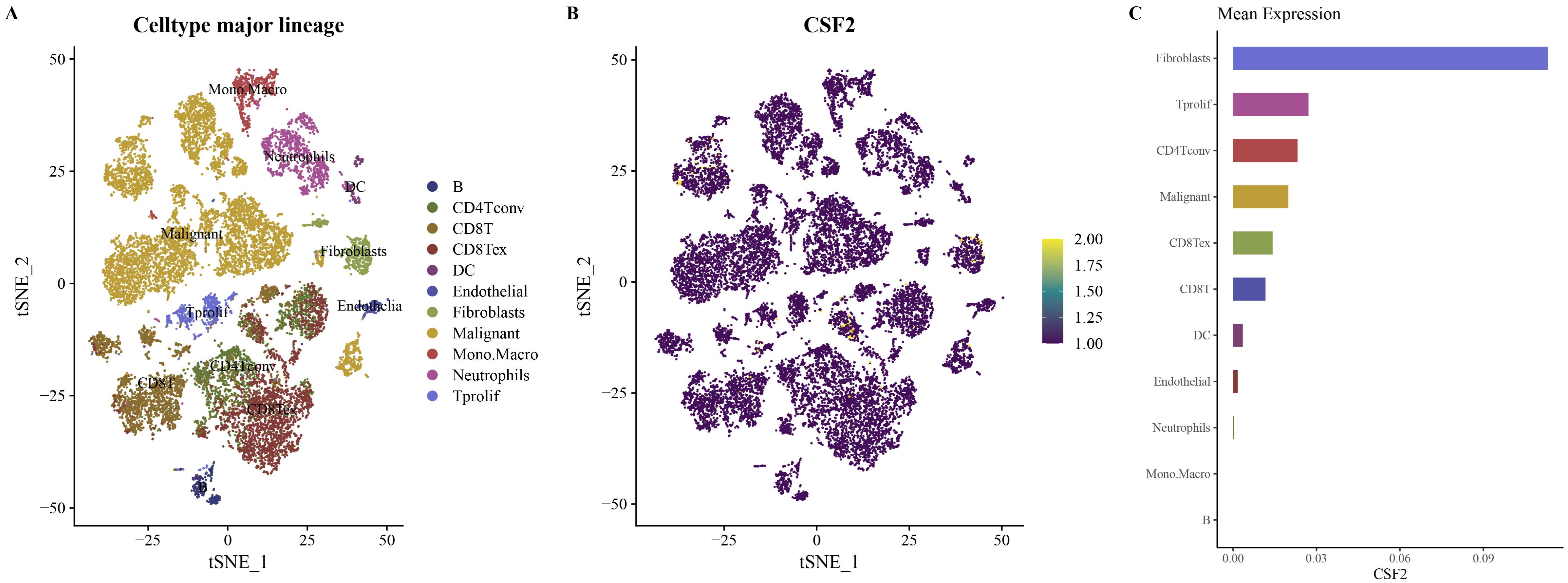

A. Single-cell expression pattern of CSF2

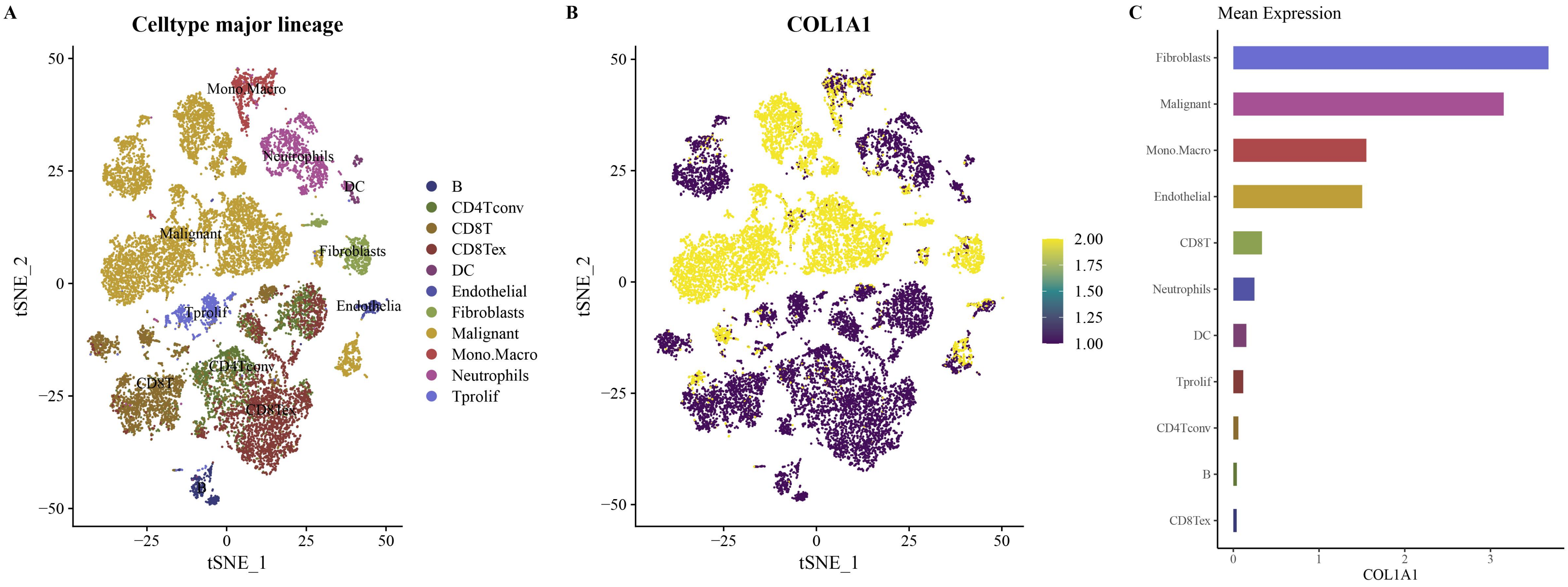

B. Single-cell expression pattern of COL1A1

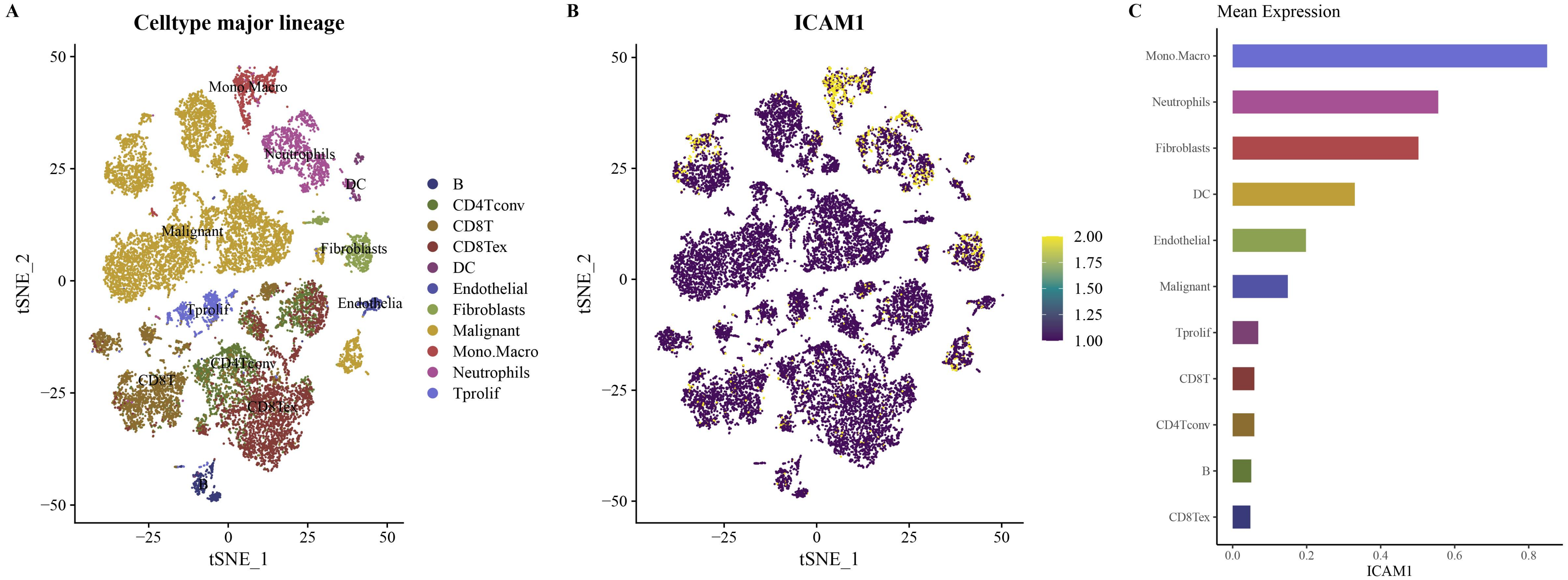

C. Single-cell expression pattern of ICAM1
