# Supplementary material for: TCGA-based identification of prognostic biomarkers and candidate traditional Chinese medicine compounds in papillary thyroid carcinoma: An observational study
Source: Medicine (Baltimore). 2026 Sep 4;105(36):e50562. doi: 10.1097/MD.0000000000050562 (PMC13549628; doi:10.1097/MD.0000000000050562)
Supplement: Supplementary file 2 [file medi-105-e50562-s002.pdf]

Supplemental Digital Content 2. Traditional Chinese medicine correspondence results for hub genes

This file contains five sequential sections corresponding to APOE, COL1A1, CSF2, FN1, and ICAM1.

S2A. APOE corresponding traditional Chinese medicines

Columns are reproduced from the source workbook. P_value and FDR columns report the SymMap statistical output; relationship and evidence score describe inferred gene-herb evidence.

Herb id	Chinese name	Pinyin name	Latin name	English name	Class in Chinese	Class in English	P_value	FDR(BH)	FDR(Bonferroni)	Relationship	Inferred evidence score
SMHB00008	菝葜	Baqia	Smilacis Chinae Rhizoma	Smilax Chi	祛风湿散寒药	Wind-Dampnessdispelling And Colddispersing Medicinal	8.466e-04	0.0142394	1	By_ingredient	0.4059
SMHB00009	白扁豆	Baibiandou	Lablab Semen Album	White Hyacinth Bean	补气药	Qi Reinforcing Drugs	0.00326858	0.0307564	1	By_ingredient	0.4259
SMHB00010	白附子	Baifuzi	Typhonii Rhizoma	rhizome of Giant Typhonium	化痰药	Phlegresolving Medicine	0.0024387	0.0261663	1	By_ingredient	0.3945
SMHB00011	白果	Baiguo	Ginkgo Semen	Ginkgo seed	止咳平喘药	Antitussive Antiasthmetics	0.00634553	0.0397671	1	By_ingredient	0.4309
SMHB00013	白花蛇舌草	Baihuasheshecao	Herba Hedyotis Diffusae	all - grass of Spreading Hedyotis	清热解毒药	Antipyretic Detoxicate Drugs	0.00320549	0.0304356	1	By_ingredient	0.4028
SMHB00028	百部	Baibu	Stemonae Radix	Root of Japanese Stemo	止咳平喘药	Antitussive Antiasthmetics	0.013437	0.0525122	1	By_ingredient	0.3998
SMHB00066	赤小豆	Chixiaodou	Vignae Semen	Rice Bean	利水渗湿药	Diuretic Dampness Excreting Drugs	0.0389653	0.0804306	1	By_ingredient	0.1816
SMHB00070	川贝母	Chuanbeimu	Fritillariae Cirrhosae Bulbus	Bulb of Tendrilleaf Fritillary	化痰药	Phlegresolving Medicine	0.00907805	0.0457888	1	By_ingredient	0.3436
SMHB00071	川槿皮	Chuanjinpi	None	Cortex Hibisci	清热解毒药	Antipyretic Detoxicate Drugs	0.0324369	0.0744111	1	By_ingredient	0.1874
SMHB00074	川牛膝	Chuanniuxi	Cyathulae Radix	root of Medicil cyathula	活血祛瘀药	Blood Activating Stasis Removing Drugs	0.0414026	0.0830129	1	By_ingredient	0.1821
SMHB00094	淡豆豉	Dandouchi	Sojae Semen Praeparatum	Fermented Soybean	辛凉解表药	Pungent Cool Diaphoretics	0.00116986	0.0172508	1	By_ingredient	0.3685
SMHB00097	刀豆	Daodou	Cana v Aliae Semen	Jack Bean	理气药	Qi Regulating Drugs	0.0332553	0.0750258	1	By_ingredient	0.1622
SMHB00110	冬瓜子	Dongguazi	None	Benincasae Semen	化痰药	Phlegresolving Medicine	0.00238385	0.0257778	1	By_ingredient	0.4174
SMHB00121	翻白草	Fanbaicao	Potentillae Discoloris Herba	all-grass of discolor cinquefoil	清热解毒药	Antipyretic Detoxicate Drugs	0.00238385	0.0257778	1	By_ingredient	0.3669
SMHB00139	葛花	Gehua	Flos Puerariae Lobatae	flower bud of lobed kudzuvine	辛凉解表药	Pungent Cool Diaphoretics	0.014424	0.0542023	1	By_ingredient	0.3417
SMHB00152	广枣	Guangzao	Choerospondiatis Fructus	fruit of Axillary choerospondias	活血祛瘀药	Blood Activating Stasis Removing Drugs	0.00714558	0.0417125	1	By_ingredient	0.3436
SMHB00155	桂枝	Guizi	Cinnamomi Ramulus	Cassia Twig	辛温解表药	Pungent-Warm Exterior-Releasing Medicinal	0.0240104	0.0656908	1	By_ingredient	0.3733
SMHB00156	海风藤	Haifengteng	Piperis Kadsurae Caulis	stem of Kadsura Pepper	祛风湿散寒药	Wind-Dampnessdispelling And Colddispersing Medicinal	0.00280655	0.0142808	1	By_ingredient	0.1913
SMHB00158	海桐皮	Haitongpi	Cortex Erythrie	bark of Oriental Variegated Coralbean	祛风湿清热药	Wind-Dampnessdispelling And Heatclearing Medicinal	0.0430242	0.084586	1	By_ingredient	0.1704
SMHB00177	虎杖	Huzhang	Polygoni Cuspidati Rhizoma Et Radix	rhizome of Gaint Knotweed	利水渗湿药	Diuretic Dampness Excreting Drugs	0.00837773	0.044321	1	By_ingredient	0.3418
SMHB00179	花生衣	Huashengyi	Testa Arachidis Hypogaeae	Semino-dermis Arachidis	收敛止血药	Astringent Hemostatic Medicinal	3.538e-04	0.00787646	1	By_ingredient	0.3113
SMHB00191	鸡骨草	Jigucao	Abri Herba	Canton Love-pea Vine	利水渗湿药	Diuretic Dampness Excreting Drugs	0.0027045	0.0139812	1	By_ingredient	0.1806
SMHB00193	鸡屎藤	Jishiteng	None	Paederiae Scandentis Herba	消食药	Digestants	0.00558956	0.0381237	1	By_ingredient	0.2979
SMHB00230	苦杏仁	Kuxingren	Armeniacae Semen Amarum	bitter Apricot seed	止咳平喘药	Antitussive Antiasthmetics	0.00887536	0.0452364	1	By_ingredient	0.3218
SMHB00235	老鹳草	Laoguancao	Erodii Herba Geranii Herba	All - grass of Common Heron's bill	祛风湿散寒药	Wind-Dampnessdispelling And Colddispersing Medicinal	0.043834	0.0853487	1	By_ingredient	0.1679
SMHB00238	雷丸	Leiwan	Omphalia	Thunder Ball	驱虫药	Antiparasitic Drugs	0.0275128	0.0696765	1	By_ingredient	0.1662
SMHB00246	两头尖	Liangtoujian	Anemones Raddeanae Rhizoma	Radde Anemone Rhizome	祛风湿散寒药	Wind-Dampnessdispelling And Colddispersing Medicinal	0.0422137	0.0837456	1	By_ingredient	0.1916
SMHB00258	路路通	Lulutong	Liquidambaris Fructus	fruit of beautiful sweetgum	祛风湿散寒药	Wind-Dampnessdispelling And Colddispersing Medicinal	0.0291568	0.0710566	1	By_ingredient	0.201
SMHB00274	蔓荆子	Manjingzi	Viticis Fructus	Shrub Chastetree Fruit	辛凉解表药	Pungent Cool Diaphoretics	4.44E-05	0.00181969	1	By_ingredient	0.7721
SMHB00295	南瓜子	nguazi	Semen Cucurbitae Moschatae	Pumpkin seed and husks	驱虫药	Antiparasitic Drugs	0.0381516	0.0797513	1	By_ingredient	0.1896
SMHB00306	女贞子	Nüzhenzi	Ligustri Lucidi Fructus	fruit of Glossy privet	补阴药	Yin-Tonifying Medicinal	0.0490662	0.0900568	1	By_ingredient	0.4254
SMHB00311	平贝母	Pingbeimu	Fritillariae Ussuriensis Bulbus	Ussuri Fritillary Bulb	化痰药	Phlegresolving Medicine	0.0486792	0.0895448	1	By_ingredient	0.1955
SMHB00319	前胡	Qianhu	Peucedani Radix	root of Grand Hogfennel	化痰药	Phlegresolving Medicine	0.0101225	0.0474481	1	By_ingredient	0.3312
SMHB00332	青叶胆	Qingyedan	Swertiae Mileensis Herba	All - grass of Mile Swertia	利水渗湿药	Diuretic Dampness Excreting Drugs	0.0405908	0.082294	1	By_ingredient	0.1906
SMHB00336	人参	Renshen	Ginseng Radix Et Rhizoma	Ginseng	补气药	Qi Reinforcing Drugs	0.00656366	0.0402375	1	By_ingredient	0.5695
SMHB00342	蕤仁	Ruiren	Prinsepiae Nux	Hedge Prinsepia Nut	辛凉解表药	Pungent Cool Diaphoretics	0.0430242	0.084586	1	By_ingredient	0.1759
SMHB00355	砂仁	Sharen	Amomi Fructus	Villous Amomum Fruit	化湿药	Dampness Removing Drugs	0.0265609	0.068552	1	By_ingredient	0.3122
SMHB00382	松花粉	Songhuafen	Pini Pollen	Pine Pollen	收敛止血药	Astringent Hemostatic Medicinal	0.0175928	0.0585289	1	By_ingredient	0.1813
SMHB00384	松子仁	Songziren	None	Semen Pini Koraiensis	润下药	Laxatives	0.00589743	0.0388881	1	By_ingredient	0.1654
SMHB00391	天冬	Tiandong	Asparagi Radix	Cochinchinese Asparagus Root	补阴药	Yin-Tonifying Medicinal	0.0446432	0.0862047	1	By_ingredient	0.1793
SMHB00401	铁线透骨草	Tiexiantougucao	None	Herba Clematidis Aethusaefoliae	祛风湿散寒药	Wind-Dampnessdispelling And Colddispersing Medicinal	0.0225649	0.0647543	1	By_ingredient	0.1634
SMHB00403	通草	Tongcao	Tetrapanacis Medulla	puth of Ricepaperplant	利水渗湿药	Diuretic Dampness Excreting Drugs	0.034073	0.0757806	1	By_ingredient	0.2041
SMHB00404	土贝母	Tubeimu	Bolbostemmatis Rhizoma	Paniculate Bolbostemma	化痰药	Phlegresolving Medicine	0.0486792	0.0895448	1	By_ingredient	0.1987
SMHB00405	土茯苓	Tufuling	Smilacis Glabrae Rhizoma	Glabrous Greenbrier Rhizome	清热解毒药	Antipyretic Detoxicate Drugs	3.04E-06	2.279e-04	1	By_ingredient	0.7994
SMHB00409	瓦松	Wasong	Orostachyis Fimbriatae Herba	Cotyledon Fimbriata Turcz.	清热凉血药	Heat Clearing Blood Cooling Drugs	0.0242168	0.0658678	1	By_ingredient	0.1728
SMHB00410	王不留行	Wangbuliuxing	Vaccariae Semen	Vacarria seed, Cow soapwort seed	活血祛瘀药	Blood Activating Stasis Removing Drugs	0.0430242	0.084586	1	By_ingredient	0.1547
SMHB00438	小蓟	Xiaoji	Cirsii Herba	Field Thistle Herb	凉血止血药	Blood-Cooling Hemostatic Medicinal	0.043834	0.0853487	1	By_ingredient	0.1697
SMHB00443	续断	Xuduan	Dipsaci Radix	Himalayan Teasel Root	补阳药	Yang Reinforcing Drugs	0.03489	0.0765633	1	By_ingredient	0.1765
SMHB00445	玄参	Xuanshen	Scrophulariae Radix	Figwort Root	清热凉血药	Heat Clearing Blood Cooling Drugs	0.00511039	0.0364409	1	By_ingredient	0.3906
SMHB00447	寻骨风	Xungufeng	None	Rhizoma Seu Herba Aristolochiae Mollissimae	祛风湿散寒药	Wind-Dampnessdispelling And Colddispersing Medicinal	0.0250418	0.0670179	1	By_ingredient	0.1867
SMHB00450	亚麻子	Yamazi	Lini Semen	Linseed	润下药	Laxatives	0.0316179	0.0736315	1	By_ingredient	0.1739
SMHB00457	益智	Yizhi	Alpiniae Oxyphyllae Fructus	Alpiniae Oxyphyliae Fructus	补阳药	Yang Reinforcing Drugs	0.034073	0.0757806	1	By_ingredient	0.173
SMHB00471	预知子	Yuzhizi	Akebiae Fructus	Akebia Fruit	理气药	Qi Regulating Drugs	0.0454517	0.0870149	1	By_ingredient	0.186
SMHB00477	皂角刺	Zhaojiaoci	Gleditsiae Spina	spine of Chinese Honeylocust	化痰药	Phlegresolving Medicine	0.0357064	0.0771677	1	By_ingredient	0.1761
SMHB00487	肿节风	Zhongjiefeng	Sarcandrae Herba	all-grass of Glabrous sarcandra	清热解毒药	Antipyretic Detoxicate Drugs	0.0397784	0.0813496	1	By_ingredient	0.1793
SMHB00489	猪牙皂	Zhuyazao	Gleditsiae Fructus Abnormalis	Chinese Honeylocust Abnormal Fruit	化痰药	Phlegresolving Medicine	0.0125967	0.0514001	1	By_ingredient	0.2004
SMHB00495	紫花前胡	Zihuaqianhu	Peucedani Decursivi Radix	Peucedanidecursivi Radix	化痰药	Phlegresolving Medicine	0.0422137	0.0837456	1	By_ingredient	0.1732
SMHB00509	大豆黄卷	Dadouhuangjuan	Sojae Semen Germinatum	None	辛凉解表药	Pungent Cool Diaphoretics	0.00168838	0.0216442	1	By_ingredient	0.1772
SMHB00512	山麦冬	Shanmaidong	Liriopes Radix	None	补阴药	Yin-Tonifying Medicinal	0.0184232	0.0594676	1	By_ingredient	0.1768
SMHB00531	毛诃子	Maohezi	Terminaliae Belliricae Fructus	None	清热解毒药	Antipyretic Detoxicate Drugs	0.0134311	0.0525122	1	By_ingredient	0.2002
SMHB00589	何首乌	Heshouwu	Polygoni Multiflori Radix	None	补虚药	Tonifying And Replenishing Medicinal	0.00558956	0.0381237	1	By_ingredient	0.3875
SMHB00639	粉葛	Fenge	Puerariae Thomsonii Radix	None	辛凉解表药	Pungent Cool Diaphoretics	0.00253155	0.026713	1	By_ingredient	0.1604
SMHB00644	桑螵蛸	Sangpiaoxiao	Mantidis Ootheca	None	收涩药	Astringent Medicinal	0.0242168	0.0658678	1	By_ingredient	0.1795
SMHB00658	鹿茸	Lurong	Cervi Cornu Pantotrichum	None	补虚药	Tonifying And Replenishing Medicinal	0.0200819	0.0612974	1	By_ingredient	0.1731
SMHB00659	羚羊角	Lingyangpao	Saigae Tataricae Cornu	None	平肝熄风药	Liver-Pacifying Wind-Extinguishing Medicinal	0.0167618	0.0576709	1	By_ingredient	0.2237
SMHB00702	麝香	Shexiang	Moschus	None	开窍药	Resuscitative Stimulant; Resuscitative Medicinal	0.0430242	0.084586	1	By_ingredient	0.1838

S2B. COL1A1 corresponding traditional Chinese medicines

Columns are reproduced from the source workbook. P_value and FDR columns report the SymMap statistical output; relationship and evidence score describe inferred gene-herb evidence.

Herb id	Chinese name	Pinyin name	Latin name	English name	Class in Chinese	Class in English	P_value	FDR(BH)	FDR(Bonferroni)	Relationship	Inferred evidence score
SMHB00001	矮地茶	Aidicha	Ardisiae Japonicae Herba	Japanese Ardisia Herb	止咳平喘药	Antitussive Antiasthmetics	0.140591	0.166542	1	By_ingredient	0.1676
SMHB00002	艾叶	Aiye	Artemisiae Argyi Folium	leaf of Argy Wormwood	温经止血药	Channel-Warming Hemostatic	0.0116123	0.0287506	1	By_ingredient	0.2959
SMHB00004	八角茴香	Bajiaohuixiang	Anisi Stellati Fructus	Chinese Star Anise	温里药	Warming Interior Drugs	0.00356878	0.0315793	1	By_ingredient	0.3345
SMHB00005	八角莲	Bajiaolian	None	Dysosmae Verspiellis Rhixoma Et Radix	清热解毒药	Antipyretic Detoxicate Drugs	0.0599845	0.0831226	1	By_ingredient	0.1592
SMHB00006	巴豆	Badou	Crotonis Fructus	Croton Fruit	攻下药	Offensive Purgative Medicinal	0.041646	0.0636079	1	By_ingredient	0.1595
SMHB00007	巴戟天	Bajitian	Morindae Officinalis Radix	Morindae Officilis Radix	补阳药	Yang Reinforcing Drugs	0.114262	0.139737	1	By_ingredient	0.1598
SMHB00008	菝葜	Baqia	Smilacis Chinae Rhizoma	Smilax Chi	祛风湿散寒药	Wind-Dampnessdispelling And Colddispersing Medicinal	0.0275143	0.0481841	1	By_ingredient	0.1671
SMHB00009	白扁豆	Baibiandou	Lablab Semen Album	White Hyacinth Bean	补气药	Qi Reinforcing Drugs	3.102e-04	0.00723165	1	By_ingredient	0.4909
SMHB00010	白附子	Baifuzi	Typhonii Rhizoma	rhizome of Giant Typhonium	化痰药	Phlegresolving Medicine	0.0536978	0.0764999	1	By_ingredient	0.1638
SMHB00011	白果	Baiguo	Ginkgo Semen	Ginkgo seed	止咳平喘药	Antitussive Antiasthmetics	3.46E-05	0.00150964	1	By_ingredient	0.702
SMHB00013	白花蛇舌草	Baihuasheshecao	Herba Hedyotis Diffusae	all - grass of Spreading Hedyotis	清热解毒药	Antipyretic Detoxicate Drugs	0.0300979	0.0510184	1	By_ingredient	0.15
SMHB00015	白蔹	Bailian	Ampelopsis Radix	root of Japanese Ampelopsis	清热解毒药	Antipyretic Detoxicate Drugs	0.00190036	0.011285	1	By_ingredient	0.3104
SMHB00018	白茅根	Baimaogen	Imperatae Rhizoma	Lalang Grass Rhizome	凉血止血药	Blood-Cooling Hemostatic Medicinal	0.0581026	0.0811566	1	By_ingredient	0.1628
SMHB00019	白前	Baiqian	Cynanchi Stauntonii Rhizoma Et Radix	Cynchum root	化痰药	Phlegresolving Medicine	0.0397301	0.061471	1	By_ingredient	0.1705
SMHB00020	白屈菜	Baiqucai	Chelidonii Herba	Chelidonii Herba	止咳平喘药	Antitussive Antiasthmetics	0.0422838	0.0642556	1	By_ingredient	0.1814
SMHB00021	白芍	Baishao	Paeoniae Radix Alba	White peony root	补血药	Blood-Tonifying Medicinal	0.129564	0.155314	1	By_ingredient	0.1674
SMHB00022	白术	Baizhu	Atractylodis Macrocephalae Rhizoma	rhizome of Largehead Atractylodes	补气药	Qi Reinforcing Drugs	0.0767667	0.100731	1	By_ingredient	0.1691
SMHB00023	白头翁	Baitouweng	Pulsatillae Radix	Root of Chinese Pulsatilla	清热解毒药	Antipyretic Detoxicate Drugs	0.061863	0.0850873	1	By_ingredient	0.1911
SMHB00024	白薇	Baiwei	Cynanchi Atrati Radix Et Rhizoma	Blackend Swallowwort Root	清虚热药	Asthenic Heat Dispelling Drugs	0.063738	0.08701	1	By_ingredient	0.1625
SMHB00025	白鲜皮	Baixianpi	Dictamni Cortex	Dictamni Cortex	清热燥湿药	Heat-Clearing And Dampnessdrying Medicinal	0.0674777	0.0910188	1	By_ingredient	0.1466
SMHB00027	白芷	Baizhi	Angelicae Dahuricae Radix	Dahurian Angelica Root	辛温解表药	Pungent-Warm Exterior-Releasing Medicinal	0.0118788	0.0291326	1	By_ingredient	0.3136
SMHB00030	柏子仁	Baiziren	Platycladi Semen	seed of Chinese Arborvitae	安神药	Tranguilizing Medicinal	0.00784935	0.0435328	1	By_ingredient	0.3248
SMHB00031	败酱草	Baijiangcao	Herba Patriniae	All - grass of Dahurian Patrinia	清热解毒药	Antipyretic Detoxicate Drugs	7.755e-04	0.00605911	1	By_ingredient	0.3054
SMHB00032	斑蝥	Banmao	Mylabris	Mylabris	活血祛瘀药	Blood Activating Stasis Removing Drugs	0.0210274	0.0405202	1	By_ingredient	0.1694
SMHB00033	板蓝根	Banlangen	Isatidis Radix	Root of Dyers Woad.	清热解毒药	Antipyretic Detoxicate Drugs	4.400e-04	0.0040596	1	By_ingredient	0.4912
SMHB00034	半边莲	Banbianlian	Lobeliae Chinensis Herba	Chinese Lobelia Herb	清热解毒药	Antipyretic Detoxicate Drugs	0.00171553	0.0105975	1	By_ingredient	0.3012
SMHB00035	半夏	Banxia	Pinelliae Rhizoma	tuber of Pinellia	化痰药	Phlegresolving Medicine	0.00370147	0.0163592	1	By_ingredient	0.298
SMHB00036	半枝莲	Banzhilian	Scutellariae Barbatae Herba	all - grass of Barbed Skullcap	清热解毒药	Antipyretic Detoxicate Drugs	0.00190036	0.011285	1	By_ingredient	0.3051
SMHB00037	薄荷	Bohe	Menthae Haplocalycis Herba	Mentha, Peppermint	辛凉解表药	Pungent Cool Diaphoretics	9.187e-04	0.0147947	1	By_ingredient	0.6656
SMHB00040	北沙参	Beishashen	Glehniae Radix	root of Coastal Glehnia	补阴药	Yin-Tonifying Medicinal	0.0724425	0.0962893	1	By_ingredient	0.1396
SMHB00041	五味子	Wuweizi	Schisandrae Chinensis Fructus	Schisandrae Chinensis Fructus	收涩药	Astringent Medicinal	0.0896278	0.114132	1	By_ingredient	0.1485
SMHB00042	荜茇	Bibo	Piperis Longi Fructus	Piperis Longi Fructus	温里药	Warming Interior Drugs	0.0773829	0.101427	1	By_ingredient	0.1632
SMHB00043	荜澄茄	Bichengqie	Litseae Fructus	Litseae Fructus	温里药	Warming Interior Drugs	5.287e-04	0.00459609	1	By_ingredient	0.3455
SMHB00044	蓖麻子	Bimazi	Ricini Semen	Castor Seed	拔毒化腐生肌药	External Medicinal (Draw Out Toxin, Resolve Putridity)	0.038451	0.0601681	1	By_ingredient	0.1556
SMHB00045	萹蓄	Bianxu	Polygoni Avicularis Herba	all-grass of common knotgrass	利水渗湿药	Diuretic Dampness Excreting Drugs	0.00487066	0.0188222	1	By_ingredient	0.3017
SMHB00047	槟榔	Binglang	Arecae Semen	areca seed	驱虫药	Antiparasitic Drugs	0.0100491	0.0474436	1	By_ingredient	0.3462
SMHB00049	布渣叶	Buzhaye	Microctis Folium	Microctis Folium	拔毒化腐生肌药	External Medicinal (Draw Out Toxin, Resolve Putridity)	3.446e-04	0.00344306	1	By_ingredient	0.2995
SMHB00051	苍耳子	Cang'erzi	Xanthii Fructus	fruit of Siberian Cockleblur	辛温解表药	Pungent-Warm Exterior-Releasing Medicinal	0.00257049	0.0269374	1	By_ingredient	0.4872
SMHB00054	草果	Caoguo	Tsaoko Fructus	Tsaoko	化湿药	Dampness Removing Drugs	0.00399126	0.032859	1	By_ingredient	0.3422
SMHB00055	草乌	Caowu	Aconiti Kusnezoffii Radix	Kusnezoff Monkshood Root	祛风湿散寒药	Wind-Dampnessdispelling And Colddispersing Medicinal	0.0242758	0.0442744	1	By_ingredient	0.1886
SMHB00057	侧柏叶	Cebaiye	Platycladi Cacumen	leafy twigs of Oriental Arborvitae	凉血止血药	Blood-Cooling Hemostatic Medicinal	0.00255258	0.0135453	1	By_ingredient	0.4884
SMHB00058	柴胡	Chaihu	Bupleuri Radix	root of Chinese Thorowax	辛凉解表药	Pungent Cool Diaphoretics	0.0224888	0.0422319	1	By_ingredient	0.3137
SMHB00060	常山	Changshan	Dichroae Radix	root of antifebrile Dichroa	涌吐药	Emetic Medicinal	0.0643623	0.0876099	1	By_ingredient	0.1611
SMHB00061	车前草	Cheqiancao	Plantaginis Herba	all-grass of Rippleseed plantain	利水渗湿药	Diuretic Dampness Excreting Drugs	3.764e-04	0.00823271	1	By_ingredient	0.6447
SMHB00062	车前子	Cheqianzi	Plantaginis Semen	seed of Asiatic pantain	利水渗湿药	Diuretic Dampness Excreting Drugs	0.0390907	0.0608393	1	By_ingredient	0.14
SMHB00063	沉香	Chenxiang	Aquilariae Lignum Resinatum	Oily wood of Agalloch Eaglewood	理气药	Qi Regulating Drugs	0.00652432	0.0215933	1	By_ingredient	0.6168
SMHB00064	陈皮	Chenpi	Citri Reticulatae Pericarpium	Dried Tangerine Peel	理气药	Qi Regulating Drugs	6.66E-05	0.00245258	1	By_ingredient	0.6684
SMHB00065	赤芍	Chishao	Paeoniae Radix Rubra	Red Peony root	清热凉血药	Heat Clearing Blood Cooling Drugs	0.00364957	0.0162566	1	By_ingredient	0.3326
SMHB00069	楮实子	Chushizi	Broussonetiae Fructus	Broussonetiae Fructus	补阴药	Yin-Tonifying Medicinal	8.214e-04	0.013924	1	By_ingredient	0.5319
SMHB00070	川贝母	Chuanbeimu	Fritillariae Cirrhosae Bulbus	Bulb of Tendrilleaf Fritillary	化痰药	Phlegresolving Medicine	0.00475283	0.0186294	1	By_ingredient	0.3174
SMHB00071	川槿皮	Chuanjinpi	None	Cortex Hibisci	清热解毒药	Antipyretic Detoxicate Drugs	0.00118868	0.0172508	1	By_ingredient	0.3548
SMHB00072	川楝子	Chuanlianzi	Toosendan Fructus	Szechwan Chiberry Fruit	理气药	Qi Regulating Drugs	6.784e-04	0.0123388	1	By_ingredient	0.5032
SMHB00073	川木通	Chuanmutong	Clematidis Armandii Caulis	Armand Clematis Stem	利水渗湿药	Diuretic Dampness Excreting Drugs	4.28E-05	0.00177154	1	By_ingredient	0.7611
SMHB00074	川牛膝	Chuanniuxi	Cyathulae Radix	root of Medicil cyathula	活血祛瘀药	Blood Activating Stasis Removing Drugs	4.318e-04	0.00401156	1	By_ingredient	0.3143
SMHB00075	川射干	Chuanshegan	Iridis Tectori Rhizoma	Iridis Tectori Rhizoma	清热解毒药	Antipyretic Detoxicate Drugs	0.0244922	0.0663191	1	By_ingredient	0.3627
SMHB00076	川乌	Chuanwu	Aconiti Radix	root of Common Monkshood	祛风湿散寒药	Wind-Dampnessdispelling And Colddispersing Medicinal	2.016e-04	0.00540337	1	By_ingredient	0.8406
SMHB00077	川芎	Chuanxiong	Chuanxiong Rhizoma	Chuanxiong rhizome, Szechuan lovage root	活血祛瘀药	Blood Activating Stasis Removing Drugs	0.00772573	0.0235781	1	By_ingredient	0.529
SMHB00079	穿心莲	Chuanxinlian	Andrographis Herba	all-grass of Common Androgrphis	清热解毒药	Antipyretic Detoxicate Drugs	0.0612372	0.0844632	1	By_ingredient	0.1564
SMHB00080	垂盆草	Chuipencao	Sedi Herba	all - grass of Stringy stonecrop	清热解毒药	Antipyretic Detoxicate Drugs	7.29E-05	0.00260279	1	By_ingredient	0.7219
SMHB00081	椿皮	Chunpi	Ailanthi Cortex	Ailanthus bark, Tree of heaven bark	清热燥湿药	Heat-Clearing And Dampnessdrying Medicinal	0.0599845	0.0831226	1	By_ingredient	0.1563
SMHB00083	大风子	Dafengzi	None	Hydnocarpi Anthelminticae Semen	解毒杀虫燥湿止痒药	Medicinal For Detoxification、Parasiticide、Drying Dampness And Relieving Itching	0.0288069	0.0495649	1	By_ingredient	0.1465
SMHB00086	大蓟	Daji	Cirsii Japonici Herba	all-grass of Thistle	凉血止血药	Blood-Cooling Hemostatic Medicinal	0.031178	0.0729984	1	By_ingredient	0.3006
SMHB00087	大青叶	Daqingye	Isatidis Folium	Isatis leaf	清热解毒药	Antipyretic Detoxicate Drugs	0.00876196	0.0449461	1	By_ingredient	0.3343
SMHB00090	大枣	Dazao	Jujubae Fructus	Jujube Chinese date	补气药	Qi Reinforcing Drugs	1.386e-04	0.00183239	1	By_ingredient	0.6601
SMHB00091	大皂角	Dazaojiao	Gleditsiae Sinensis Fructus	Gleditsiae Fructus	化痰药	Phlegresolving Medicine	0.0993154	0.12425	1	By_ingredient	0.1415
SMHB00092	玳玳花	Daidaihua	None	Citrus Aurantium	理气药	Qi Regulating Drugs	0.108908	0.134201	1	By_ingredient	0.151
SMHB00093	丹参	Danshen	Salviae Miltiorrhizae Radix Et Rhizoma	root of Ligulilobe sage	活血祛瘀药	Blood Activating Stasis Removing Drugs	1.08E-05	6.181e-04	1	By_ingredient	1.0073
SMHB00095	当归	Danggui	Angelicae Sinensis Radix	root of Chinese Angelica	补血药	Blood-Tonifying Medicinal	6.967e-04	0.0125588	1	By_ingredient	0.7059
SMHB00096	党参	Dangshen	Codonopsis Radix	root Pilose Asiabell	补气药	Qi Reinforcing Drugs	0.00627128	0.0395886	1	By_ingredient	0.5341
SMHB00098	倒扣草	Daokoucao	None	Achyranthis Asperae Herba	活血祛瘀药	Blood Activating Stasis Removing Drugs	0.049274	0.0716808	1	By_ingredient	0.1695
SMHB00100	地耳草	Diercao	Herba Hyperici Japonici	all - grass of Japanese St. Johnswort	利水渗湿药	Diuretic Dampness Excreting Drugs	6.59E-05	0.00109954	1	By_ingredient	0.5224
SMHB00102	地肤子	Difuzi	Kochiae Fructus	fruit of Belvedere	利水渗湿药	Diuretic Dampness Excreting Drugs	0.00356878	0.0315793	1	By_ingredient	0.3141
SMHB00103	地骨皮	Digupi	Lycii Cortex	root - bark of Chinese Wolfberry	清虚热药	Asthenic Heat Dispelling Drugs	0.0410077	0.062858	1	By_ingredient	0.1622
SMHB00104	地锦草	Dijincao	Euphorbiae Humifusae Herba	all - grass of Humifuse Euphorbia	清热解毒药	Antipyretic Detoxicate Drugs	4.504e-04	0.0041248	1	By_ingredient	0.3238
SMHB00105	地榆	Diyu	Sanguisorbae Radix	Root of Garden Burnet	凉血止血药	Blood-Cooling Hemostatic Medicinal	0.0429212	0.0649773	1	By_ingredient	0.1544
SMHB00107	丁香	Dingxiang	Caryophylli Flos	Clove	温里药	Warming Interior Drugs	0.00324694	0.0153926	1	By_ingredient	0.5111

Herb id	Chinese name	Pinyin name	Latin name	English name	Class in Chinese	Class in English	P_value	FDR(BH)	FDR(Bonferroni)	Relationship	Inferred evidence score
SMHB00108	冬虫夏草	Dongchongxiacao	Cordyceps	Chinese Caterpillar Fungus	补阳药	Yang Reinforcing Drugs	0.0792293	0.10324	1	By_ingredient	0.1674
SMHB00111	冬葵果	Dongkuiguo	Malvae Fructus	Cluster Mallow Fruit	拔毒化腐生肌药	External Medicinal (Draw Out Toxin, Resolve Putridity)	0.00201563	0.0117624	1	By_ingredient	0.3294
SMHB00112	豆蔻	Doukou	Amomi Fructus Rotundus	Round Cardamon Fruit	化湿药	Dampness Removing Drugs	0.0216347	0.0635209	1	By_ingredient	0.3208
SMHB00113	独活	Duhuo	Angelicae Pubescentis Radix	Doubleteeth Pubescent Angelica Root	祛风湿散寒药	Wind-Dampnessdispelling And Colddispersing Medicinal	0.0674777	0.0910188	1	By_ingredient	0.1574
SMHB00114	杜仲	Duzhong	Eucommiae Cortex	Eucommia bark	补阳药	Yang Reinforcing Drugs	0.0041954	0.0188592	1	By_ingredient	0.4657
SMHB00115	杜仲叶	Duzhongye	Eucommiae Folium	Eucommiae Folium	拔毒化腐生肌药	External Medicinal (Draw Out Toxin, Resolve Putridity)	0.00418406	0.0173669	1	By_ingredient	0.332
SMHB00118	鹅不食草	Ebushicao	Centipedae Herba	Small Centipeda Herb	辛温解表药	Pungent-Warm Exterior-Releasing Medicinal	0.00104012	0.0152622	1	By_ingredient	0.4756
SMHB00119	儿茶	Ercha	Catechu	Cutch, Black Catechu	活血祛瘀药	Blood Activating Stasis Removing Drugs	0.0307428	0.0517439	1	By_ingredient	0.1416
SMHB00120	番泻叶	Fanxieye	Sennae Folium	Sen leaf	攻下药	Offensive Purgative Medicinal	0.0511723	0.0738216	1	By_ingredient	0.1641
SMHB00121	翻白草	Fanbaicao	Potentillae Discoloris Herba	all-grass of discolor cinquefoil	清热解毒药	Antipyretic Detoxicate Drugs	0.0236269	0.0436071	1	By_ingredient	0.167
SMHB00122	防风	Fangfeng	Saposhnikoviae Radix	root of Divaricate Saposhnikovia	辛温解表药	Pungent-Warm Exterior-Releasing Medicinal	0.0382073	0.0798203	1	By_ingredient	0.3383
SMHB00123	防己	Fangji	Stephaniae Tetrandrae Radix	Root of Fourstamen Stephania	祛风湿清热药	Wind-Dampnessdispelling And Heatclearing Medicinal	0.0543282	0.0772065	1	By_ingredient	0.1608
SMHB00125	凤尾草	Fengweicao	Herba Pteridis Multifidae	all - grass of Chinese Brake	清热解毒药	Antipyretic Detoxicate Drugs	9.294e-04	0.00688769	1	By_ingredient	0.3114
SMHB00126	凤仙透骨草	Fengxiantougucao	Herba Speranskiae Tuberculatae	all-grass of Tubercultae speranskia	祛风湿散寒药	Wind-Dampnessdispelling And Colddispersing Medicinal	2.120e-04	0.00246516	1	By_ingredient	0.3089
SMHB00127	凤眼草	Fengyancao	None	Ailanthi Altissimae Fructus	清热解毒药	Antipyretic Detoxicate Drugs	0.0203765	0.0396878	1	By_ingredient	0.1459
SMHB00128	佛手	Foshou	Citri Sarcodactylis Fructus	Finger Citron	理气药	Qi Regulating Drugs	0.0536978	0.0764999	1	By_ingredient	0.1648
SMHB00129	茯苓	Fuling	Poria	Indian Bread	利水渗湿药	Diuretic Dampness Excreting Drugs	0.00182534	0.0110243	1	By_ingredient	0.5862
SMHB00130	浮萍	Fuping	Spirodelae Herba	all-grass of Common Ducksmeat	辛凉解表药	Pungent Cool Diaphoretics	0.0410077	0.062858	1	By_ingredient	0.1634
SMHB00131	附子	Fuzi	Aconiti Lateralis Radix Praeparata	Common Monkshood Daughter Root	温里药	Warming Interior Drugs	0.0221006	0.064009	1	By_ingredient	0.3912
SMHB00132	覆盆子	Fupenzi	Rubi Fructus	Rubi Fructus	收涩药	Astringent Medicinal	0.0262269	0.0680699	1	By_ingredient	0.304
SMHB00133	甘草	Gancao	Glycyrrhizae Radix Et Rhizoma	Root of Ural Licorice	补气药	Qi Reinforcing Drugs	0.172346	0.199167	1	By_ingredient	0.1578
SMHB00134	甘松	Gansong	Nardostachyos Radix Et Rhizoma	rdostachys Root	理气药	Qi Regulating Drugs	0.045467	0.0677509	1	By_ingredient	0.1624
SMHB00135	甘遂	Gansui	Kansui Radix	root of Gansui	逐水药	Drastic Purgatives	5.492e-04	0.00472388	1	By_ingredient	0.4247
SMHB00136	干姜	Ganjiang	Zingiberis Rhizoma	Dried Ginger	温里药	Warming Interior Drugs	0.0104049	0.0272792	1	By_ingredient	0.2925
SMHB00137	高良姜	Gaoliangjiang	Alpiniae Officinarum Rhizoma	Alpiniae Officirum Rhizome	温里药	Warming Interior Drugs	0.00150519	0.0163592	1	By_ingredient	0.4992
SMHB00138	藁本	Gaoben	Ligustici Rhizoma Et Radix	Ligustici Rhizoma Et Radix	辛温解表药	Pungent-Warm Exterior-Releasing Medicinal	0.00845269	0.0444644	1	By_ingredient	0.3408
SMHB00139	葛花	Gehua	Flos Puerariae Lobatae	flower bud of lobed kudzuvine	辛凉解表药	Pungent Cool Diaphoretics	3.434e-04	0.00343665	1	By_ingredient	0.4582
SMHB00140	功劳木	Gonglaomu	Mahoniae Caulis	Stem of Leatherleaf Mahonia	清热解毒药	Antipyretic Detoxicate Drugs	0.00814848	0.0438602	1	By_ingredient	0.3092
SMHB00141	钩藤	Gouteng	Uncariae Ramulus Cumuncis	Gambir Plant	平肝熄风药	Liver-Pacifying Wind-Extinguishing Medicinal	0.0189283	0.0601914	1	By_ingredient	0.3284
SMHB00142	枸骨叶	Gouguye	Ilicis Cornutae Folium	Folium Ilicis Cornutae	清虚热药	Asthenic Heat Dispelling Drugs	0.0149651	0.0553614	1	By_ingredient	0.2994
SMHB00143	枸杞子	Gouqizi	Lycii Fructus	fruit of Chinese Wolfberry	补阴药	Yin-Tonifying Medicinal	0.00801196	0.0295348	1	By_ingredient	0.4567
SMHB00144	谷精草	Gujingcao	Eriocauli Flos	Pipewort Flower	清热泻火药	Fire Purging Drugs	3.283e-04	0.00333635	1	By_ingredient	0.3082
SMHB00147	瓜蒌	Gualou	Trichosanthis Fructus	Trichosanthes Kirilowii Maxim	化痰药	Phlegresolving Medicine	0.056846	0.0797696	1	By_ingredient	0.1657
SMHB00148	关黄柏	Guanhuangbo	Phellodendri Amurensis Cortex	Phellodendri Amurensis Cortex	清热燥湿药	Heat-Clearing And Dampnessdrying Medicinal	0.0680997	0.0917392	1	By_ingredient	0.1513
SMHB00149	贯叶金丝桃	Guanyejinsitao	Hyperici Perforati Herba	Herbahypericiperforati	收敛止血药	Astringent Hemostatic Medicinal	0.0612372	0.0844632	1	By_ingredient	0.1317
SMHB00150	绵马贯众	Mianmaguanzhong	Dryopteridis Crassirhizomatis Rhizoma	root of Chinese Angelica	清热解毒药	Antipyretic Detoxicate Drugs	0.0448312	0.0670828	1	By_ingredient	0.1852
SMHB00151	广藿香	Guanghuoxiang	Pogostemonis Herba	Pogostemon Cablin (Blanco) Benth.	化湿药	Dampness Removing Drugs	0.00300666	0.0148459	1	By_ingredient	0.3132
SMHB00152	广枣	Guangzao	Choerospondiatis Fructus	fruit of Axillary choerospondias	活血祛瘀药	Blood Activating Stasis Removing Drugs	5.26E-05	9.365e-04	1	By_ingredient	0.4764
SMHB00153	鬼箭羽	Guijianyu	Ramulus Euonymi	twig of Winged Euonymus	活血祛瘀药	Blood Activating Stasis Removing Drugs	0.00272996	0.0140841	1	By_ingredient	0.3382
SMHB00154	鬼针草	Guizhencao	Herba Bidentis	all-grass of Spanishneedles	清热解毒药	Antipyretic Detoxicate Drugs	9.564e-04	0.00702214	1	By_ingredient	0.2977
SMHB00155	桂枝	Guizhi	Cinnamomi Ramulus	Cassia Twig	辛温解表药	Pungent-Warm Exterior-Releasing Medicinal	0.151489	0.177614	1	By_ingredient	0.2957
SMHB00158	海桐皮	Haitongpi	Cortex Erythrie	bark of Oriental Variegated Coralbean	祛风湿清热药	Wind-Dampnessdispelling And Heatclearing Medicinal	0.0352461	0.0566291	1	By_ingredient	0.1734
SMHB00159	海藻	Haizao	Sargassum	seaweed	化痰药	Phlegresolving Medicine	0.0268674	0.0473109	1	By_ingredient	0.1519
SMHB00160	诃子	Hezi	Chebulae Fructus	Medicine Termilia Fruit	收涩药	Astringent Medicinal	0.0358879	0.057326	1	By_ingredient	0.174
SMHB00161	荷叶	Heye	Nelumbinis Folium	Lotus leaf	清热解毒药	Antipyretic Detoxicate Drugs	5.54E-05	9.724e-04	1	By_ingredient	0.5442
SMHB00162	核桃仁	Hetaoren	Juglandis Semen	English Walnut Seed	补阳药	Yang Reinforcing Drugs	6.063e-04	0.01141	1	By_ingredient	0.5135
SMHB00164	黑豆	Heidou	Sojae Semen Nigrum	Black bean	补阴药	Yin-Tonifying Medicinal	0.109504	0.134827	1	By_ingredient	0.1711
SMHB00167	红参	Hongshen	Ginseng Radix Et Rhizoma Rubra	Red Ginseng	补气药	Qi Reinforcing Drugs	0.0680997	0.0917392	1	By_ingredient	0.3974
SMHB00168	红花	Honghua	Carthami Flos	Safflower	活血祛瘀药	Blood Activating Stasis Removing Drugs	0.00686794	0.0223202	1	By_ingredient	0.2858
SMHB00169	红芪	Hongqi	Hedysari Radix	Hedysari Radix	补气药	Qi Reinforcing Drugs	0.0981097	0.122933	1	By_ingredient	0.1429
SMHB00171	厚朴	Houpo	Magnoliae Officinalis Cortex	bark of Officil magnolia	化湿药	Dampness Removing Drugs	0.00603491	0.0390382	1	By_ingredient	0.5775
SMHB00173	胡椒	Hujiao	Piperis Fructus	Pepper Fruit	温里药	Warming Interior Drugs	0.0786142	0.102618	1	By_ingredient	0.1552
SMHB00174	胡芦巴	Huluba	Trigonellae Semen	Trigonellae Semen	补阳药	Yang Reinforcing Drugs	0.0724425	0.0962893	1	By_ingredient	0.1662
SMHB00175	槲寄生	Hujisheng	Visci Herba	Colored Mistletoe Herb	祛风湿筋骨药	Wind-Damp-Dispelling And Sinew-Bone-Strengthening Medicinal	0.0612372	0.0844632	1	By_ingredient	0.1591
SMHB00176	虎耳草	Huercao	Herba Saxifragae	stem or leaf of Fung Waxplant	清热解毒药	Antipyretic Detoxicate Drugs	4.504e-04	0.0041248	1	By_ingredient	0.283
SMHB00177	虎杖	Huzhang	Polygoni Cuspidati Rhizoma Et Radix	rhizome of Gaint Knotweed	利水渗湿药	Diuretic Dampness Excreting Drugs	0.0193687	0.0603893	1	By_ingredient	0.3039
SMHB00178	花椒	Huajiao	Zanthoxyli Pericarpium	Pricklyash Peel	温里药	Warming Interior Drugs	0.00209428	0.0120211	1	By_ingredient	0.2907
SMHB00179	花生衣	Huashengyi	Testa Arachidis Hypogaeae	Semino-dermis Arachidis	收敛止血药	Astringent Hemostatic Medicinal	0.042209	0.0837456	1	By_ingredient	0.1386
SMHB00181	化橘红	Huajuhong	Citri Grandis Exocarpium	Pummelo Peel	理气药	Qi Regulating Drugs	0.0656096	0.0889693	1	By_ingredient	0.1598
SMHB00182	槐花	Huaihua	Sophorae Flos	flower bud of Japanese pagodatree	凉血止血药	Blood-Cooling Hemostatic Medicinal	0.1101	0.135449	1	By_ingredient	0.1478
SMHB00183	槐角	Huaijiao	Sophorae Fructus	pod of Japanese pagodatree	凉血止血药	Blood-Cooling Hemostatic Medicinal	0.0307428	0.0517439	1	By_ingredient	0.1417
SMHB00184	黄柏	Huangbo	Phellodendri Chinensis Cortex	Phellodendron bark	清热燥湿药	Heat-Clearing And Dampnessdrying Medicinal	0.00469442	0.0184709	1	By_ingredient	0.298
SMHB00186	黄连	Huanglian	Coptidis Rhizoma	rhizome of Chinese Goldthread	清热燥湿药	Heat-Clearing And Dampnessdrying Medicinal	7.755e-04	0.00605911	1	By_ingredient	0.3502
SMHB00187	黄芪	Huangqi	Astragali Radix	root of Membranous Milkvetch	补气药	Qi Reinforcing Drugs	3.016e-04	0.00709006	1	By_ingredient	0.6872
SMHB00188	黄芩	Huangqin	Scutellariae Radix	root of Baikal skullcap	清热燥湿药	Heat-Clearing And Dampnessdrying Medicinal	0.102323	0.127294	1	By_ingredient	0.3483
SMHB00189	黄药子	Huangyaozi	Rhizoma Dioscoreae Bulbiferae	rhizome of Airpotato Yam	化痰药	Phlegresolving Medicine	1.38E-05	3.555e-04	1	By_ingredient	0.5326
SMHB00190	火麻仁	Huomaren	Cannabis Fructus	Hemp Seed	润下药	Laxatives	0.12312	0.148745	1	By_ingredient	0.1757
SMHB00192	鸡冠花	Jiguanhua	Celosiae Cristatae Flos	Cockscomb flower	收敛止血药	Astringent Hemostatic Medicinal	0.0138457	0.0319753	1	By_ingredient	0.1635
SMHB00193	鸡屎藤	Jishiteng	None	Paederiae Scandentis Herba	消食药	Digestants	0.00391251	0.0167799	1	By_ingredient	0.2686
SMHB00195	积雪草	Jixuecao	Centellae Herba	Asiatic Pennywort Herb	清热解毒药	Antipyretic Detoxicate Drugs	0.00101147	0.007304	1	By_ingredient	0.3086
SMHB00196	急性子	Jixingzi	Impatientis Semen	seed of Garden Balsam	活血祛瘀药	Blood Activating Stasis Removing Drugs	0.0365292	0.0580469	1	By_ingredient	0.1663
SMHB00198	姜黄	Jianghuang	Curcumae Longae Rhizoma	rhizome of Common Turmeric	活血祛瘀药	Blood Activating Stasis Removing Drugs	0.0176334	0.0585289	1	By_ingredient	0.3549
SMHB00199	降香	Jiangxiang	Dalbergiae Odoriferae Lignum	Odoriferous Rosewood	理气药	Qi Regulating Drugs	0.0240057	0.0656858	1	By_ingredient	0.2913
SMHB00200	绞股蓝	Jiaoguolan	None	Gynostemmae Pentaphylli Herba	补气药	Qi Reinforcing Drugs	3.387e-04	0.00340039	1	By_ingredient	0.552
SMHB00201	芥子	Jiezi	Sinapis Semen	Mustard Seed	化痰药	Phlegresolving Medicine	0.108908	0.134201	1	By_ingredient	0.1774
SMHB00202	金沸草	Jinfeicao	Inulae Herba	All - grass of Japanese Inula	化痰药	Phlegresolving Medicine	0.00421082	0.0339191	1	By_ingredient	0.2934
SMHB00204	金莲花	Jinlianhua	Flos Trollii Chinensis	flower of Chinese Globeflower	清热解毒药	Antipyretic Detoxicate Drugs	9.028e-04	0.00677462	1	By_ingredient	0.3171
SMHB00205	金钱草	Jinqiancao	Lysimachiae Herba	Christi Loosestrife	利水渗湿药	Diuretic Dampness Excreting Drugs	0.0624884	0.0856835	1	By_ingredient	0.1461
SMHB00206	金荞麦	Jinqiaomai	Fagopyri Dibotryis Rhizoma	Wild Buckwheat Rhizome	清热解毒药	Antipyretic Detoxicate Drugs	0.00118569	0.00820864	1	By_ingredient	0.3109

Herb id	Chinese name	Pinyin name	Latin name	English name	Class in Chinese	Class in English	P_value	FDR(BH)	FDR(Bonferroni)	Relationship	Inferred evidence score
SMHB00207	金银花	Jinyinhua	Lonicerae Japonicae Flos	Honeysuckle Flower	清热解毒药	Antipyretic Detoxicate Drugs	0.00109245	0.0077364	1	By_ingredient	0.4644
SMHB00208	金樱子	Jinyingzi	Rosae Laevigatae Fructus	fruit of Cherokee Rose	收涩药	Astringent Medicinal	0.0010969	0.00775987	1	By_ingredient	0.2854
SMHB00212	荆芥	Jingjie	Schizonepetae Herba	all-grass of Fineleaf Schizonepeta	辛温解表药	Pungent-Warm Exterior-Releasing Medicinal	0.008172	0.0240643	1	By_ingredient	0.3162
SMHB00213	荆芥穗	Jingjiesui	Schizonepetae Spica	Schizonepetae Spica	辛温解表药	Pungent-Warm Exterior-Releasing Medicinal	0.0461025	0.0684049	1	By_ingredient	0.1585
SMHB00214	景天三七	Jintiansanqi	None	Sedum Aizoon L.	化瘀止血药	Stasis-Resolving Hemostatic Medicinal	0.024737	0.0666468	1	By_ingredient	0.4129
SMHB00215	韭菜子	Jiucaizi	Allii Tuberosi Semen	Tuber Onion Seed	补阳药	Yang Reinforcing Drugs	0.0339614	0.0552195	1	By_ingredient	0.1558
SMHB00216	桔梗	Jiegeng	Platycodonis Radix	Platycodon Root	化痰药	Phlegresolving Medicine	0.0773829	0.101427	1	By_ingredient	0.1676
SMHB00217	菊花	Juhua	Chrysanthemi Flos	Chrysanthemum Flower	辛凉解表药	Pungent Cool Diaphoretics	0.0249039	0.0450233	1	By_ingredient	0.307
SMHB00218	菊苣	Juju	Cichorii Herba Cichorii Radix	Chicory Herb	拔毒化腐生肌药	External Medicinal (Draw Out Toxin, Resolve Putridity)	5.24E-05	0.00205065	1	By_ingredient	0.665
SMHB00219	橘核	Juhe	Citri Reticulatae Semen	Tangerine Seed	理气药	Qi Regulating Drugs	0.0339614	0.0552195	1	By_ingredient	0.1491
SMHB00222	橘叶	Juye	Folium Citri Tangerie	tangerine leaf	理气药	Qi Regulating Drugs	0.0352461	0.0566291	1	By_ingredient	0.1746
SMHB00223	卷柏	Juanbai	Selaginellae Herba	aal-grass of Tamariskoid spikemoss	化瘀止血药	Stasis-Resolving Hemostatic Medicinal	0.0403691	0.0621518	1	By_ingredient	0.1489
SMHB00225	苦参	Kushen	Sophorae Flavescentis Radix	root of Lightyellow sophora	清热燥湿药	Heat-Clearing And Dampnessdrying Medicinal	0.0730614	0.0969719	1	By_ingredient	0.162
SMHB00227	苦丁茶	Kudingcha	None	Ilicis Cornutae Folium Immayuri	清热泻火药	Fire Purging Drugs	0.101722	0.126699	1	By_ingredient	0.1571
SMHB00229	苦楝子	Kulianzi	None	Meliae Azedarach Fructus	驱虫药	Antiparasitic Drugs	0.0448312	0.0670828	1	By_ingredient	0.1493
SMHB00231	款冬花	Kuandonghua	Farfarae Flos	flower bud of common coltsfoot	止咳平喘药	Antitussive Antiasthmetics	1.438e-04	0.00188178	1	By_ingredient	0.4851
SMHB00232	昆布	Kunbu	Laminariae Thallus Eckloniae Thallus	Kelp or Tangle	化痰药	Phlegresolving Medicine	0.0077017	0.0433314	1	By_ingredient	0.3487
SMHB00233	辣椒	Lajiao	Capsici Fructus	Hot pepper	拔毒化腐生肌药	External Medicinal (Draw Out Toxin, Resolve Putridity)	0.0111742	0.0282204	1	By_ingredient	0.3535
SMHB00234	莱菔子	Laifuzi	Raphani Semen	Radish Seed	消食药	Digestants	0.0461025	0.0684049	1	By_ingredient	0.1594
SMHB00235	老鹳草	Laoguancao	Erodii Herba Geranii Herba	All - grass of Common Heron's bill	祛风湿散寒药	Wind-Dampnessdispelling And Colddispersing Medicinal	0.0326752	0.0536532	1	By_ingredient	0.1431
SMHB00237	雷公藤	Leigongteng	Radix Tripterygii Wilfordii	root of common htreewingnut	祛风湿散寒药	Wind-Dampnessdispelling And Colddispersing Medicinal	0.113075	0.138555	1	By_ingredient	0.1724
SMHB00239	荔枝核	Lizhihe	Litchi Semen	Litchi seed	理气药	Qi Regulating Drugs	0.00205477	0.0118973	1	By_ingredient	0.2748
SMHB00240	连钱草	Lianqiancao	Glechomae Herba	Glechomae Herba	利水渗湿药	Diuretic Dampness Excreting Drugs	0.00182113	0.0181614	1	By_ingredient	0.5136
SMHB00241	连翘	Lianqiao	Forsythiae Fructus	Weeping Forsythia Capsule	清热解毒药	Antipyretic Detoxicate Drugs	0.00693764	0.0224754	1	By_ingredient	0.3358
SMHB00242	莲房	Lianfang	Nelumbinis Receptaculum	Lotus Receptacle	收敛止血药	Astringent Hemostatic Medicinal	0.00661542	0.0217182	1	By_ingredient	0.1683
SMHB00243	莲须	Lianxu	Nelumbinis Stamen	Lotus stamen	收涩药	Astringent Medicinal	0.045467	0.0677509	1	By_ingredient	0.148
SMHB00244	莲子心	Lianzixin	Nelumbinis Plumula	Lotus Plumule	收涩药	Astringent Medicinal	0.0294526	0.0503154	1	By_ingredient	0.1561
SMHB00250	零陵香	Linglingxiang	None	Lysimachiae Foenigraeci Herba	辛温解表药	Pungent-Warm Exterior-Releasing Medicinal	0.00736266	0.0229399	1	By_ingredient	0.2879
SMHB00251	龙胆	Longdan	Gentianae Radix Et Rhizoma	Gentiae Radix Et Rhozima	清热燥湿药	Heat-Clearing And Dampnessdrying Medicinal	0.0435582	0.0656505	1	By_ingredient	0.1654
SMHB00252	龙葵	Longkui	None	Solanum Nigrum Linn.	清热解毒药	Antipyretic Detoxicate Drugs	0.0100491	0.0474436	1	By_ingredient	0.3101
SMHB00253	龙脷叶	Longliye	Sauropi Folium	Aauropi Folium	拔毒化腐生肌药	External Medicinal (Draw Out Toxin, Resolve Putridity)	0.0643623	0.0876099	1	By_ingredient	0.1577
SMHB00254	漏芦	Loulu	Rhapontici Radix	Uniflower Swisscentaury Root	清热解毒药	Antipyretic Detoxicate Drugs	0.0448312	0.0670828	1	By_ingredient	0.1597
SMHB00255	芦根	Lugen	Phragmitis Rhizoma	Reed rhizome	清热泻火药	Fire Purging Drugs	0.0712036	0.0949209	1	By_ingredient	0.1432
SMHB00256	芦荟	Luhui	Aloe	Aloe	攻下药	Offensive Purgative Medicinal	0.0011	0.0117624	1	By_ingredient	0.4713
SMHB00257	鹿衔草	Luxiancao	Pyrolae Herba	all-grass Chinese pyrola	祛风湿散寒药	Wind-Dampnessdispelling And Colddispersing Medicinal	0.0422838	0.0642556	1	By_ingredient	0.141
SMHB00260	罗汉果	Luohanguo	Siraitiae Fructus	Grosvenor Momordica Fruit	化痰药	Phlegresolving Medicine	0.0277525	0.0699559	1	By_ingredient	0.3258
SMHB00261	络石藤	Luoshiteng	Trachelospermi Caulis Et Folium	stem of Chinese starjasmine	祛风湿清热药	Wind-Dampnessdispelling And Heatclearing Medicinal	7.272e-04	0.00578039	1	By_ingredient	0.3787
SMHB00263	葎草	Lvcao	Herba Humuli Scandentis	all-grass of japanese hop	清热解毒药	Antipyretic Detoxicate Drugs	0.0656096	0.0889693	1	By_ingredient	0.162
SMHB00264	麻黄	Mahuang	Ephedrae Herba	Ephedra	辛温解表药	Pungent-Warm Exterior-Releasing Medicinal	0.00385802	0.0328001	1	By_ingredient	0.7074
SMHB00265	麻黄根	Mahuanggen	Ephedrae Radix Et Rhizoma	Ephedra Root	收涩药	Astringent Medicinal	0.045467	0.0677509	1	By_ingredient	0.1436
SMHB00266	马鞭草	Mabiancao	Verbenae Herba	European Verbe Herb	活血祛瘀药	Blood Activating Stasis Removing Drugs	1.849e-04	0.00294881	1	By_ingredient	0.6123
SMHB00267	马勃	Mabo	Lasiosphaera Calvatia	Puff-ball	清热解毒药	Antipyretic Detoxicate Drugs	0.0229776	0.0428653	1	By_ingredient	0.1638
SMHB00268	马齿苋	Machixian	Portulacae Herba	all-grass of Purslane	清热解毒药	Antipyretic Detoxicate Drugs	0.00115572	0.0080656	1	By_ingredient	0.295
SMHB00271	马尾莲	Maweilian	Radix Et Rhizoma Thalictri	root and rhizome of Manyleaf Meadowrue	清热燥湿药	Heat-Clearing And Dampnessdrying Medicinal	0.101722	0.126699	1	By_ingredient	0.1478
SMHB00272	麦芽	Maiya	Hordei Fructus Germinatus	Germited Barley	消食药	Digestants	5.523e-04	0.0106732	1	By_ingredient	0.5222
SMHB00273	满山红	Manshanhong	Rhododendri Daurici Folium	Dahurian Rhododendron Leaf	止咳平喘药	Antitussive Antiasthmetics	0.00599159	0.0207263	1	By_ingredient	0.2986
SMHB00274	蔓荆子	Manjingzi	Viticis Fructus	Shrub Chastetree Fruit	辛凉解表药	Pungent Cool Diaphoretics	0.0387938	0.0801935	1	By_ingredient	0.3379
SMHB00275	猫眼草	Maoyancao	None	Lunulatae Herba	化痰药	Phlegresolving Medicine	0.0410077	0.062858	1	By_ingredient	0.151
SMHB00276	猫爪草	Maozhaocao	Ranunculi Ternati Radix	Catclaw Buttercup Root	清热解毒药	Antipyretic Detoxicate Drugs	0.0643623	0.0876099	1	By_ingredient	0.1693
SMHB00277	没药	Moyao	Myrrha	Myrrh	活血祛瘀药	Blood Activating Stasis Removing Drugs	4.504e-04	0.0041248	1	By_ingredient	0.8792
SMHB00278	玫瑰花	Meiguihua	Rosae Rugosae Flos	Rose Flower	理气药	Qi Regulating Drugs	0.00380629	0.0166477	1	By_ingredient	0.37
SMHB00280	猕猴桃根	Mihoutaogen	None	Actinidia Chinensis Planch	清热解毒药	Antipyretic Detoxicate Drugs	6.576e-04	0.00536172	1	By_ingredient	0.3166
SMHB00282	绵萆薢	Mianbixie	Dioscoreae Spongiosae Rhizoma	Dioscoreae Septemlo Bae Rhizoma	利水渗湿药	Diuretic Dampness Excreting Drugs	0.0119472	0.0501843	1	By_ingredient	0.313
SMHB00283	墨旱莲	Mohanlian	Ecliptae Herba	all-grass of Yerbadetajo	补阴药	Yin-Tonifying Medicinal	0.00590013	0.0388881	1	By_ingredient	0.3162
SMHB00284	牡丹皮	Mudanpi	Moutan Cortex	Tree Peony Bark	清热凉血药	Heat Clearing Blood Cooling Drugs	0.0178465	0.0585289	1	By_ingredient	0.3344
SMHB00286	牡荆子	Mujingzi	None	Viticis Canbifoliae Fructus	止咳平喘药	Antitussive Antiasthmetics	0.00679856	0.0221473	1	By_ingredient	0.509
SMHB00287	木鳖子	Mubiezi	Momordicae Semen	Momordicae Semen	拔毒化腐生肌药	External Medicinal (Draw Out Toxin, Resolve Putridity)	0.0268674	0.0473109	1	By_ingredient	0.1596
SMHB00288	木瓜	Mugua	Chaenomelis Fructus	fruit of Common Floweringquince	祛风湿散寒药	Wind-Dampnessdispelling And Colddispersing Medicinal	0.0414757	0.0804332	1	By_ingredient	0.289
SMHB00289	木蝴蝶	Muhudie	Oroxyli Semen	Indian Trumpetflower Seed	清热解毒药	Antipyretic Detoxicate Drugs	5.655e-04	0.0108706	1	By_ingredient	0.4406
SMHB00290	木棉花	Mumianhua	Gossampini Flos	Flower of Common Bombax	拔毒化腐生肌药	External Medicinal (Draw Out Toxin, Resolve Putridity)	0.00729102	0.0228635	1	By_ingredient	0.2954
SMHB00293	木贼	Muzei	Equiseti Hiemalis Herba	Common Scouring Rush Herb	辛凉解表药	Pungent Cool Diaphoretics	0.0624884	0.0856835	1	By_ingredient	0.1461
SMHB00294	墓头回	Mutouhui	None	Et Radix Patriniae Rhizoma	清热解毒药	Antipyretic Detoxicate Drugs	0.00160901	0.010122	1	By_ingredient	0.2723
SMHB00295	南瓜子	nguazi	Semen Cucurbitae Moschatae	Pumpkin seed and husks	驱虫药	Antiparasitic Drugs	3.124e-04	0.00323282	1	By_ingredient	0.3612
SMHB00298	南蛇藤果	nshetengguo	None	Celastri Orbiculati Fructus	安神药	Tranguilizing Medicinal	0.00205477	0.0118973	1	By_ingredient	0.2887
SMHB00300	闹羊花	Naoyanghua	Rhododendri Mollis Flos	Yellow Azalea Flower	祛风湿散寒药	Wind-Dampnessdispelling And Colddispersing Medicinal	0.0562171	0.0791687	1	By_ingredient	0.1554
SMHB00301	牛蒡子	Niubangzi	Arctil Fructus	achene of Great Burdock	辛凉解表药	Pungent Cool Diaphoretics	0.111886	0.137281	1	By_ingredient	0.1496
SMHB00302	牛大力	Niudali	None	Millettiae Speciosae Radix	止咳平喘药	Antitussive Antiasthmetics	0.0668554	0.0903192	1	By_ingredient	0.1592
SMHB00304	牛膝	Niuxi	Achyranthis Bidentatae Radix	root of Twotooth Achyranthes	活血祛瘀药	Blood Activating Stasis Removing Drugs	3.116e-04	0.00322632	1	By_ingredient	0.5028
SMHB00306	女贞子	Nüzhenzi	Ligustri Lucidi Fructus	fruit of Glossy privet	补阴药	Yin-Tonifying Medicinal	0.00142579	0.00933143	1	By_ingredient	0.5246
SMHB00307	藕节	Oujie	Nelumbinis Rhizomatis Nodus	node of lotus rhizome	收敛止血药	Astringent Hemostatic Medicinal	0.0219646	0.063849	1	By_ingredient	0.1792
SMHB00308	胖大海	Pangdahai	Sterculiae Lychnophorae Semen	Boat-fruited Sterculia Seed	化痰药	Phlegresolving Medicine	0.00101147	0.007304	1	By_ingredient	0.3451
SMHB00310	枇杷叶	Pipaye	Eriobotryae Folium	Loquat Leaf	止咳平喘药	Antitussive Antiasthmetics	0.0113229	0.0359025	1	By_ingredient	0.4836
SMHB00311	平贝母	Pingbeimu	Fritillariae Ussuriensis Bulbus	Ussuri Fritillary Bulb	化痰药	Phlegresolving Medicine	0.0339614	0.0552195	1	By_ingredient	0.1719
SMHB00312	蒲黄	Puhuang	Typhae Pollen	pollen of longbract cattail	化瘀止血药	Stasis-Resolving Hemostatic Medicinal	0.0390907	0.0608393	1	By_ingredient	0.1518
SMHB00313	荠菜	Jicai	Herba Capsellae	all-grass of Shepherdspurse	利水渗湿药	Diuretic Dampness Excreting Drugs	0.0010969	0.00775987	1	By_ingredient	0.3026
SMHB00314	千斤拔	Qianjinba	Radix Moghaniae Philippinensis	root of Philippine Flemingia	祛风湿筋骨药	Wind-Damp-Dispelling And Sinew-Bone-Strengthening Medicinal	0.038451	0.0601681	1	By_ingredient	0.1488
SMHB00315	千金子	Qianjinzi	Euphorbiae Semen	Caper Euphorbia Seed	通水药	Drastic Purgatives	0.0365292	0.0580469	1	By_ingredient	0.1658
SMHB00316	千年健	Qiannianjian	Homalomenae Rhizoma	rhizome of Obscured Homalome	祛风湿筋骨药	Wind-Damp-Dispelling And Sinew-Bone-Strengthening Medicinal	0.0486405	0.0710131	1	By_ingredient	0.1502
SMHB00318	牵牛子	Qianniuzi	Pharbitidis Semen	Pharbitis seed	通水药	Drastic Purgatives	0.00829995	0.0441675	1	By_ingredient	0.323

Herb id	Chinese name	Pinyin name	Latin name	English name	Class in Chinese	Class in English	P_value	FDR(BH)	FDR(Bonferroni)	Relationship	Inferred evidence score
SMHB00319	前胡	Qianhu	Peucedani Radix	root of Grand Hogfennel	化痰药	Phlegresolving Medicine	1.412e-04	0.00185931	1	By_ingredient	0.506
SMHB00320	芡实	Qianshi	Euryales Semen	seed of Gordon Euryale	收涩药	Astringent Medicinal	0.038451	0.0601681	1	By_ingredient	0.15
SMHB00321	茜草	Qiancao	Rubiae Radix Et Rhizoma	root of Tali Madder	化瘀止血药	Stasis-Resolving Hemostatic Medicinal	0.00112612	0.00791278	1	By_ingredient	0.3719
SMHB00322	羌活	Qianghuo	Notopterygii Rhizoma Et Radix	Root and rhizome of Incised Notoptergium	辛温解表药	Pungent-Warm Exterior-Releasing Medicinal	0.0161961	0.0346355	1	By_ingredient	0.4989
SMHB00328	青果	Qingguo	Canarii Fructus	Carii Fructus	清热解毒药	Antipyretic Detoxicate Drugs	9.837e-04	0.00715392	1	By_ingredient	0.315
SMHB00329	青蒿	Qinghao	Artemisiae Annuae Herba	Sweet Wormwood Herb	清虚热药	Asthenic Heat Dispelling Drugs	0.0301068	0.07198	1	By_ingredient	0.3346
SMHB00331	青葙子	Qingxiangzi	Celosiae Semen	Celosiae Semen	清热泻火药	Fire Purging Drugs	0.0190735	0.0382423	1	By_ingredient	0.1673
SMHB00333	苘麻子	Qingmazi	Abutili Semen	Chingma Abutilon Seed	利水渗湿药	Diuretic Dampness Excreting Drugs	0.00990795	0.0266273	1	By_ingredient	0.1909
SMHB00334	瞿麦	Qumai	Dianthi Herba	all-grass of Lilac Pink	利水渗湿药	Diuretic Dampness Excreting Drugs	0.0480066	0.0703351	1	By_ingredient	0.1563
SMHB00335	拳参	Quanshen	Bistortae Rhizoma	rhizome of Bistort	清热解毒药	Antipyretic Detoxicate Drugs	0.0313873	0.0523102	1	By_ingredient	0.1471
SMHB00336	人参	Renshen	Ginseng Radix Et Rhizoma	Ginseng	补气药	Qi Reinforcing Drugs	0.0218324	0.0636904	1	By_ingredient	0.7197
SMHB00337	人参叶	Renshenye	Ginseng Folium	Ginseng Leaf	补气药	Qi Reinforcing Drugs	0.00359803	0.0161857	1	By_ingredient	0.5436
SMHB00338	肉苁蓉	Roucongrong	Cistanches Herba	Desertliving Cistanche	补阳药	Yang Reinforcing Drugs	0.00344495	0.0315771	1	By_ingredient	0.5542
SMHB00340	肉桂	Rougui	Cinnamomi Cortex	Cassia Bark	温里药	Warming Interior Drugs	0.0356117	0.0770765	1	By_ingredient	0.2806
SMHB00341	乳香	Ruxiang	Olibanum	Frankincense	活血祛瘀药	Blood Activating Stasis Removing Drugs	0.00364957	0.0162566	1	By_ingredient	0.2997
SMHB00342	蕤仁	Ruiren	Prinsepiae Nux	Hedge Prinsepia Nut	辛凉解表药	Pungent Cool Diaphoretics	0.034604	0.0559841	1	By_ingredient	0.1441
SMHB00343	三白草	Sanbaicao	Saururi Herba	Chinese Lizardtail Rhizome or Herb	利水渗湿药	Diuretic Dampness Excreting Drugs	9.837e-04	0.00715392	1	By_ingredient	0.3253
SMHB00345	三棱	Sanleng	Sparganii Rhizoma	Common Burreed Tuber	活血祛瘀药	Blood Activating Stasis Removing Drugs	0.0403691	0.0621518	1	By_ingredient	0.1541
SMHB00346	三七	Sanqi	Notoginseng Radix Et Rhizoma	Sanchi	化瘀止血药	Stasis-Resolving Hemostatic Medicinal	0.00901988	0.0254592	1	By_ingredient	0.6774
SMHB00348	桑白皮	Sangbaipi	Mori Cortex	Cortex Mori	止咳平喘药	Antitussive Antiasthmetics	0.00679856	0.0221473	1	By_ingredient	0.3125
SMHB00349	桑寄生	Sangjisheng	Taxilli Herba	Chinese Taxillus Herb	祛风湿筋骨药	Wind-Damp-Dispelling And Sinew-Bone-Strengthening Medicinal	0.00316864	0.0301757	1	By_ingredient	0.2996
SMHB00350	桑椹	Sangshen	Mori Fructus	Mulberry Fruit	补阴药	Yin-Tonifying Medicinal	9.67E-05	0.00320208	1	By_ingredient	0.6153
SMHB00351	桑叶	Sangye	Mori Folium	Mulberry Leaf	辛凉解表药	Pungent Cool Diaphoretics	0.00204346	0.0118458	1	By_ingredient	0.6542
SMHB00352	桑枝	Sangzhi	Mori Ramulus	Mulberry Twig	祛风湿清热药	Wind-Dampnessdispelling And Heatclearing Medicinal	0.024737	0.0666468	1	By_ingredient	0.3793
SMHB00353	沙棘	Shaji	Hippophae Fructus	fruit of seabuckthorn	化痰药	Phlegresolving Medicine	9.875e-04	0.00716867	1	By_ingredient	0.4233
SMHB00354	沙苑子	Shayuanzi	Astragali Complanati Semen	Flatstem Milkvetch Seed	补阳药	Yang Reinforcing Drugs	1.56E-05	3.862e-04	1	By_ingredient	0.7215
SMHB00355	砂仁	Sharen	Amomi Fructus	Villous Amomum Fruit	化湿药	Dampness Removing Drugs	0.00917815	0.0256575	1	By_ingredient	0.2914
SMHB00357	山豆根	Shandougen	Sophorae Tonkinensis Radix Et Rhizoma	Vietmese Sophora Root	清热解毒药	Antipyretic Detoxicate Drugs	0.0950888	0.119819	1	By_ingredient	0.1521
SMHB00360	山楂叶	Shanzha	Crataegi Folium	Crataegi Folium	活血祛瘀药	Blood Activating Stasis Removing Drugs	8.730e-04	0.0145602	1	By_ingredient	0.5504
SMHB00361	山茱萸	Shanzhuyu	Corni Fructus	Asiatic Cornelian Cherry Fruit	收涩药	Astringent Medicinal	6.80E-06	4.289e-04	1	By_ingredient	1.0119
SMHB00362	商陆	Shanglu	Phytolaccae Radix	Pokeberry Root	通水药	Drastic Purgatives	0.0397301	0.061471	1	By_ingredient	0.1742
SMHB00363	蛇床子	Shechuangzi	Cnidii Fructus	Common Cnidium Fruit	解毒杀虫燥湿止痒药	Medicinal For Detoxification、Parasiticide、Drying Dampness And Relieving Itching	0.0396801	0.0812362	1	By_ingredient	0.3009
SMHB00365	伸筋草	Shenjincao	Lycopodii Herba	Common Clubmoss Herb	祛风湿散寒药	Wind-Dampnessdispelling And Colddispersing Medicinal	0.0505399	0.0731361	1	By_ingredient	0.1697
SMHB00366	升麻	Shengma	Cimicifugae Rhizoma	Largetrifoliolious Bugbane Rhizome	辛凉解表药	Pungent Cool Diaphoretics	0.0034866	0.0239742	1	By_ingredient	0.5337
SMHB00367	生姜	Shengjiang	Zingiberis Rhizoma Recens	Fresh Ginger	辛温解表药	Pungent-Warm Exterior-Releasing Medicinal	0.184522	0.211628	1	By_ingredient	0.3878
SMHB00368	十大功劳根	Shidagonglaogen	None	Mahoniae Eadix	清热解毒药	Antipyretic Detoxicate Drugs	0.0242758	0.0442744	1	By_ingredient	0.146
SMHB00369	十大功劳叶	Shidagonglaoye	Folium Mahoe	leaf of leatherleaf Mahonia	清热解毒药	Antipyretic Detoxicate Drugs	0.0810722	0.105274	1	By_ingredient	0.1596
SMHB00370	石菖蒲	Shichangpu	Acori Tatarinowii Rhizoma	Grassleaf Sweetflag Rhizome	开窍药	Resuscitative Stimulant; Resuscitative Medicinal	0.0699632	0.0936856	1	By_ingredient	0.1664
SMHB00371	石见穿	Shijianchuan	None	Salviae Chinensis Herba	活血祛瘀药	Blood Activating Stasis Removing Drugs	3.18E-06	1.183e-04	0.281048	By_ingredient	0.5268
SMHB00372	石榴皮	Shiliupi	Granati Pericarpium	Pomegrate Rind	收涩药	Astringent Medicinal	0.0555879	0.0785291	1	By_ingredient	0.143
SMHB00374	石韦	Shiwei	Pyrrosiae Folium	Pyrrosiae Folium	利水渗湿药	Diuretic Dampness Excreting Drugs	0.00399126	0.032859	1	By_ingredient	0.3119
SMHB00375	使君子	Shijunzi	Quisqualis Fructus	fruit of Rangooncreeper	驱虫药	Antiparasitic Drugs	0.0397301	0.061471	1	By_ingredient	0.1624
SMHB00376	柿蒂	Shidi	Kaki Calyx	calyx and receptacle of a Persimmon	理气药	Qi Regulating Drugs	0.0397301	0.061471	1	By_ingredient	0.1497
SMHB00377	熟地黄	Shudihuang	Rehmanniae Radix Praeparata	Prepared Rehmannia Root	补血药	Blood-Tonifying Medicinal	0.0543282	0.0772065	1	By_ingredient	0.1557
SMHB00378	鼠曲草	Shuqucao	None	Gphalii Affinis Herba	化痰药	Phlegresolving Medicine	5.39E-05	9.533e-04	1	By_ingredient	0.5332
SMHB00379	水飞蓟	Shuifeiji	Silybi Fructus	Silybum Marianum	清热解毒药	Antipyretic Detoxicate Drugs	7.036e-04	0.00562902	1	By_ingredient	0.3173
SMHB00380	水红花子	Shuihonghuazi	Polygoni Orientalis Fructus	Prince's-feather Fruit	活血祛瘀药	Blood Activating Stasis Removing Drugs	9.564e-04	0.00702214	1	By_ingredient	0.2915
SMHB00381	四季青	Sijiqing	Ilicis Chinensis Folium	Purpleflower Holly Leaf	清热解毒药	Antipyretic Detoxicate Drugs	9.837e-04	0.00715392	1	By_ingredient	0.2912
SMHB00383	油松节	Songjie	Pini Lignum Nodi	Knotty pine wood, Pine nodular branch	祛风湿散寒药	Wind-Dampnessdispelling And Colddispersing Medicinal	0.061863	0.0850873	1	By_ingredient	0.1585
SMHB00385	苏合香	Suhexiang	Styrax	Storax	开窍药	Resuscitative Stimulant; Resuscitative Medicinal	0.0115912	0.0500184	1	By_ingredient	0.3075
SMHB00387	娑罗子	Suoluozi	Aesculi Semen	Buckeye Seed	理气药	Qi Regulating Drugs	1.20E-06	1.062e-04	0.494997	By_ingredient	0.6484
SMHB00388	太子参	Taizishen	Pseudostellariae Radix	Heterophylly Falsestarwort Root	补气药	Qi Reinforcing Drugs	0.0115912	0.0428653	1	By_ingredient	0.3259
SMHB00389	檀香	Tanxiang	Santali Albi Lignum	Sandalwood	理气药	Qi Regulating Drugs	0.0549582	0.0779036	1	By_ingredient	0.1599
SMHB00390	桃仁	Taoren	Persicae Semen	Peach Seed	活血祛瘀药	Blood Activating Stasis Removing Drugs	0.0132306	0.0521213	1	By_ingredient	0.3624
SMHB00391	天冬	Tiandong	Asparagi Radix	Cochinchinese Asparagus Root	补阴药	Yin-Tonifying Medicinal	2.527e-04	0.00278285	1	By_ingredient	0.2958
SMHB00393	天葵子	Tiankuizi	Semiaquilegiae Radix	Muskroot-like Semiaquilegia Root	清热解毒药	Antipyretic Detoxicate Drugs	0.0555879	0.0785291	1	By_ingredient	0.1628
SMHB00394	天南星	Tiannanxing	Arisaematis Rhizoma	Jackinthepulpit Tuber	化痰药	Phlegresolving Medicine	0.0141827	0.0537662	1	By_ingredient	0.2821
SMHB00395	天山雪莲	Tianshanxuelian	Saussureae Involucratae Herba	Saussureae Involucratae Herba	补阳药	Yang Reinforcing Drugs	0.0555879	0.0785291	1	By_ingredient	0.1359
SMHB00397	天仙子	Tianxianzi	Hyoscyami Semen	Henbane Seed	止咳平喘药	Antitussive Antiasthmetics	0.045467	0.0677509	1	By_ingredient	0.1667
SMHB00399	甜瓜子	Tianguazi	Melo Semen	muskmelon seed	化痰药	Phlegresolving Medicine	0.0164628	0.0349301	1	By_ingredient	0.1611
SMHB00401	铁线透骨草	Tiexiantougucao	None	Herba Clematidis Aethusaefoliae	祛风湿散寒药	Wind-Dampnessdispelling And Colddispersing Medicinal	0.017769	0.0366296	1	By_ingredient	0.148
SMHB00402	葶苈子	Tinglizi	Descurainiae Semen Lepidii Semen	Lepidium seed, Descurainia seed	止咳平喘药	Antitussive Antiasthmetics	0.00121603	0.00836823	1	By_ingredient	0.3131
SMHB00404	土贝母	Tubeimu	Bolbostemmatis Rhizoma	Paniculate Bolbostemma	化痰药	Phlegresolving Medicine	4.136e-04	0.00389638	1	By_ingredient	0.3756
SMHB00405	土茯苓	Tufuling	Smilacis Glabrae Rhizoma	Glabrous Greenbrier Rhizome	清热解毒药	Antipyretic Detoxicate Drugs	1.84E-05	9.416e-04	1	By_ingredient	0.676
SMHB00407	菟丝子	Tusizi	Cuscutae Semen	Dodder Seed	补阳药	Yang Reinforcing Drugs	0.0236269	0.0436071	1	By_ingredient	0.145
SMHB00409	瓦松	Wasong	Orostachyis Fimbriatae Herba	Cotyledon Fimbriata Turcz.	清热凉血药	Heat Clearing Blood Cooling Drugs	0.0190735	0.0382423	1	By_ingredient	0.1475
SMHB00410	王不留行	Wangbuliuxing	Vaccariae Semen	Vacarria seed, Cow soapwort seed	活血祛瘀药	Blood Activating Stasis Removing Drugs	1.633e-04	0.00204271	1	By_ingredient	0.2974
SMHB00411	威灵仙	Weilingxian	Clematidis Radix Et Rhizoma	Chinese Clematis Root	祛风湿散寒药	Wind-Dampnessdispelling And Colddispersing Medicinal	0.00124675	0.0084982	1	By_ingredient	0.2939
SMHB00412	委陵菜	Weilingcai	Potentiliae Chinensis Herba	Chinese Cinquefoil	清热解毒药	Antipyretic Detoxicate Drugs	0.049274	0.0716808	1	By_ingredient	0.1403
SMHB00413	乌敛莓	Wulianmei	None	Cayratiae Japonicae Herba	清热解毒药	Antipyretic Detoxicate Drugs	0.0164628	0.0349301	1	By_ingredient	0.1567
SMHB00414	乌梅	Wumei	Mume Fructus	Smoked Plum	收涩药	Astringent Medicinal	3.124e-04	0.00323282	1	By_ingredient	0.2695
SMHB00415	乌药	Wuyao	Linderae Radix	Combined Spicebush Root	理气药	Qi Regulating Drugs	0.00277519	0.01418	1	By_ingredient	0.3411
SMHB00416	无患子	Wuhuanzi	None	Sapindi Mukorossi Semen	化痰药	Phlegresolving Medicine	0.0242758	0.0442744	1	By_ingredient	0.174
SMHB00417	无患子皮	Wuhuanzipi	None	Sapindi Mukorossiperic Arpium	化痰药	Phlegresolving Medicine	2.252e-04	0.0025749	1	By_ingredient	0.3284
SMHB00418	吴茱萸	Wuzhuyu	Euodiae Fructus	Medicil Evodia Fruit	温里药	Warming Interior Drugs	0.00438529	0.0204115	1	By_ingredient	0.4643
SMHB00419	梧桐子	Wutongzi	None	Semen Firmiae	消食药	Digestants	0.00793364	0.0238489	1	By_ingredient	0.1675
SMHB00421	西河柳	Xiheliu	Tamaricis Cacumen	Chinese Tamarisk Twig	辛温解表药	Pungent-Warm Exterior-Releasing Medicinal	0.00272996	0.0140841	1	By_ingredient	0.2961
SMHB00422	西红花	Xihonghua	Croci Stigma	Saffron	活血祛瘀药	Blood Activating Stasis Removing Drugs	9.294e-04	0.00688769	1	By_ingredient	0.5828
SMHB00423	西洋参	Xiyangshen	Panacis Quinquefolii Radix	American Ginseng	补气药	Qi Reinforcing Drugs	0.0347626	0.0763505	1	By_ingredient	0.5739

Herb id	Chinese name	Pinyin name	Latin name	English name	Class in Chinese	Class in English	P_value	FDR(BH)	FDR(Bonferroni)	Relationship	Inferred evidence score
SMHB00425	细辛	Xixin	Asari Radix Et Rhizoma	all-grass Manchurian wildginger	辛温解表药	Pungent-Warm Exterior-Releasing Medicinal	0.0470428	0.0883999	1	By_ingredient	0.3384
SMHB00426	夏枯草	Xiakucao	Prunellae Spica	fruit -spike of Common Selfheal	清热泻火药	Fire Purging Drugs	7.036e-04	0.00562902	1	By_ingredient	0.3054
SMHB00428	仙鹤草	Xianhecao	Agrimoniae Herba	all - grss of Hairyvein Agrimonia	收敛止血药	Astringent Hemostatic Medicinal	0.0268674	0.0473109	1	By_ingredient	0.1382
SMHB00429	仙茅	Xianmao	Curculiginis Rhizoma	rhizome of Common Curculigo	补阳药	Yang Reinforcing Drugs	0.0499072	0.0723805	1	By_ingredient	0.181
SMHB00432	香附	Xiangfu	Cyperi Rhizoma	rhizome of Nutgrass Galingale	理气药	Qi Regulating Drugs	0.0668554	0.0903192	1	By_ingredient	0.1592
SMHB00433	香加皮	Xiangjiapi	Periplocae Cortex	Cortex Periplocae Radicis	祛风湿散寒药	Wind-Dampnessdispelling And Colddispersing Medicinal	0.0505399	0.0731361	1	By_ingredient	0.1627
SMHB00435	香薷	Xiangru	Moslae Herba	all-grass of Haichow Elsholtzia	辛温解表药	Pungent-Warm Exterior-Releasing Medicinal	0.00481158	0.0187136	1	By_ingredient	0.3022
SMHB00437	小茴香	Xiaohuixiang	Foeniculi Fructus	fennel fruit	温里药	Warming Interior Drugs	3.957e-04	0.00378978	1	By_ingredient	0.3598
SMHB00438	小蓟	Xiaoji	Cirsii Herba	Field Thistle Herb	凉血止血药	Blood-Cooling Hemostatic Medicinal	0.00859215	0.0248348	1	By_ingredient	0.1463
SMHB00439	小通草	Xiaotongcao	Stachyuri Medulla Helwingiae Medulla	Stachyurus or Japanese Helwingia Pith	利水渗湿药	Diuretic Dampness Excreting Drugs	0.0151551	0.033614	1	By_ingredient	0.1797
SMHB00440	薤白	Xiebai	Allii Macrostemonis Bulbus	bulb of longstamen onion	理气药	Qi Regulating Drugs	0.00147212	0.00952975	1	By_ingredient	0.2952
SMHB00441	辛夷	Xinyi	Magnoliae Flos	Magnoliae Flos	辛温解表药	Pungent-Warm Exterior-Releasing Medicinal	0.113075	0.138555	1	By_ingredient	0.1652
SMHB00442	徐长卿	Xuchangqing	Cynanchi Paniculati Radix Et Rhizoma	Paniculate Swallowwort Root	祛风湿散寒药	Wind-Dampnessdispelling And Colddispersing Medicinal	0.0441949	0.0663881	1	By_ingredient	0.1558
SMHB00446	旋覆花	Xuanfuhua	Inulae Flos	Inula Flower	化痰药	Phlegresolving Medicine	0.0339614	0.0552195	1	By_ingredient	0.1594
SMHB00450	亚麻子	Yamazi	Lini Semen	Linseed	润下药	Laxatives	0.0203765	0.0396878	1	By_ingredient	0.1485
SMHB00451	延胡索	Yanhusuo	Rhizoma Corydalis,Corydalis Rhizoma	Corydalis tuber	理气药	Qi Regulating Drugs	0.0499072	0.0723805	1	By_ingredient	0.1496
SMHB00452	洋金花	Yangjinhua	Daturae Flos	Datura Flower	止咳平喘药	Antitussive Antiasthmetics	0.0662327	0.0896564	1	By_ingredient	0.1634
SMHB00453	野菊花	Yejuhua	Chrysanthemi Indici Flos	Wild Chrysanthemum Flower	清热解毒药	Antipyretic Detoxicate Drugs	0.00526647	0.0371091	1	By_ingredient	0.3304
SMHB00454	一枝黄花	Yizhihuanghua	Solidaginis Herba	Solidaginis Herba	辛凉解表药	Pungent Cool Diaphoretics	0.0555879	0.0785291	1	By_ingredient	0.1537
SMHB00456	益母草	Yimucao	Leonuri Herba	motherwort	活血祛瘀药	Blood Activating Stasis Removing Drugs	5.085e-04	0.00448987	1	By_ingredient	0.3183
SMHB00457	益智	Yizhi	Alpiniae Oxyphyllae Fructus	Alpiniae Oxyphyllae Fructus	补阳药	Yang Reinforcing Drugs	0.0262201	0.0465612	1	By_ingredient	0.1671
SMHB00458	薏苡仁	Yiyiren	Coicis Semen	seed of Jobstears	利水渗湿药	Diuretic Dampness Excreting Drugs	0.0249243	0.0450476	1	By_ingredient	0.1596
SMHB00459	茵陈	Yinchen	Artemisiae Scopariae Herba	Virgate Wormwood Herb	利水渗湿药	Diuretic Dampness Excreting Drugs	0.00684284	0.0411773	1	By_ingredient	0.4056
SMHB00460	银柴胡	Yincha	Stellariae Radix	Starwort Root	清虚热药	Asthenic Heat Dispelling Drugs	0.0047837	0.0353933	1	By_ingredient	0.3291
SMHB00461	银杏叶	Yinxingye	Ginkgo Folium	Ginkgo Folium	止咳平喘药	Antitussive Antiasthmetics	0.00822123	0.0352635	1	By_ingredient	0.4455
SMHB00462	淫羊藿	Yinyanghuo	Epimedii Folium	Epimrdii Herba	补阳药	Yang Reinforcing Drugs	0.0816858	0.105937	1	By_ingredient	0.1611
SMHB00464	余甘子	Yuganzi	Phyllanthi Fructus	Emblic Leafflower Fruit	清热解毒药	Antipyretic Detoxicate Drugs	3.012e-04	0.00708489	1	By_ingredient	0.5
SMHB00465	鱼腥草	Yuxingcao	Houttuyniae Herba	all-grass of Heartleaf Houttuynia	清热解毒药	Antipyretic Detoxicate Drugs	0.0320314	0.0530252	1	By_ingredient	0.1635
SMHB00466	禹州漏芦	Yuzhouloulu	Echinopsis Radix	Globethistle Root	拔毒化腐生肌药	External Medicinal (Draw Out Toxin, Resolve Putridity)	5.085e-04	0.00448987	1	By_ingredient	0.3116
SMHB00467	玉米须	Yumixu	Stigma Maydis	Corn stigma	利水渗湿药	Diuretic Dampness Excreting Drugs	0.0624884	0.0856835	1	By_ingredient	0.1541
SMHB00468	玉竹	Yuzhu	Polygonati Odorati Rhizoma	Fragrant Solomonseal Rhizome	补阴药	Yin-Tonifying Medicinal	0.0403691	0.0621518	1	By_ingredient	0.1624
SMHB00469	郁金	Yujin	Curcumae Radix	Tuber - root of Common Turmeric	活血祛瘀药	Blood Activating Stasis Removing Drugs	4.77E-05	0.00191976	1	By_ingredient	0.9222
SMHB00470	郁李仁	Yuliren	Pruni Semen	Chinese Dwarf Cherry Seed	润下药	Laxatives	0.00793364	0.0238489	1	By_ingredient	0.1602
SMHB00471	预知子	Yuzhizi	Akebiae Fructus	Akebia Fruit	理气药	Qi Regulating Drugs	0.00793364	0.0238489	1	By_ingredient	0.169
SMHB00472	芫花	Yuanhua	Genkwa Flos	flower bud of Lilac Daphne	逐水药	Drastic Purgatives	6.351e-04	0.00523235	1	By_ingredient	0.3363
SMHB00473	芫荽	Yuansui	None	Coriandri Sativi Herba	辛温解表药	Pungent-Warm Exterior-Releasing Medicinal	0.01589	0.0343028	1	By_ingredient	0.2966
SMHB00474	月季花	Yuejihua	Rosae Chinensis Flos	Chinese rose flower	活血祛瘀药	Blood Activating Stasis Removing Drugs	0.00645658	0.0214766	1	By_ingredient	0.2936
SMHB00475	云芝	Yunzhi	Coriolus	Coriolus versicolor	拔毒化腐生肌药	External Medicinal (Draw Out Toxin, Resolve Putridity)	0.00502238	0.036271	1	By_ingredient	0.3405
SMHB00476	皂荚子	Zaojiazi	None	Gleditsiae Sinensis Semen	化痰药	Phlegresolving Medicine	0.00331277	0.0155513	1	By_ingredient	0.1974
SMHB00477	皂角刺	Zhaojiaoci	Gleditsiae Spina	spine of Chinese Honeylocust	化痰药	Phlegresolving Medicine	1.633e-04	0.00204271	1	By_ingredient	0.3073
SMHB00479	泽漆	Zeqi	None	Euphorbiae Helioscopiae Herba	利水渗湿药	Diuretic Dampness Excreting Drugs	0.00121603	0.00836823	1	By_ingredient	0.3325
SMHB00480	泽泻	Zexie	Alismatis Rhizoma	rhizome of Oriental Waterplantain	利水渗湿药	Diuretic Dampness Excreting Drugs	0.0275143	0.0481841	1	By_ingredient	0.1518
SMHB00483	栀子	Zhizi	Gardeniae Fructus	fruit of Cape Jasmine	清热泻火药	Fire Purging Drugs	0.0624884	0.0856835	1	By_ingredient	0.4142
SMHB00484	枳椇子	Zhijuzi	None	Hoveniae Dulcis Semen	利水渗湿药	Diuretic Dampness Excreting Drugs	0.0249243	0.0450476	1	By_ingredient	0.1575
SMHB00487	肿节风	Zhongjiefeng	Sarcandrae Herba	all-grass of Glabrous sarcandra	清热解毒药	Antipyretic Detoxicate Drugs	2.387e-04	0.00267491	1	By_ingredient	0.3196
SMHB00492	苎麻根	Zhumagen	Radix Boehmeriae	Ramine root	凉血止血药	Blood-Cooling Hemostatic Medicinal	0.0171161	0.0358771	1	By_ingredient	0.1413
SMHB00493	紫草	Zicao	Arnebiae Radix	Arnebia Root Gromwell Root	清热凉血药	Heat Clearing Blood Cooling Drugs	0.0333185	0.0544254	1	By_ingredient	0.1748
SMHB00494	紫花地丁	Zihuadiding	Violae Herba	Tokyo Violet Herb	清热解毒药	Antipyretic Detoxicate Drugs	0.0229776	0.0428653	1	By_ingredient	0.1604
SMHB00495	紫花前胡	Zihuaqianhu	Peucedani Decursivi Radix	Peucedanidecursivi Radix	化痰药	Phlegresolving Medicine	0.0262201	0.0465612	1	By_ingredient	0.2147
SMHB00496	紫苏叶	Zisuye	Perillae Folium	None	辛温解表药	Pungent-Warm Exterior-Releasing Medicinal	0.194904	0.22233	1	By_ingredient	0.3188
SMHB00497	紫苏子	Zisuzi	Perillae Fructus	Perilia Fruit	止咳平喘药	Antitussive Antiasthmetics	0.0816858	0.105937	1	By_ingredient	0.1551
SMHB00498	紫菀	Ziwan	Asteris Radix Et Rhizoma	root of tatarian aster	止咳平喘药	Antitussive Antiasthmetics	0.00160901	0.010122	1	By_ingredient	0.3356
SMHB00516	千里光	Qianliguang	Senecionis Scandentis Hebra	None	清热解毒药	Antipyretic Detoxicate Drugs	0.00261084	0.0272065	1	By_ingredient	0.306
SMHB00532	片姜黄	Pianjianghuang	Wenyujin Rhizoma Concisum	None	活血化瘀药	Blood-Activating And Stasis-Resolving Medicinal	0.0397008	0.0812495	1	By_ingredient	0.1879
SMHB00555	母丁香	Mudingxiang	Caryophylli Fructus	None	温里药	Warming Interior Drugs	0.0245177	0.0663698	1	By_ingredient	0.1508
SMHB00557	地龙	Dilong	Pheretima	None	平肝熄风药	Liver-Pacifying Wind-Extinguishing Medicinal	2.788e-04	0.00670912	1	By_ingredient	0.3433
SMHB00566	竹茹	Zhuru	Bambusae Caulis In Taenias	None	化痰药	Phlegresolving Medicine	0.016839	0.0577115	1	By_ingredient	0.1692
SMHB00572	合欢花	Hehuanhua	Albiziae Flos	None	安神药	Tranquilizing Medicinal	0.00521082	0.0368494	1	By_ingredient	0.1505
SMHB00575	红花龙胆	Honghualongdan	Gentianae Rhodanthae Herba	None	清热燥湿药	Heat-Clearing And Dampnessdrying Medicinal	0.016839	0.0577115	1	By_ingredient	0.1636
SMHB00576	炙红芪	Zhihongqi	Hedysari Radix Praeparata Cum Melle	None	补气药	Qi Reinforcing Drugs	0.00521082	0.0368494	1	By_ingredient	0.1916
SMHB00581	远志	Yuanzhi	Polygalae Raedix	None	安神药	Tranquilizing Medicinal	0.013608	0.0530178	1	By_ingredient	0.3676
SMHB00593	龟甲	Guijia	Testudinis Carapax Et Plastrum	None	补虚药	Tonifying And Replenishing Medicinal	0.0155535	0.0560833	1	By_ingredient	0.2544
SMHB00597	阿魏	Awei	Ferulae Resina	None	消食药	Digestants	0.0434607	0.0850163	1	By_ingredient	0.2133
SMHB00604	刺五加	Ciwujia	Acanthopanacis Senticosi Radix Et Rhizoma Seu Caulis	None	补气药	Qi Reinforcing Drugs	0.00367231	0.0319309	1	By_ingredient	0.3418
SMHB00617	制草乌	Zh1caowu	Aconiti Kusnezoffii Radix Cocta	None	祛风湿药	Wind-Dampnessdispelling Medicinal	0.0321382	0.0740937	1	By_ingredient	0.1632
SMHB00622	厚朴花	Houpohua	Magnoliae Officinalis Flos	None	化湿药	Dampness Removing Drugs	0.00521082	0.0368494	1	By_ingredient	0.176
SMHB00629	独一味	Duyiwei	Lamiophlomis Herba	None	活血祛瘀药	Blood Activating Stasis Removing Drugs	0.00202535	0.0235758	1	By_ingredient	0.319
SMHB00631	洪连	Honglion	Lagotidis Herba	None	清热解毒药	Antipyretic Detoxicate Drugs	0.0155535	0.0560833	1	By_ingredient	0.1491
SMHB00641	海龙	Hailong	Syngnathus	None	补阳药	Yang Reinforcing Drugs	0.00780633	0.0434898	1	By_ingredient	0.1733
SMHB00644	桑螵蛸	Sangpiaoxiao	Mantidis Ootheca	None	收涩药	Astringent Medicinal	0.0371863	0.0789173	1	By_ingredient	0.2172
SMHB00647	黄蜀葵花	Huangshukuihua	Abelmoschi Corolla	None	清热燥湿药	Heat-Clearing And Dampnessdrying Medicinal	0.00780633	0.0434898	1	By_ingredient	0.1478
SMHB00658	鹿茸	Lurong	Cervi Cornu Pantotrichum	None	补虚药	Tonifying And Replenishing Medicinal	0.0308721	0.0727554	1	By_ingredient	0.145
SMHB00662	棕榈	Zonglü	Trachycarpi Petiolus	None	止血药	Hemostatic Medicinal	0.00391059	0.032859	1	By_ingredient	0.1676
SMHB00672	锁阳	Suoyang	Cynomorii Herba	None	补虚药	Tonifying And Replenishing Medicinal	0.0321382	0.0740937	1	By_ingredient	0.1678
SMHB00679	蒲公英	Pugongying	Taraxaci Herba	None	清热药	Heat-Clearing Medicinal	0.00603085	0.0390382	1	By_ingredient	0.3284
SMHB00684	蜂蜜	Fengmi	Mel	None	补气药	Qi Reinforcing Drugs	1.490e-04	0.00437334	1	By_ingredient	0.3407
SMHB00687	榧子	Feizi	Torreyae Semen	None	驱虫药	Antiparasitic Drugs	0.0065094	0.0400405	1	By_ingredient	0.1881
SMHB00696	僵蚕	Jiangcan	Bombyx Batryticatus	None	平肝熄风药	Liver-Pacifying Wind-Extinguishing Medicinal	4.112e-04	0.00879653	1	By_ingredient	0.3114

S2C. CSF2 corresponding traditional Chinese medicines

Columns are reproduced from the source workbook. P_value and FDR columns report the SymMap statistical output; relationship and evidence score describe inferred gene-herb evidence.

Herb id	Chinese name	Pinyin name	Latin name	English name	Class in Chinese	Class in English	P_value	FDR(BH)	FDR(Bonferroni)	Relationship	Inferred evidence score
SMHB00013	白花蛇舌草	Baihuasheshecao	Herba Hedyotis Diffusae	all - grass of Spreading Hedyotis	清热解毒药	Antipyretic Detoxicate Drugs	0.0140032	0.0321924	1	By_ingredient	0.1689
SMHB00015	白蔹	Bailian	Ampelopsis Radix	root of Japanese Ampelopsis	清热解毒药	Antipyretic Detoxicate Drugs	0.0299343	0.0508719	1	By_ingredient	0.1798
SMHB00016	白毛藤	Baimaoteng	None	Herba Solani Lyrati	清热解毒药	Antipyretic Detoxicate Drugs	0.024548	0.0445593	1	By_ingredient	0.2163
SMHB00023	白头翁	Baitouweng	Pulsatillae Radix	Root of Chinese Pulsatilla	清热解毒药	Antipyretic Detoxicate Drugs	3.517e-04	0.00349246	1	By_ingredient	0.3539
SMHB00029	百合	Baihe	Lilii Bulbus	Bulb of Greenish Lily	补阴药	Yin-Tonifying Medicinal	0.0311279	0.0520851	1	By_ingredient	0.2211
SMHB00031	败酱草	Baijiangcao	Herba Patriniae	All - grass of Dahurian Patrinia	清热解毒药	Antipyretic Detoxicate Drugs	0.0191367	0.0383073	1	By_ingredient	0.1568
SMHB00033	板蓝根	Banlangen	Isatidis Radix	Root of Dyers Woad.	清热解毒药	Antipyretic Detoxicate Drugs	0.0695473	0.0932107	1	By_ingredient	0.1706
SMHB00036	半枝莲	Banzhilian	Scutellariae Barbatae Herba	all - grass of Barbed Skullcap	清热解毒药	Antipyretic Detoxicate Drugs	0.0299343	0.0508719	1	By_ingredient	0.1777
SMHB00037	薄荷	Bohe	Menthae Haplocalycis Herba	Mentha, Peppermint	辛凉解表药	Pungent Cool Diaphoretics	0.00165075	0.0103083	1	By_ingredient	0.3532
SMHB00048	冰片	Bingpian	Borneolum Syntheticum	Borneol	开窍药	Resuscitative Stimulant; Resuscitative Medicinal	0.0167237	0.0352718	1	By_ingredient	0.2094
SMHB00051	苍耳子	Cang'erzi	Xanthii Fructus	fruit of Siberian Cockleblur	辛温解表药	Pungent-Warm Exterior-Releasing Medicinal	0.0447664	0.0670224	1	By_ingredient	0.195
SMHB00052	苍术	Cangzhu	Atractylodis Rhizoma	Rhizome of Swordlike Atractylodes	化湿药	Dampness Removing Drugs	0.0224466	0.042184	1	By_ingredient	0.1963
SMHB00059	蝉蜕	Chantui	Cicadae Periostracum	Cicada Slough	辛凉解表药	Pungent Cool Diaphoretics	0.0266534	0.068636	1	By_ingredient	0.2127
SMHB00060	常山	Changshan	Dichroae Radix	root of antifebrile Dichroa	涌吐药	Emetic Medicinal	0.0302328	0.0511217	1	By_ingredient	0.225
SMHB00061	车前草	Cheqiancao	Plantaginis Herba	all-grass of Rippleseed plantain	利水渗湿药	Diuretic Dampness Excreting Drugs	0.0775921	0.101642	1	By_ingredient	0.1526
SMHB00063	沉香	Chenxiang	Aquilariae Lignum Resinatum	Oily wood of Agalloch Eaglewood	理气药	Qi Regulating Drugs	0.0556201	0.078542	1	By_ingredient	0.2066
SMHB00067	茺蔚子	Chongweizi	Leonuri Fructus	Motherwort Fruit	活血祛瘀药	Blood Activating Stasis Removing Drugs	0.0418152	0.0637485	1	By_ingredient	0.2048
SMHB00071	川槿皮	Chuanjinpi	None	Cortex Hibisci	清热解毒药	Antipyretic Detoxicate Drugs	0.0118829	0.029136	1	By_ingredient	0.1997
SMHB00076	川乌	Chuanwu	Aconiti Radix	root of Common Monkshood	祛风湿散寒药	Wind-Dampnessdispelling And Colddispersing Medicinal	0.0114318	0.0500184	1	By_ingredient	0.3304
SMHB00081	椿皮	Chunpi	Ailanthi Cortex	Ailanthus bark, Tree of heaven bark	清热燥湿药	Heat-Clearing And Dampnessdrying Medicinal	0.0281416	0.0488953	1	By_ingredient	0.1786
SMHB00084	大腹皮	Dafupi	Arecae Pericarpium	Areca Catechu L.	理气药	Qi Regulating Drugs	0.00468296	0.0352312	1	By_ingredient	0.3612
SMHB00085	大黄	Dahuang	Rhei Radix Et Rhizoma	root and rhziome of Sorrel Rhubarb	攻下药	Offensive Purgative Medicinal	0.0299343	0.0508719	1	By_ingredient	0.2033
SMHB00089	大血藤	Daxueteng	Sargentodoxae Caulis	Stem of Sargentgloryvine	清热解毒药	Antipyretic Detoxicate Drugs	0.00915116	0.0256322	1	By_ingredient	0.1834
SMHB00090	大枣	Dazao	Jujubae Fructus	Jujube Chinese date	补气药	Qi Reinforcing Drugs	0.047416	0.0698089	1	By_ingredient	0.1663
SMHB00092	玳玳花	Daidaihua	None	Citrus Aurantium	理气药	Qi Regulating Drugs	0.00894044	0.0454633	1	By_ingredient	0.3392
SMHB00093	丹参	Danshen	Salviae Miltiorrhizae Radix Et Rhizoma	root of Ligulilobe sage	活血祛瘀药	Blood Activating Stasis Removing Drugs	0.0652136	0.0885505	1	By_ingredient	0.1493
SMHB00094	淡豆豉	Dandouchi	Sojae Semen Praeparatum	Fermented Soybean	辛凉解表药	Pungent Cool Diaphoretics	0.0428811	0.0844673	1	By_ingredient	0.1738
SMHB00096	党参	Dangshen	Codonopsis Radix	root Pilose Asiabell	补气药	Qi Reinforcing Drugs	0.00114787	0.0171161	1	By_ingredient	0.5089
SMHB00098	倒扣草	Daokoucao	None	Achyranthis Asperae Herba	活血祛瘀药	Blood Activating Stasis Removing Drugs	0.0230473	0.0428673	1	By_ingredient	0.1618
SMHB00103	地骨皮	Digupi	Lycii Cortex	root - bark of Chinese Wolfberry	清虚热药	Asthenic Heat Dispelling Drugs	1.513e-04	0.00193805	1	By_ingredient	0.3989
SMHB00109	冬瓜皮	Dongguapi	Benincasae Exocarpium	Chinese Waxgourd Peel	利水渗湿药	Diuretic Dampness Excreting Drugs	0.0637653	0.0870238	1	By_ingredient	0.2202
SMHB00114	杜仲	Duzhong	Eucommiae Cortex	Eucommia bark	补阳药	Yang Reinforcing Drugs	0.0482979	0.070624	1	By_ingredient	0.1597
SMHB00116	断血流	Duanxueliu	Clinopodii Herba	Clinopodium Herb	凉血止血药	Blood-Cooling Hemostatic Medicinal	0.033809	0.0550182	1	By_ingredient	0.1505
SMHB00120	番泻叶	Fanxieye	Sennae Folium	Sen leaf	攻下药	Offensive Purgative Medicinal	0.00298661	0.0293845	1	By_ingredient	0.4207
SMHB00121	翻白草	Fanbaicao	Potentillae Discoloris Herba	all-grass of discolor cinquefoil	清热解毒药	Antipyretic Detoxicate Drugs	0.010973	0.0278828	1	By_ingredient	0.1765
SMHB00124	粉萆薢	Fenbixie	Dioscoreae Hypoglaucae Rhizoma	Dioscoreae Hypoglaucae Rhizoma	利水渗湿药	Diuretic Dampness Excreting Drugs	7.854e-04	0.0135839	1	By_ingredient	0.3503
SMHB00125	凤尾草	Fengweicao	Herba Pteridis Multifidae	all - grass of Chinese Brake	清热解毒药	Antipyretic Detoxicate Drugs	0.0209432	0.0404344	1	By_ingredient	0.1967
SMHB00127	凤眼草	Fengyancao	None	Ailanthi Altissimae Fructus	清热解毒药	Antipyretic Detoxicate Drugs	0.00945499	0.0260702	1	By_ingredient	0.16
SMHB00131	附子	Fuzi	Aconiti Lateralis Radix Praeparata	Common Monkshood Daughter Root	温里药	Warming Interior Drugs	0.0212441	0.0406995	1	By_ingredient	0.1899
SMHB00132	覆盆子	Fupenzi	Rubi Fructus	Rubi Fructus	收涩药	Astringent Medicinal	0.036187	0.057617	1	By_ingredient	0.1589
SMHB00133	甘草	Gancao	Glycyrrhizae Radix Et Rhizoma	Root of Ural Licorice	补气药	Qi Reinforcing Drugs	0.0835876	0.107917	1	By_ingredient	0.4096
SMHB00134	甘松	Gansong	Nardostachyos Radix Et Rhizoma	rdostachys Root	理气药	Qi Regulating Drugs	0.0212441	0.0406995	1	By_ingredient	0.1753
SMHB00137	高良姜	Gaoliangjiang	Alpiniae Officinarum Rhizoma	Alpiniae Officirum Rhizome	温里药	Warming Interior Drugs	7.354e-04	0.00582202	1	By_ingredient	0.3769
SMHB00141	钩藤	Gouteng	Uncariae Ramulus Cumuncis	Gambir Plant	平肝熄风药	Liver-Pacifying Wind-Extinguishing Medicinal	0.0284406	0.049227	1	By_ingredient	0.1793
SMHB00142	枸骨叶	Gouguye	Ilicis Cornutae Folium	Folium Ilicis Cornutae	清虚热药	Asthenic Heat Dispelling Drugs	1.472e-04	0.00433108	1	By_ingredient	0.5239
SMHB00150	绵马贯众	Mianmaguanzhong	Dryopteridis Crassirhizomatis Rhizoma	root of Chinese Angelica	清热解毒药	Antipyretic Detoxicate Drugs	0.0449787	0.086568	1	By_ingredient	0.1722
SMHB00155	桂枝	Guizi	Cinnamomi Ramulus	Cassia Twig	辛温解表药	Pungent-Warm Exterior-Releasing Medicinal	0.0181608	0.0589026	1	By_ingredient	0.3621
SMHB00162	核桃仁	Hetaoren	Juglandis Semen	English Walnut Seed	补阳药	Yang Reinforcing Drugs	0.0299343	0.0508719	1	By_ingredient	0.2092
SMHB00169	红芪	Hongqi	Hedysari Radix	Hedysari Radix	补气药	Qi Reinforcing Drugs	0.00562847	0.0382709	1	By_ingredient	0.3437
SMHB00177	虎杖	Huzhang	Polygoni Cuspidati Rhizoma Et Radix	rhizome of Gaint Knotweed	利水渗湿药	Diuretic Dampness Excreting Drugs	0.00627878	0.0395886	1	By_ingredient	0.3723
SMHB00179	花生衣	Huashengyi	Testa Arachidis Hypogaeae	Semino-dermis Arachidis	收敛止血药	Astringent Hemostatic Medicinal	0.0238048	0.0654912	1	By_ingredient	0.1859
SMHB00181	化橘红	Huajuhong	Citri Grandis Exocarpium	Pummelo Peel	理气药	Qi Regulating Drugs	0.0308296	0.0517563	1	By_ingredient	0.1864
SMHB00184	黄柏	Huangbo	Phellodendri Chinensis Cortex	Phellodendron bark	清热燥湿药	Heat-Clearing And Dampnessdrying Medicinal	0.0471219	0.069479	1	By_ingredient	0.1492
SMHB00185	黄精	Huangjing	Polygonati Rhizoma	Rhizome of Fragrant Solomonseal	补阴药	Yin-Tonifying Medicinal	0.0484651	0.0894661	1	By_ingredient	0.2136
SMHB00189	黄药子	Huangyaozi	Rhizoma Dioscoreae Bulbiferae	rhizome of Airpotato Yam	化痰药	Phlegresolving Medicine	0.022146	0.0418121	1	By_ingredient	0.205
SMHB00191	鸡骨草	Jigucao	Abri Herba	Canton Love-pea Vine	利水渗湿药	Diuretic Dampness Excreting Drugs	1.069e-04	0.00152878	1	By_ingredient	0.3655
SMHB00193	鸡屎藤	Jishiteng	None	Paederiae Scandentis Herba	消食药	Digestants	0.0429966	0.0650425	1	By_ingredient	0.1509
SMHB00194	鸡血藤	Jixueteng	Spatholobi Caulis	Suberect Spatholobus Stem	活血祛瘀药	Blood Activating Stasis Removing Drugs	0.00102135	0.00734653	1	By_ingredient	0.3508
SMHB00195	积雪草	Jixuecao	Centellae Herba	Asiatic Pennywort Herb	清热解毒药	Antipyretic Detoxicate Drugs	0.00436504	0.0342023	1	By_ingredient	0.3372
SMHB00197	蒺藜	Jili	Tribuli Fructus	Puncturevine Caltrop Fruit	平肝熄风药	Liver-Pacifying Wind-Extinguishing Medicinal	0.0218455	0.041379	1	By_ingredient	0.1964
SMHB00203	金果榄	Jinguolan	Tinosporae Radix	Tinosporae Radix	清热解毒药	Antipyretic Detoxicate Drugs	0.0176292	0.0365123	1	By_ingredient	0.1813
SMHB00204	金莲花	Jinlianhua	Flos Trollii Chinensis	flower of Chinese Globeflower	清热解毒药	Antipyretic Detoxicate Drugs	0.0206424	0.040038	1	By_ingredient	0.1744
SMHB00206	金荞麦	Jinqiaomai	Fagopyri Dibotryis Rhizoma	Wild Buckwheat Rhizome	清热解毒药	Antipyretic Detoxicate Drugs	0.00387825	0.032859	1	By_ingredient	0.3777
SMHB00207	金银花	Jinyinhua	Lonicerae Japonicae Flos	Honeysuckle Flower	清热解毒药	Antipyretic Detoxicate Drugs	0.00302465	0.029563	1	By_ingredient	0.5458
SMHB00208	金樱子	Jinyingzi	Rosae Laevigatae Fructus	fruit of Cherokee Rose	收涩药	Astringent Medicinal	0.022747	0.0425432	1	By_ingredient	0.1427
SMHB00212	荆芥	Jingjie	Schizonepetae Herba	all-grass of Fineleaf Schizonepeta	辛温解表药	Pungent-Warm Exterior-Releasing Medicinal	0.0623151	0.0855759	1	By_ingredient	0.1729
SMHB00213	荆芥穗	Jingjiesui	Schizonepetae Spica	Schizonepetae Spica	辛温解表药	Pungent-Warm Exterior-Releasing Medicinal	0.0215448	0.0410514	1	By_ingredient	0.1596
SMHB00214	景天三七	Jintiansanqi	None	Sedum Aizoon L.	化瘀止血药	Stasis-Resolving Hemostatic Medicinal	0.054744	0.0776983	1	By_ingredient	0.1648
SMHB00216	桔梗	Jiegeng	Platycodonis Radix	Platycodon Root	化痰药	Phlegresolving Medicine	0.00577013	0.0387566	1	By_ingredient	0.4057
SMHB00224	决明子	Juemingzi	Cassiae Semen	Cassia Seed	清热泻火药	Fire Purging Drugs	0.0453558	0.0676285	1	By_ingredient	0.1945
SMHB00227	苦丁茶	Kudingcha	None	Ilicis Cornutae Folium Immayuri	清热泻火药	Fire Purging Drugs	0.0482979	0.070624	1	By_ingredient	0.1665
SMHB00230	苦杏仁	Kuxingren	Armeniacae Semen Amarum	bitter Apricot seed	止咳平喘药	Antitussive Antiasthmetics	0.047416	0.0698089	1	By_ingredient	0.1737
SMHB00232	昆布	Kunbu	Laminariae Thallus Eckloniae Thallus	Kelp or Tangle	化痰药	Phlegresolving Medicine	0.00243799	0.0261648	1	By_ingredient	0.3714
SMHB00234	莱菔子	Laifuzi	Raphani Semen	Radish Seed	消食药	Digestants	0.0215448	0.0410514	1	By_ingredient	0.1528
SMHB00236	了哥王根	Liaogewanggen	None	Wikstroemiae Indicae Rasix	清热解毒药	Antipyretic Detoxicate Drugs	0.0245177	0.0663698	1	By_ingredient	0.1914
SMHB00237	雷公藤	Leigongteng	Radix Tripterygii Wilfordii	root of common htreewingnut	祛风湿散寒药	Wind-Dampnessdispelling And Colddispersing Medicinal	0.0538671	0.0766655	1	By_ingredient	0.171
SMHB00238	雷丸	Leiwan	Omphalia	Thunder Ball	驱虫药	Antiparasitic Drugs	0.0238048	0.0654912	1	By_ingredient	0.1704

Herb id	Chinese name	Pinyin name	Latin name	English name	Class in Chinese	Class in English	P_value	FDR(BH)	FDR(Bonferroni)	Relationship	Inferred evidence score
SMHB00240	连钱草	Lianqiancao	Glechomae Herba	Glechomae Herba	利水渗湿药	Diuretic Dampness Excreting Drugs	0.0462392	0.0685537	1	By_ingredient	0.1681
SMHB00241	连翘	Lianqiao	Forsythiae Fructus	Weeping Forsythia Capsule	清热解毒药	Antipyretic Detoxicate Drugs	0.0573705	0.0803054	1	By_ingredient	0.1961
SMHB00249	凌霄花	Lingxiaohua	Campsis Flos	flower of Common Trumpetcreeper	活血祛瘀药	Blood Activating Stasis Removing Drugs	0.0573705	0.0803054	1	By_ingredient	0.1525
SMHB00250	零陵香	Linglingxiang	None	Lysimachiae Foenigraeci Herba	辛温解表药	Pungent-Warm Exterior-Releasing Medicinal	0.0591181	0.0821997	1	By_ingredient	0.1633
SMHB00251	龙胆	Longdan	Gentianae Radix Et Rhizoma	Gentiae Radix Et Rhozima	清热燥湿药	Heat-Clearing And Dampnessdrying Medicinal	0.0203414	0.0396369	1	By_ingredient	0.1867
SMHB00254	漏芦	Loulu	Rhapontici Radix	Uniflower Swisscentaury Root	清热解毒药	Antipyretic Detoxicate Drugs	0.00283173	0.0284937	1	By_ingredient	0.375
SMHB00257	鹿衔草	Luxiancao	Pyrolae Herba	all-grass Chinese pyrola	祛风湿散寒药	Wind-Dampnessdispelling And Colddispersing Medicinal	0.0197392	0.0389162	1	By_ingredient	0.1623
SMHB00258	路路通	Lulutong	Liquidambaris Fructus	fruit of beautiful sweetgum	祛风湿散寒药	Wind-Dampnessdispelling And Colddispersing Medicinal	0.00580387	0.0204115	1	By_ingredient	0.1899
SMHB00264	麻黄	Mahuang	Ephedrae Herba	Ephedra	辛温解表药	Pungent-Warm Exterior-Releasing Medicinal	0.110306	0.135612	1	By_ingredient	0.3301
SMHB00266	马鞭草	Mabiancao	Verbenae Herba	European Verbe Herb	活血祛瘀药	Blood Activating Stasis Removing Drugs	0.0594091	0.0825459	1	By_ingredient	0.1498
SMHB00270	马钱子	Maqianzi	Strychni Semen	Nux Vomica	祛风湿散寒药	Wind-Dampnessdispelling And Colddispersing Medicinal	0.0215448	0.0410514	1	By_ingredient	0.162
SMHB00273	满山红	Manshanhong	Rhododendri Daurici Folium	Dahurian Rhododendron Leaf	止咳平喘药	Antitussive Antiasthmetics	0.0532821	0.0760964	1	By_ingredient	0.1761
SMHB00280	猕猴桃根	Mihoutaogen	None	Actinidia Chinensis Planch	清热解毒药	Antipyretic Detoxicate Drugs	1.282e-04	0.00173447	1	By_ingredient	0.3779
SMHB00283	墨旱莲	Mohanlian	Ecliptae Herba	all-grass of Yerbadetajo	补阴药	Yin-Tonifying Medicinal	0.0018585	0.0226717	1	By_ingredient	0.3628
SMHB00287	木鳖子	Mubiezi	Momordicae Semen	Momordicae Semen	拔毒化腐生肌药	External Medicinal (Draw Out Toxin, Resolve Putridity)	0.0124891	0.0300251	1	By_ingredient	0.1688
SMHB00288	木瓜	Mugua	Chaenomelis Fructus	fruit of Common Floweringquince	祛风湿散寒药	Wind-Dampnessdispelling And Colddispersing Medicinal	0.013874	0.047423	1	By_ingredient	0.3682
SMHB00293	木贼	Muzei	Equiseti Hiemalis Herba	Common Scouring Rush Herb	辛凉解表药	Pungent Cool Diaphoretics	0.029337	0.0501549	1	By_ingredient	0.1753
SMHB00303	牛黄	Niuhuang	Bovis Calculus	Cow-bezoar	开窍药	Resuscitative Stimulant; Resuscitative Medicinal	0.0146083	0.0328962	1	By_ingredient	0.1763
SMHB00306	女贞子	Nüzhenzi	Ligustri Lucidi Fructus	fruit of Glossy privet	补阴药	Yin-Tonifying Medicinal	0.0374916	0.0792393	1	By_ingredient	0.3534
SMHB00310	枇杷叶	Pipaye	Eriobotryae Folium	Loquat Leaf	止咳平喘药	Antitussive Antiasthmetics	0.00213481	0.0160937	1	By_ingredient	0.5447
SMHB00312	蒲黄	Puhuang	Typhae Pollen	pollen of longbract cattail	化瘀止血药	Stasis-Resolving Hemostatic Medicinal	0.0182324	0.037149	1	By_ingredient	0.1603
SMHB00314	千斤拔	Qianjinba	Radix Moghaniae Philippinensis	root of Philippine Flemingia	祛风湿筋骨药	Wind-Damp-Dispelling And Sinew-Bone-Strengthening Medicinal	0.00248567	0.0264248	1	By_ingredient	0.3613
SMHB00318	牵牛子	Qianniuzi	Pharbitidis Semen	Pharbitis seed	通水药	Drastic Purgatives	0.0287395	0.0495167	1	By_ingredient	0.1896
SMHB00319	前胡	Qianhu	Peucedani Radix	root of Grand Hogfennel	化痰药	Phlegresolving Medicine	0.0477101	0.0700854	1	By_ingredient	0.1546
SMHB00325	秦皮	Qinpi	Fraxini Cortex	bark of Largeleaf Chinese Ash	清热燥湿药	Heat-Clearing And Dampnessdrying Medicinal	0.0302328	0.0511217	1	By_ingredient	0.1665
SMHB00334	瞿麦	Qumai	Dianthi Herba	all-grass of Lilac Pink	利水渗湿药	Diuretic Dampness Excreting Drugs	2.089e-04	0.00244395	1	By_ingredient	0.3676
SMHB00336	人参	Renshen	Ginseng Radix Et Rhizoma	Ginseng	补气药	Qi Reinforcing Drugs	0.043646	0.0851449	1	By_ingredient	0.3792
SMHB00342	蕤仁	Ruiren	Prinsepiae Nux	Hedge Prinsepia Nut	辛凉解表药	Pungent Cool Diaphoretics	0.0161197	0.0345662	1	By_ingredient	0.1628
SMHB00348	桑白皮	Sangbaipi	Mori Cortex	Cortex Mori	止咳平喘药	Antitussive Antiasthmetics	0.0567873	0.079705	1	By_ingredient	0.1627
SMHB00351	桑叶	Sangye	Mori Folium	Mulberry Leaf	辛凉解表药	Pungent Cool Diaphoretics	0.044678	0.0862047	1	By_ingredient	0.2967
SMHB00353	沙棘	Shaji	Hippophae Fructus	fruit of seabuckthorn	化痰药	Phlegresolving Medicine	0.0909656	0.115522	1	By_ingredient	0.1525
SMHB00357	山豆根	Shandougen	Sophorae Tonkinensis Radix Et Rhizoma	Vietmese Sophora Root	清热解毒药	Antipyretic Detoxicate Drugs	8.556e-04	0.00650223	1	By_ingredient	0.3761
SMHB00360	山楂叶	Shanzha	Crataegi Folium	Crataegi Folium	活血祛瘀药	Blood Activating Stasis Removing Drugs	3.25398e-06	2.408e-04	1	By_ingredient	0.7711
SMHB00361	山茱萸	Shanzhuyu	Corni Fructus	Asiatic Cornelian Cherry Fruit	收涩药	Astringent Medicinal	0.0878502	0.11231	1	By_ingredient	0.1555
SMHB00370	石菖蒲	Shichangpu	Acori Tatarinowii Rhizoma	Grassleaf Sweetflag Rhizome	开窍药	Resuscitative Stimulant; Resuscitative Medicinal	0.032916	0.0539574	1	By_ingredient	0.2198
SMHB00371	石见穿	Shijianchuan	None	Salviae Chinensis Herba	活血祛瘀药	Blood Activating Stasis Removing Drugs	0.0137005	0.0317324	1	By_ingredient	0.1767
SMHB00372	石榴皮	Shiliupi	Granati Pericarpium	Pomegrate Rind	收涩药	Astringent Medicinal	0.0260467	0.0463845	1	By_ingredient	0.1658
SMHB00376	柿蒂	Shidi	Kaki Calyx	calyx and receptacle of a Persimmon	理气药	Qi Regulating Drugs	0.0185339	0.0375488	1	By_ingredient	0.1726
SMHB00378	鼠曲草	Shuqucao	None	Gphalii Affinis Herba	化痰药	Phlegresolving Medicine	0.0347013	0.0560861	1	By_ingredient	0.1703
SMHB00379	水飞蓟	Shuifeiji	Silybi Fructus	Silybum Marianum	清热解毒药	Antipyretic Detoxicate Drugs	0.0182324	0.037149	1	By_ingredient	0.1818
SMHB00380	水红花子	Shuihonghuazi	Polygoni Orientalis Fructus	Prince's-feather Fruit	活血祛瘀药	Blood Activating Stasis Removing Drugs	0.0212441	0.0406995	1	By_ingredient	0.186
SMHB00381	四季青	Sijiqing	Ilicis Chinensis Folium	Purpleflower Holly Leaf	清热解毒药	Antipyretic Detoxicate Drugs	0.0215448	0.0410514	1	By_ingredient	0.166
SMHB00383	油松节	Songjie	Pini Lignum Nodi	Knotty pine wood, Pine nodular branch	祛风湿散寒药	Wind-Dampnessdispelling And Colddispersing Medicinal	0.0290383	0.0497964	1	By_ingredient	0.1865
SMHB00388	太子参	Taizishen	Pseudostellariae Radix	Heterophylly Falsestarwort Root	补气药	Qi Reinforcing Drugs	0.0106695	0.0275272	1	By_ingredient	0.1748
SMHB00404	土贝母	Tubeimu	Bolbostemmatis Rhizoma	Paniculate Bolbostemma	化痰药	Phlegresolving Medicine	0.0140032	0.0321924	1	By_ingredient	0.2028
SMHB00405	土茯苓	Tufuling	Smilacis Glabrae Rhizoma	Glabrous Greenbrier Rhizome	清热解毒药	Antipyretic Detoxicate Drugs	1.62636e-06	1.366e-04	0.672529	By_ingredient	0.7989
SMHB00412	委陵菜	Weilingcai	Potentiliae Chinensis Herba	Chinese Cinquefoil	清热解毒药	Antipyretic Detoxicate Drugs	0.0230473	0.0428673	1	By_ingredient	0.1396
SMHB00414	乌梅	Wumei	Mume Fructus	Smoked Plum	收涩药	Astringent Medicinal	0.012186	0.0295348	1	By_ingredient	0.145
SMHB00421	西河柳	Xiheliu	Tamaricis Cacumen	Chinese Tamarisk Twig	辛温解表药	Pungent-Warm Exterior-Releasing Medicinal	0.03589	0.057326	1	By_ingredient	0.1744
SMHB00426	夏枯草	Xiakucao	Prunellae Spica	fruit -spike of Common Selfheal	清热泻火药	Fire Purging Drugs	0.0182324	0.037149	1	By_ingredient	0.1689
SMHB00428	仙鹤草	Xianhecao	Agrimoniae Herba	all - grss of Hairyvein Agrimonia	收敛止血药	Astringent Hemostatic Medicinal	0.0124891	0.0300251	1	By_ingredient	0.1618
SMHB00433	香加皮	Xiangjiapi	Periplocae Cortex	Cortex Periplocae Radicis	祛风湿散寒药	Wind-Dampnessdispelling And Colddispersing Medicinal	0.0236478	0.0436152	1	By_ingredient	0.1516
SMHB00435	香薷	Xiangru	Moslae Herba	all-grass of Haichow Elsholtzia	辛温解表药	Pungent-Warm Exterior-Releasing Medicinal	0.0113346	0.0500184	1	By_ingredient	0.3592
SMHB00443	续断	Xuduan	Dipsaci Radix	Himalayan Teasel Root	补阳药	Yang Reinforcing Drugs	0.00884725	0.0251881	1	By_ingredient	0.181
SMHB00445	玄参	Xuanshen	Scrophulariae Radix	Figwort Root	清热凉血药	Heat Clearing Blood Cooling Drugs	0.0140032	0.0321924	1	By_ingredient	0.172
SMHB00451	延胡索	Yanhusuo	Rhizoma Corydalis,Corydalis Rhizoma	Corydalis tuber	理气药	Qi Regulating Drugs	0.0233476	0.0432519	1	By_ingredient	0.1665
SMHB00453	野菊花	Yejuhua	Chrysanthemi Indici Flos	Wild Chrysanthemum Flower	清热解毒药	Antipyretic Detoxicate Drugs	0.00915116	0.0256322	1	By_ingredient	0.1715
SMHB00461	银杏叶	Yinxingye	Ginkgo Folium	Ginkgo Folium	止咳平喘药	Antitussive Antiasthmetics	0.0222821	0.0642963	1	By_ingredient	0.3586
SMHB00462	淫羊藿	Yinyanghuo	Epimedii Folium	Epimrdii Herba	补阳药	Yang Reinforcing Drugs	0.0385601	0.0602856	1	By_ingredient	0.1993
SMHB00467	玉米须	Yumixu	Stigma Maydis	Corn stigma	利水渗湿药	Diuretic Dampness Excreting Drugs	0.029337	0.0501549	1	By_ingredient	0.155
SMHB00470	郁李仁	Yuliren	Pruni Semen	Chinese Dwarf Cherry Seed	润下药	Laxatives	0.00366888	0.0162566	1	By_ingredient	0.1766
SMHB00471	预知子	Yuzhizi	Akebiae Fructus	Akebia Fruit	理气药	Qi Regulating Drugs	0.0393753	0.0809744	1	By_ingredient	0.1572
SMHB00474	月季花	Yuejihua	Rosae Chinensis Flos	Chinese rose flower	活血祛瘀药	Blood Activating Stasis Removing Drugs	0.0553282	0.0782948	1	By_ingredient	0.1483
SMHB00478	泽兰	Zelan	Lycopi Herba	Hirsute Shiny Bugleweed Herb	活血祛瘀药	Blood Activating Stasis Removing Drugs	0.00488934	0.0188222	1	By_ingredient	0.1708
SMHB00480	泽泻	Zexie	Alismatis Rhizoma	rhizome of Oriental Waterplantain	利水渗湿药	Diuretic Dampness Excreting Drugs	0.012792	0.0304107	1	By_ingredient	0.1947
SMHB00483	栀子	Zhizi	Gardeniae Fructus	fruit of Cape Jasmine	清热泻火药	Fire Purging Drugs	0.018641	0.0501549	1	By_ingredient	0.3783
SMHB00484	枳椇子	Zhijuzi	None	Hoveniae Dulcis Semen	利水渗湿药	Diuretic Dampness Excreting Drugs	0.0115796	0.0287235	1	By_ingredient	0.1881
SMHB00492	苎麻根	Zhumagen	Radix Boehmeriae	Ramine root	凉血止血药	Blood-Cooling Hemostatic Medicinal	0.00793505	0.0238489	1	By_ingredient	0.1767
SMHB00493	紫草	Zicao	Arnebiae Radix	Arnebia Root Gromwell Root	清热凉血药	Heat Clearing Blood Cooling Drugs	0.0155154	0.0339576	1	By_ingredient	0.1844
SMHB00496	紫苏叶	Zisuye	Perillae Folium	None	辛温解表药	Pungent-Warm Exterior-Releasing Medicinal	0.0327768	0.0747006	1	By_ingredient	0.3264
SMHB00498	紫菀	Ziwan	Asteris Radix Et Rhizoma	root of tatarian aster	止咳平喘药	Antitussive Antiasthmetics	0.0275434	0.0481923	1	By_ingredient	0.1882
SMHB00512	山麦冬	Shanmaidong	Liriopes Radix	None	补阴药	Yin-Tonifying Medicinal	0.0159301	0.0565385	1	By_ingredient	0.1433
SMHB00516	千里光	Qianliguang	Senecionis Scandentis Hebra	None	清热解毒药	Antipyretic Detoxicate Drugs	8.131e-04	0.0138253	1	By_ingredient	0.3921
SMHB00518	川木香	Chuanmuxiang	Ⅴladimiriae Radix	None	理气药	Qi Regulating Drugs	9.285e-04	0.0148746	1	By_ingredient	0.3622
SMHB00521	广金钱草	Guangjinqiancao	Desmodii Styracifolii Herba	None	利水渗湿药	Diuretic Dampness Excreting Drugs	0.0435808	0.0851449	1	By_ingredient	0.2049
SMHB00536	水蛭	Shuizhi	Hirudo	None	活血化瘀药	Blood-Activating And Stasis-Resolving Medicinal	0.0252301	0.067052	1	By_ingredient	0.1877
SMHB00589	何首乌	Heshouwu	Polygoni Multiflori Radix	None	补虚药	Tonifying And Replenishing Medicinal	0.00417932	0.0337822	1	By_ingredient	0.4627
SMHB00590	制何首乌	Zhiheshouwu	Polgoni Multiflori Radix Praeparata	None	补虚药	Tonifying And Replenishing Medicinal	0.0302032	0.07204	1	By_ingredient	0.1867
SMHB00597	阿魏	Awei	Ferulae Resina	None	消食药	Digestants	0.0245177	0.0663698	1	By_ingredient	0.1939

Herb id	Chinese name	Pinyin name	Latin name	English name	Class in Chinese	Class in English	P_value	FDR(BH)	FDR(Bonferroni)	Relationship	Inferred evidence score
SMHB00617	制草乌	Zh1caowu	Aconiti Kusnezoffii Radix Cocta	None	祛风湿药	Wind-Dampnessdispelling Medicinal	0.0180837	0.0588421	1	By_ingredient	0.1892
SMHB00630	首乌藤	Shouwuteng	Polygoni Multiflori Caulis	None	安神药	Tranguilizing Medicinal	0.00436865	0.0342023	1	By_ingredient	0.2018
SMHB00664	雄黄	Xionghuang	Realgar	None	攻毒杀虫止痒药	Toxin-Attacking And Worm-Expelling And Itch-Relieving Medicinal	0.00582084	0.0388881	1	By_ingredient	0.1956
SMHB00672	锁阳	Suoyang	Cynomorii Herba	None	补虚药	Tonifying And Replenishing Medicinal	0.0180837	0.0588421	1	By_ingredient	0.1619

S2D. FN1 corresponding traditional Chinese medicines

Columns are reproduced from the source workbook. P_value and FDR columns report the SymMap statistical output; relationship and evidence score describe inferred gene-herb evidence.

Herb id	Chinese name	Pinyin name	Latin name	English name	Class in Chinese	Class in English	P_value	FDR(BH)	FDR(Bonferroni)	Relationship	Inferred evidence score
SMHB00001	矮地茶	Aidicha	Ardisiae Japonicae Herba	Japanese Ardisia Herb	止咳平喘药	Antitussive Antiasthmetics	0.00572403	0.0386568	1	By_ingredient	0.5705
SMHB00025	白鲜皮	Baixianpi	Dictamni Cortex	Dictamni Cortex	清热燥湿药	Heat-Clearing And Dampnessdrying Medicinal	0.0624513	0.0856618	1	By_ingredient	0.1658
SMHB00026	白药子	Baiyaozi	None	Radix Stephaniae Cepharanthae.	清热解毒药	Antipyretic Detoxicate Drugs	5.901e-04	0.0111907	1	By_ingredient	0.5361
SMHB00029	百合	Baihe	Lilii Bulbus	Bulb of Greenish Lily	补阴药	Yin-Tonifying Medicinal	0.013468	0.0525913	1	By_ingredient	0.4531
SMHB00035	半夏	Banxia	Pinelliae Rhizoma	tuber of Pinellia	化痰药	Phlegresolving Medicine	0.0329771	0.0747138	1	By_ingredient	0.3443
SMHB00036	半枝莲	Banzhilian	Scutellariae Barbatae Herba	all - grass of Barbed Skullcap	清热解毒药	Antipyretic Detoxicate Drugs	0.00410817	0.0334136	1	By_ingredient	0.5947
SMHB00040	北沙参	Beishashen	Glehniae Radix	root of Coastal Glehnia	补阴药	Yin-Tonifying Medicinal	0.039542	0.0811999	1	By_ingredient	0.3362
SMHB00050	蚕沙	Cansha	None	Bombyx Mori L.	祛风湿散寒药	Wind-Dampnessdispelling And Colddispersing Medicinal	0.100966	0.125939	1	By_ingredient	0.1537
SMHB00052	苍术	Cangzhu	Atractylodis Rhizoma	Rhizome of Swordlike Atractylodes	化湿药	Dampness Removing Drugs	0.0216149	0.0635209	1	By_ingredient	0.3534
SMHB00062	车前子	Cheqianzi	Plantaginis Semen	seed of Asiatic pantain	利水渗湿药	Diuretic Dampness Excreting Drugs	0.00458063	0.0349741	1	By_ingredient	0.3312
SMHB00063	沉香	Chenxiang	Aquilariae Lignum Resinatum	Oily wood of Agalloch Eaglewood	理气药	Qi Regulating Drugs	0.108162	0.13344	1	By_ingredient	0.2133
SMHB00066	赤小豆	Chixiaodou	Vignae Semen	Rice Bean	利水渗湿药	Diuretic Dampness Excreting Drugs	0.022429	0.0421814	1	By_ingredient	0.2139
SMHB00067	茺蔚子	Chongweizi	Leonuri Fructus	Motherwort Fruit	活血祛瘀药	Blood Activating Stasis Removing Drugs	0.081894	0.106128	1	By_ingredient	0.1461
SMHB00069	楮实子	Chushizi	Broussonetiae Fructus	Broussonetiae Fructus	补阴药	Yin-Tonifying Medicinal	0.0207818	0.0623205	1	By_ingredient	0.358
SMHB00070	川贝母	Chuanbeimu	Fritillariae Cirrhosae Bulbus	Bulb of Tendrilleaf Fritillary	化痰药	Phlegresolving Medicine	1.502e-04	0.00439491	1	By_ingredient	0.73
SMHB00074	川牛膝	Chuanniuxi	Cyathulae Radix	root of Medicil cyathula	活血祛瘀药	Blood Activating Stasis Removing Drugs	0.0284112	0.0492021	1	By_ingredient	0.1872
SMHB00075	川射干	Chuanshegan	Iridis Tectori Rhizoma	Iridis Tectori Rhizoma	清热解毒药	Antipyretic Detoxicate Drugs	0.0346591	0.0762711	1	By_ingredient	0.3397
SMHB00076	川乌	Chuanwu	Aconiti Radix	root of Common Monkshood	祛风湿散寒药	Wind-Dampnessdispelling And Colddispersing Medicinal	0.00525687	0.0370522	1	By_ingredient	0.4918
SMHB00079	穿心莲	Chuanxinlian	Andrographis Herba	all-grass of Common Androgrphis	清热解毒药	Antipyretic Detoxicate Drugs	0.0566614	0.0796079	1	By_ingredient	0.2003
SMHB00082	葱白	Congbai	Bulbus Allii Fistulosi	Allium bulb, Wild scallion, Chinese green onion	辛温解表药	Pungent-Warm Exterior-Releasing Medicinal	0.072791	0.0967045	1	By_ingredient	0.1802
SMHB00087	大青叶	Daqingye	Isatidis Folium	Isatis leaf	清热解毒药	Antipyretic Detoxicate Drugs	0.0125685	0.0513661	1	By_ingredient	0.3884
SMHB00088	大蒜	Dasuan	Allii Sativi Bulbus	Garlic	解毒杀虫燥湿止痒药	Medicinal For Detoxification、Parasiticide、Drying Dampness And Relieving Itching	0.00183181	0.0225237	1	By_ingredient	0.5639
SMHB00092	玳玳花	Daidaihua	None	Citrus Aurantium	理气药	Qi Regulating Drugs	0.00367532	0.0319309	1	By_ingredient	0.5217
SMHB00094	淡豆豉	Dandouchi	Sojae Semen Praeparatum	Fermented Soybean	辛凉解表药	Pungent Cool Diaphoretics	0.00403769	0.0304107	1	By_ingredient	0.3321
SMHB00096	党参	Dangshen	Codonopsis Radix	root Pilose Asiabell	补气药	Qi Reinforcing Drugs	0.0105547	0.0485443	1	By_ingredient	0.4836
SMHB00108	冬虫夏草	Dongchongxiacao	Cordyceps	Chinese Caterpillar Fungus	补阳药	Yang Reinforcing Drugs	0.0241959	0.0658439	1	By_ingredient	0.3485
SMHB00114	杜仲	Duzhong	Eucommiae Cortex	Eucommia bark	补阳药	Yang Reinforcing Drugs	0.0942769	0.118962	1	By_ingredient	0.335
SMHB00122	防风	Fangfeng	Saposhnikoviae Radix	root of Divaricate Saposhnikovia	辛温解表药	Pungent-Warm Exterior-Releasing Medicinal	0.100411	0.125405	1	By_ingredient	0.2087
SMHB00129	茯苓	Fuling	Poria	Indian Bread	利水渗湿药	Diuretic Dampness Excreting Drugs	0.057822	0.0808248	1	By_ingredient	0.2229
SMHB00135	甘遂	Gansui	Kansui Radix	root of Gansui	通水药	Drastic Purgatives	0.0319844	0.0529756	1	By_ingredient	0.2349
SMHB00139	葛花	Gehua	Flos Puerariae Lobatae	flower bud of lobed kudzuvine	辛凉解表药	Pungent Cool Diaphoretics	0.124025	0.14963	1	By_ingredient	0.1613
SMHB00140	功劳木	Gonglaomu	Mahoniae Caulis	Stem of Leatherleaf Mahonia	清热解毒药	Antipyretic Detoxicate Drugs	0.0560807	0.0790508	1	By_ingredient	0.1694
SMHB00142	枸骨叶	Gouguye	Ilicis Cornutae Folium	Folium Ilicis Cornutae	清虚热药	Asthenic Heat Dispelling Drugs	0.0213357	0.0632148	1	By_ingredient	0.3386
SMHB00147	瓜蒌	Gualou	Trichosanthis Fructus	Trichosanthes Kirilowii Maxim	化痰药	Phlegresolving Medicine	0.0202342	0.0614834	1	By_ingredient	0.3605
SMHB00148	关黄柏	Guanhuangbo	Phellodendri Amurensis Cortex	Phellodendri Amurensis Cortex	清热燥湿药	Heat-Clearing And Dampnessdrying Medicinal	0.00184636	0.0110579	1	By_ingredient	0.5095
SMHB00150	绵马贯众	Mianmaguanzhong	Dryopteridis Crassirhizomatis Rhizoma	root of Chinese Angelica	清热解毒药	Antipyretic Detoxicate Drugs	0.0414541	0.0633926	1	By_ingredient	0.3387
SMHB00153	鬼箭羽	Guijianyu	Ramulus Euonymi	twig of Winged Euonymus	活血祛瘀药	Blood Activating Stasis Removing Drugs	0.00327168	0.0307771	1	By_ingredient	0.6205
SMHB00158	海桐皮	Haitongpi	Cortex Erythrie	bark of Oriental Variegated Coralbean	祛风湿清热药	Wind-Dampnessdispelling And Heatclearing Medicinal	4.833e-04	0.00434042	1	By_ingredient	0.335
SMHB00164	黑豆	Heidou	Sojae Semen Nigrum	Black bean	补阴药	Yin-Tonifying Medicinal	0.0336459	0.0753835	1	By_ingredient	0.3415
SMHB00183	槐角	Huaijiao	Sophorae Fructus	pod of Japanese pagodatree	凉血止血药	Blood-Cooling Hemostatic Medicinal	0.00365163	0.0319309	1	By_ingredient	0.3276
SMHB00185	黄精	Huangjing	Polygonati Rhizoma	Rhizome of Fragrant Solomonseal	补阴药	Yin-Tonifying Medicinal	0.00515542	0.0366581	1	By_ingredient	0.3596
SMHB00187	黄芪	Huangqi	Astragali Radix	root of Membranous Milkvetch	补气药	Qi Reinforcing Drugs	0.00703734	0.041619	1	By_ingredient	0.4808
SMHB00188	黄芩	Huangqin	Scutellariae Radix	root of Baikal skullcap	清热燥湿药	Heat-Clearing And Dampnessdrying Medicinal	0.0100687	0.0474436	1	By_ingredient	0.6175
SMHB00194	鸡血藤	Jixueteng	Spatholobi Caulis	Suberect Spatholobus Stem	活血祛瘀药	Blood Activating Stasis Removing Drugs	0.0959535	0.120753	1	By_ingredient	0.3304
SMHB00216	桔梗	Jiegeng	Platycodonis Radix	Platycodon Root	化痰药	Phlegresolving Medicine	0.0253818	0.0672724	1	By_ingredient	0.3863
SMHB00218	菊苣	Juju	Cichorii Herba Cichorii Radix	Chicory Herb	拔毒化腐生肌药	External Medicinal (Draw Out Toxin, Resolve Putridity)	0.00197069	0.0233139	1	By_ingredient	0.519
SMHB00230	苦杏仁	Kuxingren	Armeniacae Semen Amarum	bitter Apricot seed	止咳平喘药	Antitussive Antiasthmetics	0.0925974	0.11726	1	By_ingredient	0.1414
SMHB00232	昆布	Kunbu	Laminariae Thallus Eckloniae Thallus	Kelp or Tangle	化痰药	Phlegresolving Medicine	0.0110612	0.0496469	1	By_ingredient	0.3587
SMHB00237	雷公藤	Leigongteng	Radix Tripterygii Wilfordii	root of common htreewingnut	祛风湿散寒药	Wind-Dampnessdispelling And Colddispersing Medicinal	0.00696009	0.041403	1	By_ingredient	0.6053
SMHB00241	连翘	Lianqiao	Forsythiae Fructus	Weeping Forsythia Capsule	清热解毒药	Antipyretic Detoxicate Drugs	0.111466	0.136824	1	By_ingredient	0.208
SMHB00248	灵芝	Lingzhi	Ganoderma	Lucid Ganoderma	补气药	Qi Reinforcing Drugs	0.0200577	0.0612974	1	By_ingredient	0.5002
SMHB00255	芦根	Lugen	Phragmitis Rhizoma	Reed rhizome	清热泻火药	Fire Purging Drugs	0.0132405	0.0521346	1	By_ingredient	0.359
SMHB00260	罗汉果	Luohanguo	Siraitiae Fructus	Grosvenor Momordica Fruit	化痰药	Phlegresolving Medicine	0.11421	0.139681	1	By_ingredient	0.1614
SMHB00261	络石藤	Luoshiteng	Trachelospermi Caulis Et Folium	stem of Chinese starjasmine	祛风湿清热药	Wind-Dampnessdispelling And Heatclearing Medicinal	0.0367299	0.0582694	1	By_ingredient	0.2021
SMHB00266	马鞭草	Mabiancao	Verbenae Herba	European Verbe Herb	活血祛瘀药	Blood Activating Stasis Removing Drugs	0.00493782	0.0358445	1	By_ingredient	0.5054
SMHB00272	麦芽	Maiya	Hordei Fructus Germinatus	Germited Barley	消食药	Digestants	0.0160808	0.0567689	1	By_ingredient	0.3436
SMHB00277	没药	Moyao	Myrrha	Myrrh	活血祛瘀药	Blood Activating Stasis Removing Drugs	0.304515	0.334656	1	By_ingredient	0.784
SMHB00278	玫瑰花	Meiguihua	Rosae Rugosae Flos	Rose Flower	理气药	Qi Regulating Drugs	0.0830261	0.107332	1	By_ingredient	0.2047
SMHB00279	梅花	Meihua	Mume Flos	Plum Flower	理气药	Qi Regulating Drugs	0.0875417	0.112052	1	By_ingredient	0.1702
SMHB00283	墨旱莲	Mohanlian	Ecliptae Herba	all-grass of Yerbadetajo	补阴药	Yin-Tonifying Medicinal	0.00849321	0.0445421	1	By_ingredient	0.3269
SMHB00289	木蝴蝶	Muhudie	Oroxyli Semen	Indian Trumpetflower Seed	清热解毒药	Antipyretic Detoxicate Drugs	4.32134e-05	0.00178446	1	By_ingredient	0.7083
SMHB00293	木贼	Muzei	Equiseti Hiemalis Herba	Common Scouring Rush Herb	辛凉解表药	Pungent Cool Diaphoretics	0.057822	0.0808248	1	By_ingredient	0.153
SMHB00296	南鹤虱	Nanheshi	Carotae Fructus	Fructus Carotae	驱虫药	Antiparasitic Drugs	0.0537542	0.0765406	1	By_ingredient	0.1779
SMHB00298	南蛇藤果	nshetengguo	None	Celastri Orbiculati Fructus	安神药	Tranguilizing Medicinal	0.0130148	0.051746	1	By_ingredient	0.3705
SMHB00304	牛膝	Niuxi	Achyranthis Bidentatae Radix	root of Twotooth Achyranthes	活血祛瘀药	Blood Activating Stasis Removing Drugs	0.00532213	0.0373683	1	By_ingredient	0.5393
SMHB00306	女贞子	Nüzhenzi	Ligustri Lucidi Fructus	fruit of Glossy privet	补阴药	Yin-Tonifying Medicinal	0.195022	0.222444	1	By_ingredient	0.381
SMHB00311	平贝母	Pingbeimu	Fritillariae Ussuriensis Bulbus	Ussuri Fritillary Bulb	化痰药	Phlegresolving Medicine	0.00390698	0.032859	1	By_ingredient	0.3649
SMHB00314	千斤拔	Qianjinba	Radix Moghaniae Philippinensis	root of Philippine Flemingia	祛风湿筋骨药	Wind-Damp-Dispelling And Sinew-Bone-Strengthening Medicinal	0.0112713	0.0500184	1	By_ingredient	0.3379
SMHB00336	人参	Renshen	Ginseng Radix Et Rhizoma	Ginseng	补气药	Qi Reinforcing Drugs	0.0058002	0.0388319	1	By_ingredient	0.9222
SMHB00338	肉苁蓉	Roucongrong	Cistanches Herba	Desertliving Cistanche	补阳药	Yang Reinforcing Drugs	0.046157	0.068456	1	By_ingredient	0.3955
SMHB00339	肉豆蔻	Roudoukou	Myristicae Semen	Nutmeg	收涩药	Astringent Medicinal	0.0595605	0.0826652	1	By_ingredient	0.2109
SMHB00353	沙棘	Shaji	Hippophae Fructus	fruit of seabuckthorn	化痰药	Phlegresolving Medicine	0.173681	0.200545	1	By_ingredient	0.3342
SMHB00357	山豆根	Shandougen	Sophorae Tonkinensis Radix Et Rhizoma	Vietmese Sophora Root	清热解毒药	Antipyretic Detoxicate Drugs	0.0881047	0.112562	1	By_ingredient	0.1871
SMHB00364	射干	Shegan	Belamcandae Rhizoma	Blackberrylily Rhizome	清热解毒药	Antipyretic Detoxicate Drugs	0.0670597	0.0905647	1	By_ingredient	0.1905
SMHB00379	水飞蓟	Shuifeiji	Silybi Fructus	Silybum Marianum	清热解毒药	Antipyretic Detoxicate Drugs	0.0361379	0.0575949	1	By_ingredient	0.2112

Herb id	Chinese name	Pinyin name	Latin name	English name	Class in Chinese	Class in English	P_value	FDR(BH)	FDR(Bonferroni)	Relationship	Inferred evidence score
SMHB00388	太子参	Taizishen	Pseudostellariae Radix	Heterophylly Falsestarwort Root	补气药	Qi Reinforcing Drugs	0.0165775	0.0574553	1	By_ingredient	0.3238
SMHB00393	天葵子	Tiankuizi	Semiaquilegiae Radix	Muskroot-like Semiaquilegia Root	清热解毒药	Antipyretic Detoxicate Drugs	0.0514226	0.0741306	1	By_ingredient	0.1694
SMHB00397	天仙子	Tianxianzi	Hyoscyami Semen	Henbane Seed	止咳平喘药	Antitussive Antiasthmetics	0.0420431	0.0639691	1	By_ingredient	0.1885
SMHB00398	天竺黄	Tianzhuhuang	Bambusae Concretio Silicea	Chinese schinzostachyum Tabasheer	化痰药	Phlegresolving Medicine	0.0589813	0.0821102	1	By_ingredient	0.1784
SMHB00405	土茯苓	Tufuling	Smilacis Glabrae Rhizoma	Glabrous Greenbrier Rhizome	清热解毒药	Antipyretic Detoxicate Drugs	0.0155908	0.0561019	1	By_ingredient	0.3631
SMHB00412	委陵菜	Weilingcai	Potentiliae Chinensis Herba	Chinese Cinquefoil	清热解毒药	Antipyretic Detoxicate Drugs	0.0455703	0.0678477	1	By_ingredient	0.1514
SMHB00440	薤白	Xiebai	Allii Macrostemonis Bulbus	bulb of longstamen onion	理气药	Qi Regulating Drugs	0.0250831	0.0670191	1	By_ingredient	0.3541
SMHB00455	伊贝母	Yibeimu	Fritillariae Pallidiflorae Bulbus	Sinkiang Fritillary Bulb	化痰药	Phlegresolving Medicine	0.0476831	0.0889541	1	By_ingredient	0.2079
SMHB00460	银柴胡	Yincha	Stellariae Radix	Starwort Root	清虚热药	Asthenic Heat Dispelling Drugs	0.00689707	0.0412454	1	By_ingredient	0.354
SMHB00472	芫花	Yuanhua	Genkwa Flos	flower bud of Lilac Daphne	通水药	Drastic Purgatives	0.0343598	0.0557207	1	By_ingredient	0.1904
SMHB00481	浙贝母	Zhebeimu	Fritillariae Thunbergii Bulbus	bulb of Thunberg Fritillary	化痰药	Phlegresolving Medicine	0.00365163	0.0319309	1	By_ingredient	0.3826
SMHB00496	紫苏叶	Zisuye	Perillae Folium	None	辛温解表药	Pungent-Warm Exterior-Releasing Medicinal	0.023794	0.0654759	1	By_ingredient	0.5091
SMHB00499	葛根	Gegen	Puerariae Lobatae Radix	root of lobed kudzuvine	辛温解表药	Pungent-Warm Exterior-Releasing Medicinal	0.0109696	0.0278828	1	By_ingredient	0.1629
SMHB00532	片姜黄	Pianjianghuang	Wenyujin Rhizoma Concisum	None	活血化瘀药	Blood-Activating And Stasis-Resolving Medicinal	0.0476831	0.0889541	1	By_ingredient	0.2661
SMHB00577	红豆蔻	Hongdoukou	Galangae Fructus	None	温里药	Warming Interior Drugs	0.00886835	0.0452364	1	By_ingredient	0.3709
SMHB00593	龟甲	Guijia	Testudinis Carapax Et Plastrum	None	补虚药	Tonifying And Replenishing Medicinal	0.018728	0.0599011	1	By_ingredient	0.2381
SMHB00617	制草乌	Zh1caowu	Aconiti Kusnezoffii Radix Cocta	None	祛风湿药	Wind-Dampnessdispelling Medicinal	0.0386306	0.0800783	1	By_ingredient	0.1784
SMHB00622	厚朴花	Houpohua	Magnoliae Officinalis Flos	None	化湿药	Dampness Removing Drugs	0.0062811	0.0395886	1	By_ingredient	0.1711
SMHB00658	鹿茸	Lurong	Cervi Cornu Pantotrichum	None	补虚药	Tonifying And Replenishing Medicinal	0.0371137	0.0789024	1	By_ingredient	0.1476
SMHB00681	蜂房	Fengfang	Vespae Nidus	None	攻毒杀虫止痒药	Toxin-Attacking And Worm-Expelling And Itch-Relieving Medicinal	0.0476831	0.0889541	1	By_ingredient	0.1945

S2E. ICAM1 corresponding traditional Chinese medicines

Columns are reproduced from the source workbook. P_value and FDR columns report the SymMap statistical output; relationship and evidence score describe inferred gene-herb evidence.

Herb id	Chinese name	Pinyin name	Latin name	English name	Class in Chinese	Class in English	P_value	FDR(BH)	FDR(Bonferroni)	Relationship	Inferred evidence score
SMHB00001	矮地茶	Aidicha	Ardisiae Japonicae Herba	Japanese Ardisia Herb	止咳平喘药	Antitussive Antiasthmetics	0.00704419	0.0225739	1	By_ingredient	0.7205
SMHB00002	艾叶	Aiye	Artemisiae Argyi Folium	leaf of Argy Wormwood	温经止血药	Channel-Warming Hemostatic	0.00865348	0.0448525	1	By_ingredient	0.5413
SMHB00003	安息香	Anxixiang	Benzoinum	Benzoin	开窍药	Resuscitative Stimulant; Resuscitative Medicinal	0.13146	0.157272	1	By_ingredient	0.3226
SMHB00004	八角茴香	Bajiaohuixiang	Anisi Stellati Fructus	Chinese Star Anise	温里药	Warming Interior Drugs	1.683e-04	0.00208973	1	By_ingredient	0.4393
SMHB00005	八角莲	Bajiaolian	None	Dysosmae Verspiellis Rhixoma Et Radix	清热解毒药	Antipyretic Detoxicate Drugs	0.0113245	0.0283993	1	By_ingredient	0.319
SMHB00006	巴豆	Badou	Crotonis Fructus	Croton Fruit	攻下药	Offensive Purgative Medicinal	0.00353449	0.0315771	1	By_ingredient	0.5201
SMHB00007	巴戟天	Bajitian	Morindae Officinalis Radix	Morindae Officilis Radix	补阳药	Yang Reinforcing Drugs	0.0204262	0.0618352	1	By_ingredient	0.5174
SMHB00008	菝葜	Baqia	Smilacis Chinae Rhizoma	Smilax Chi	祛风湿散寒药	Wind-Dampnessdispelling And Colddispersing Medicinal	4.155e-04	0.00883826	1	By_ingredient	0.6998
SMHB00009	白扁豆	Baibiandou	Lablab Semen Album	White Hyacinth Bean	补气药	Qi Reinforcing Drugs	1.03E-05	5.953e-04	1	By_ingredient	0.8204
SMHB00010	白附子	Baifuzi	Typhonii Rhizoma	rhizome of Giant Typhonium	化痰药	Phlegresolving Medicine	0.00196552	0.0233139	1	By_ingredient	0.6608
SMHB00011	白果	Baiguo	Ginkgo Semen	Ginkgo seed	止咳平喘药	Antitussive Antiasthmetics	5.34E-05	0.00207715	1	By_ingredient	0.8175
SMHB00012	白花菜子	Baihuacaizi	None	Semen Cleomis	祛风湿散寒药	Wind-Dampnessdispelling And Colddispersing Medicinal	0.0639142	0.0871221	1	By_ingredient	0.1664
SMHB00013	白花蛇舌草	Baihuasheshecao	Herba Hedyotis Diffusae	all - grass of Spreading Hedyotis	清热解毒药	Antipyretic Detoxicate Drugs	4.12E-07	4.43E-05	0.170562	By_ingredient	1.1568
SMHB00014	白及	Baiji	Bletillae Rhizoma	Bletilla Striata (Thunb.Ex A.Murray)Rchb.F.	收敛止血药	Astringent Hemostatic Medicinal	0.0174339	0.0582409	1	By_ingredient	0.3928
SMHB00015	白蔹	Bailian	Ampelopsis Radix	root of Japanese Ampelopsis	清热解毒药	Antipyretic Detoxicate Drugs	6.679e-04	0.00541206	1	By_ingredient	0.6468
SMHB00016	白毛藤	Baimaoteng	None	Herba Solani Lyrati	清热解毒药	Antipyretic Detoxicate Drugs	0.0299906	0.0718632	1	By_ingredient	0.3752
SMHB00020	白屈菜	Baiqucai	Chelidonii Herba	Chelidonii Herba	止咳平喘药	Antitussive Antiasthmetics	0.0366435	0.0782226	1	By_ingredient	0.3315
SMHB00021	白芍	Baishao	Paeoniae Radix Alba	White peony root	补血药	Blood-Tonifying Medicinal	1.24E-05	6.882e-04	1	By_ingredient	1.3531
SMHB00023	白头翁	Baitouweng	Pulsatillae Radix	Root of Chinese Pulsatilla	清热解毒药	Antipyretic Detoxicate Drugs	0.012032	0.0293794	1	By_ingredient	0.3084
SMHB00025	白鲜皮	Baixianpi	Dictamni Cortex	Dictamni Cortex	清热燥湿药	Heat-Clearing And Dampnessdrying Medicinal	7.926e-04	0.00614104	1	By_ingredient	0.4429
SMHB00026	白药子	Baiyaozi	None	Radix Stephaniae Cepharanthae.	清热解毒药	Antipyretic Detoxicate Drugs	0.0353949	0.0768127	1	By_ingredient	0.3389
SMHB00027	白芷	Baizhi	Angelicae Dahuricae Radix	Dahurian Angelica Root	辛温解表药	Pungent-Warm Exterior-Releasing Medicinal	0.0217178	0.0635889	1	By_ingredient	0.6357
SMHB00031	败酱草	Baijiangcao	Herba Patriniae	All - grass of Dahurian Patrinia	清热解毒药	Antipyretic Detoxicate Drugs	1.26E-11	3.77E-09	1.11E-06	By_ingredient	1.1922
SMHB00033	板蓝根	Banlangen	Isatidis Radix	Root of Dyers Woad.	清热解毒药	Antipyretic Detoxicate Drugs	0.00875857	0.0449348	1	By_ingredient	0.6192
SMHB00034	半边莲	Banbianlian	Lobeliae Chinensis Herba	Chinese Lobelia Herb	清热解毒药	Antipyretic Detoxicate Drugs	5.68E-07	3.15E-05	0.0501998	By_ingredient	0.7413
SMHB00035	半夏	Banxia	Pinelliae Rhizoma	tuber of Pinellia	化痰药	Phlegresolving Medicine	0.215119	0.243063	1	By_ingredient	0.141
SMHB00036	半枝莲	Banzhilian	Scutellariae Barbatae Herba	all - grass of Barbed Skullcap	清热解毒药	Antipyretic Detoxicate Drugs	1.94E-06	1.589e-04	0.803413	By_ingredient	1.086
SMHB00037	薄荷	Bohe	Menthae Haplocalycis Herba	Mentha, Peppermint	辛凉解表药	Pungent Cool Diaphoretics	4.70E-05	0.00190128	1	By_ingredient	1.2164
SMHB00038	北豆根	Beidougen	Menispermi Rhizoma	Chinese Cinquefoil	清热解毒药	Antipyretic Detoxicate Drugs	0.00692141	0.0224481	1	By_ingredient	0.371
SMHB00039	北刘寄奴	Beiliujinu	Siphonostegiae Herba	Siphonostegiae Herba	活血祛瘀药	Blood Activating Stasis Removing Drugs	0.00454452	0.0182036	1	By_ingredient	0.256
SMHB00040	北沙参	Beishashen	Glehniae Radix	root of Coastal Glehnia	补阴药	Yin-Tonifying Medicinal	0.178632	0.205652	1	By_ingredient	0.1371
SMHB00041	五味子	Wuweizi	Schisandrae Chinensis Fructus	Schisandrae Chinensis Fructus	收涩药	Astringent Medicinal	0.21786	0.245878	1	By_ingredient	0.1458
SMHB00042	荜茇	Bibo	Piperis Longi Fructus	Piperis Longi Fructus	温里药	Warming Interior Drugs	0.190031	0.217424	1	By_ingredient	0.1838
SMHB00043	荜澄茄	Bichengqie	Litseae Fructus	Litseae Fructus	温里药	Warming Interior Drugs	0.0203052	0.0615907	1	By_ingredient	0.3337
SMHB00044	蓖麻子	Bimazi	Ricini Semen	Castor Seed	拔毒化腐生肌药	External Medicinal (Draw Out Toxin, Resolve Putridity)	0.0975153	0.122301	1	By_ingredient	0.1527
SMHB00045	萹蓄	Bianxu	Polygoni Avicularis Herba	all-grass of common knotgrass	利水渗湿药	Diuretic Dampness Excreting Drugs	1.284e-04	0.00393951	1	By_ingredient	0.8024
SMHB00046	扁豆花	Biandouhua	Flos Dolichoris	flower of Hyacinth Dolichos	化湿药	Dampness Removing Drugs	3.74E-05	0.0016072	1	By_ingredient	0.4746
SMHB00048	冰片	Bingpian	Borneolum Syntheticum	Borneol	开窍药	Resuscitative Stimulant; Resuscitative Medicinal	0.0249534	0.0669307	1	By_ingredient	0.5422
SMHB00049	布渣叶	Buzhaye	Microctis Folium	Microctis Folium	拔毒化腐生肌药	External Medicinal (Draw Out Toxin, Resolve Putridity)	5.23E-05	9.321e-04	1	By_ingredient	0.4302
SMHB00050	蚕沙	Cansha	None	Bombyx Mori L.	祛风湿散寒药	Wind-Dampnessdispelling And Colddispersing Medicinal	0.260467	0.289622	1	By_ingredient	0.1434
SMHB00051	苍耳子	Cang'erzi	Xanthii Fructus	fruit of Siberian Cockleblur	辛温解表药	Pungent-Warm Exterior-Releasing Medicinal	0.0220257	0.0639403	1	By_ingredient	0.4816
SMHB00054	草果	Caoguo	Tsaoko Fructus	Tsaoko	化湿药	Dampness Removing Drugs	0.0178995	0.0585784	1	By_ingredient	0.3181
SMHB00057	侧柏叶	Cebaiye	Platycladi Cacumen	leafy twigs of Oriental Arborvitae	凉血止血药	Blood-Cooling Hemostatic Medicinal	0.00103168	0.00741001	1	By_ingredient	0.5843
SMHB00058	柴胡	Chaihu	Bupleuri Radix	root of Chinese Thorowax	辛凉解表药	Pungent Cool Diaphoretics	0.00857556	0.0433803	1	By_ingredient	1.0166
SMHB00060	常山	Changshan	Dichroae Radix	root of antifebrile Dichroa	涌吐药	Emetic Medicinal	0.0360171	0.0774881	1	By_ingredient	0.2721
SMHB00061	车前草	Cheqiancao	Plantaginis Herba	all-grass of Rippleseed plantain	利水渗湿药	Diuretic Dampness Excreting Drugs	1.065e-04	0.00343292	1	By_ingredient	1.071
SMHB00062	车前子	Cheqianzi	Plantaginis Semen	seed of Asiatic pantain	利水渗湿药	Diuretic Dampness Excreting Drugs	1.08E-06	9.76E-05	0.304027	By_ingredient	0.8891
SMHB00063	沉香	Chenxiang	Aquilariae Lignum Resinatum	Oily wood of Agalloch Eaglewood	理气药	Qi Regulating Drugs	0.277124	0.306671	1	By_ingredient	0.3235
SMHB00064	陈皮	Chenpi	Citri Reticulatae Pericarpium	Dried Tangerine Peel	理气药	Qi Regulating Drugs	0.012223	0.0506489	1	By_ingredient	0.4361
SMHB00065	赤芍	Chishao	Paeoniae Radix Rubra	Red Peony root	清热凉血药	Heat Clearing Blood Cooling Drugs	1.043e-04	0.00338216	1	By_ingredient	1.1117
SMHB00066	赤小豆	Chixiaodou	Vignae Semen	Rice Bean	利水渗湿药	Diuretic Dampness Excreting Drugs	0.00792313	0.0435365	1	By_ingredient	0.3484
SMHB00068	臭梧桐	Chouwutong	Folium Clerodentri Trichotomi	Glorybower leaf, Clerodendron leaf	祛风湿清热药	Wind-Dampnessdispelling And Heatclearing Medicinal	0.0622842	0.0855707	1	By_ingredient	0.1715
SMHB00069	楮实子	Chushizi	Broussonetiae Fructus	Broussonetiae Fructus	补阴药	Yin-Tonifying Medicinal	0.00192905	0.0114055	1	By_ingredient	0.6246
SMHB00070	川贝母	Chuanbeimu	Fritillariae Cirrhosae Bulbus	Bulb of Tendrilleaf Fritillary	化痰药	Phlegresolving Medicine	0.00144254	0.0135773	1	By_ingredient	0.6635
SMHB00072	川楝子	Chuanlianzi	Toosendan Fructus	Szechwan Chiberry Fruit	理气药	Qi Regulating Drugs	0.205454	0.233267	1	By_ingredient	0.4369
SMHB00073	川木通	Chuanmutong	Clematidis Armandii Caulis	Armand Clematis Stem	利水渗湿药	Diuretic Dampness Excreting Drugs	0.00900121	0.0455735	1	By_ingredient	0.8031
SMHB00074	川牛膝	Chuanniuxi	Cyathulae Radix	root of Medicil cyathula	活血祛瘀药	Blood Activating Stasis Removing Drugs	1.28E-06	5.91E-05	0.113126	By_ingredient	0.6905
SMHB00075	川射干	Chuanshegan	Iridis Tectori Rhizoma	Iridis Tectori Rhizoma	清热解毒药	Antipyretic Detoxicate Drugs	0.277124	0.306671	1	By_ingredient	0.3696
SMHB00076	川乌	Chuanwu	Aconiti Radix	root of Common Monkshood	祛风湿散寒药	Wind-Dampnessdispelling And Colddispersing Medicinal	0.311775	0.342116	1	By_ingredient	0.1549
SMHB00077	川芎	Chuanxiong	Chuanxiong Rhizoma	Chuanxiong rhizome, Szechuan lovage root	活血祛瘀药	Blood Activating Stasis Removing Drugs	0.298359	0.328356	1	By_ingredient	0.3368
SMHB00079	穿心莲	Chuanxinlian	Andrographis Herba	all-grass of Common Androgrphis	清热解毒药	Antipyretic Detoxicate Drugs	8.86E-05	0.00300605	1	By_ingredient	0.8243
SMHB00080	垂盆草	Chuipencao	Sedi Herba	all - grass of Stringy stonecrop	清热解毒药	Antipyretic Detoxicate Drugs	0.0137563	0.0318317	1	By_ingredient	0.3007
SMHB00081	椿皮	Chunpi	Ailanthi Cortex	Ailanthus bark, Tree of heaven bark	清热燥湿药	Heat-Clearing And Dampnessdrying Medicinal	1.98E-05	4.603e-04	1	By_ingredient	0.6324
SMHB00082	葱白	Congbai	Bulbus Allii Fistulosi	Allium bulb, Wild scallion, Chinese green onion	辛温解表药	Pungent-Warm Exterior-Releasing Medicinal	4.350e-04	0.00853342	1	By_ingredient	0.8063
SMHB00083	大风子	Dafengzi	None	Hydnocarpi Anthelminticae Semen	解毒杀虫燥湿止痒药	Medicinal For Detoxification、Parasiticide、Drying Dampness And Relieving Itching	9.76E-07	4.81E-05	0.0862589	By_ingredient	0.6177
SMHB00084	大腹皮	Dafupi	Arecae Pericarpium	Areca Catechu L.	理气药	Qi Regulating Drugs	0.00760319	0.0335308	1	By_ingredient	0.4352
SMHB00085	大黄	Dahuang	Rhei Radix Et Rhizoma	root and rhziome of Sorrel Rhubarb	攻下药	Offensive Purgative Medicinal	4.205e-04	0.00891688	1	By_ingredient	0.8604
SMHB00086	大蓟	Daji	Cirsii Japonici Herba	all-grass of Thistle	凉血止血药	Blood-Cooling Hemostatic Medicinal	3.477e-04	0.00778821	1	By_ingredient	0.9245
SMHB00088	大蒜	Dasuan	Allii Sativi Bulbus	Garlic	解毒杀虫燥湿止痒药	Medicinal For Detoxification、Parasiticide、Drying Dampness And Relieving Itching	7.85E-05	0.00139641	1	By_ingredient	0.7285
SMHB00089	大血藤	Daxueteng	Sargentodoxae Caulis	Stem of Sargentgloryvine	清热解毒药	Antipyretic Detoxicate Drugs	0.0169737	0.0580082	1	By_ingredient	0.3244
SMHB00090	大枣	Dazao	Jujubae Fructus	Jujube Chinese date	补气药	Qi Reinforcing Drugs	0.0025615	0.0135773	1	By_ingredient	0.7809
SMHB00091	大皂角	Dazaojiao	Gleditsiae Sinensis Fructus	Gleditsiae Fructus	化痰药	Phlegresolving Medicine	5.200e-04	0.010311	1	By_ingredient	0.5992
SMHB00092	玳玳花	Daidaihua	None	Citrus Aurantium	理气药	Qi Regulating Drugs	2.481e-04	0.00624188	1	By_ingredient	0.7457
SMHB00093	丹参	Danshen	Salviae Miltiorrhizae Radix Et Rhizoma	root of Ligulilobe sage	活血祛瘀药	Blood Activating Stasis Removing Drugs	8.23E-08	7.01E-06	0.00727367	By_ingredient	1.1271
SMHB00094	淡豆豉	Dandouchi	Sojae Semen Praeparatum	Fermented Soybean	辛凉解表药	Pungent Cool Diaphoretics	1.89E-08	3.19E-06	0.00783414	By_ingredient	0.9922

Herb id	Chinese name	Pinyin name	Latin name	English name	Class in Chinese	Class in English	P_value	FDR(BH)	FDR(Bonferroni)	Relationship	Inferred evidence score
SMHB00096	党参	Dangshen	Codonopsis Radix	root Pilose Asiabell	补气药	Qi Reinforcing Drugs	0.0092966	0.0462579	1	By_ingredient	0.5715
SMHB00097	刀豆	Daodou	Cana v Aliae Semen	Jack Bean	理气药	Qi Regulating Drugs	0.00579063	0.0388093	1	By_ingredient	0.3183
SMHB00098	倒扣草	Daokoucao	None	Achyranthis Asperae Herba	活血祛瘀药	Blood Activating Stasis Removing Drugs	3.076e-04	0.00319396	1	By_ingredient	0.4817
SMHB00099	灯心草	Dengxincao	Junci Medulla	pith of Common Rush	利水渗湿药	Diuretic Dampness Excreting Drugs	0.00300385	0.0294404	1	By_ingredient	0.4684
SMHB00100	地耳草	Diercao	Herba Hyperici Japonici	all - grass of Japanese St. Johnswort	利水渗湿药	Diuretic Dampness Excreting Drugs	0.0030399	0.0296147	1	By_ingredient	0.7053
SMHB00102	地肤子	Difuzi	Kochiae Fructus	fruit of Belvedere	利水渗湿药	Diuretic Dampness Excreting Drugs	0.0160691	0.0567353	1	By_ingredient	0.5177
SMHB00103	地骨皮	Digupi	Lycii Cortex	root - bark of Chinese Wolfberry	清虚热药	Asthenic Heat Dispelling Drugs	0.103781	0.128837	1	By_ingredient	0.1376
SMHB00104	地锦草	Dijincao	Euphorbiae Humifusae Herba	all - grass of Humifuse Euphorbia	清热解毒药	Antipyretic Detoxicate Drugs	5.63E-05	0.00216219	1	By_ingredient	0.6605
SMHB00105	地榆	Diyu	Sanguisorbae Radix	Root of Garden Burnet	凉血止血药	Blood-Cooling Hemostatic Medicinal	2.026e-04	0.00239019	1	By_ingredient	0.6232
SMHB00107	丁香	Dingxiang	Caryophylli Flos	Clove	温里药	Warming Interior Drugs	0.00146863	0.00952975	1	By_ingredient	0.498
SMHB00108	冬虫夏草	Dongchongxiacao	Cordyceps	Chinese Caterpillar Fungus	补阳药	Yang Reinforcing Drugs	0.194265	0.221712	1	By_ingredient	0.1676
SMHB00109	冬瓜皮	Dongguapi	Benincasae Exocarpium	Chinese Waxgourd Peel	利水渗湿药	Diuretic Dampness Excreting Drugs	0.0248401	0.0668944	1	By_ingredient	0.4951
SMHB00110	冬瓜子	Dongguazi	None	Benincasae Semen	化痰药	Phlegresolving Medicine	1.068e-04	0.00344148	1	By_ingredient	0.7647
SMHB00111	冬葵果	Dongkuiguo	Malvae Fructus	Cluster Mallow Fruit	拔毒化腐生肌药	External Medicinal (Draw Out Toxin, Resolve Putridity)	0.00344235	0.0313804	1	By_ingredient	0.4971
SMHB00112	豆蔻	Doukou	Amomi Fructus Rotundus	Round Cardamon Fruit	化湿药	Dampness Removing Drugs	1.380e-04	0.00183049	1	By_ingredient	0.6316
SMHB00113	独活	Duhuo	Angelicae Pubescentis Radix	Doubleteeth Pubescent Angelica Root	祛风湿散寒药	Wind-Dampnessdispelling And Colddispersing Medicinal	0.167078	0.19368	1	By_ingredient	0.3444
SMHB00114	杜仲	Duzhong	Eucommiae Cortex	Eucommia bark	补阳药	Yang Reinforcing Drugs	8.07E-06	2.401e-04	0.713227	By_ingredient	0.7526
SMHB00115	杜仲叶	Duzhongye	Eucommiae Folium	Eucommiae Folium	拔毒化腐生肌药	External Medicinal (Draw Out Toxin, Resolve Putridity)	0.0271405	0.0476951	1	By_ingredient	0.3323
SMHB00116	断血流	Duanxueliu	Clinopodii Herba	Clinopodium Herb	凉血止血药	Blood-Cooling Hemostatic Medicinal	4.350e-04	0.00701971	1	By_ingredient	0.5887
SMHB00118	鹅不食草	Ebushicao	Centipedae Herba	Small Centipeda Herb	辛温解表药	Pungent-Warm Exterior-Releasing Medicinal	7.61E-05	0.00268793	1	By_ingredient	0.8132
SMHB00119	儿茶	Ercha	Catechu	Cutch, Black Catechu	活血祛瘀药	Blood Activating Stasis Removing Drugs	0.00118657	0.0148459	1	By_ingredient	0.4426
SMHB00120	番泻叶	Fanxieye	Sennae Folium	Sen leaf	攻下药	Offensive Purgative Medicinal	0.0404896	0.0822379	1	By_ingredient	0.3179
SMHB00121	翻白草	Fanbaicao	Potentillae Discoloris Herba	all-grass of discolor cinquefoil	清热解毒药	Antipyretic Detoxicate Drugs	5.01E-09	9.93E-07	0.00207354	By_ingredient	1.3013
SMHB00123	防己	Fangji	Stephaniae Tetrandrae Radix	Root of Fourstamen Stephania	祛风湿清热药	Wind-Dampnessdispelling And Heatclearing Medicinal	0.135992	0.161911	1	By_ingredient	0.177
SMHB00124	粉萆薢	Fenbixie	Dioscoreae Hypoglaucae Rhizoma	Dioscoreae Hypoglaucae Rhizoma	利水渗湿药	Diuretic Dampness Excreting Drugs	0.0114862	0.0500184	1	By_ingredient	0.3232
SMHB00125	凤尾草	Fengweicao	Herba Pteridis Multifidae	all - grass of Chinese Brake	清热解毒药	Antipyretic Detoxicate Drugs	2.312e-04	0.00261666	1	By_ingredient	0.4343
SMHB00126	凤仙透骨草	Fengxiantougucao	Herba Speranskiae Tuberculatae	all-grass of Tubercultae speranskia	祛风湿散寒药	Wind-Dampnessdispelling And Colddispersing Medicinal	0.00149151	0.00958548	1	By_ingredient	0.306
SMHB00127	凤眼草	Fengyancao	None	Ailanthi Altissimae Fructus	清热解毒药	Antipyretic Detoxicate Drugs	1.90E-09	3.09E-07	1.679e-04	By_ingredient	0.7789
SMHB00130	浮萍	Fuping	Spirodelae Herba	all-grass of Common Ducksmeat	辛凉解表药	Pungent Cool Diaphoretics	1.765e-04	0.00216907	1	By_ingredient	0.4485
SMHB00132	覆盆子	Fupenzi	Rubi Fructus	Rubi Fructus	收涩药	Astringent Medicinal	1.91E-06	8.05E-05	0.168806	By_ingredient	1.1045
SMHB00133	甘草	Gancao	Glycyrrhizae Radix Et Rhizoma	Root of Ural Licorice	补气药	Qi Reinforcing Drugs	1.197e-04	0.00165595	1	By_ingredient	1.1171
SMHB00134	甘松	Gansong	Nardostachyos Radix Et Rhizoma	rdostachys Root	理气药	Qi Regulating Drugs	2.412e-04	0.00269347	1	By_ingredient	0.8104
SMHB00135	甘遂	Gansui	Kansui Radix	root of Gansui	通水药	Drastic Purgatives	0.0329491	0.0747138	1	By_ingredient	0.3669
SMHB00136	干姜	Ganjiang	Zingiberis Rhizoma	Dried Ginger	温里药	Warming Interior Drugs	0.0496735	0.0866996	1	By_ingredient	0.4277
SMHB00137	高良姜	Gaoliangjiang	Alpiniae Officinarum Rhizoma	Alpiniae Officirum Rhizome	温里药	Warming Interior Drugs	6.49E-07	6.35E-05	0.268358	By_ingredient	1.0682
SMHB00139	葛花	Gehua	Flos Puerariae Lobatae	flower bud of lobed kudzuvine	辛凉解表药	Pungent Cool Diaphoretics	2.92E-06	2.211e-04	1	By_ingredient	1.0842
SMHB00140	功劳木	Gonglaomu	Mahoniae Caulis	Stem of Leatherleaf Mahonia	清热解毒药	Antipyretic Detoxicate Drugs	0.0353949	0.0768127	1	By_ingredient	0.5014
SMHB00141	钩藤	Gouteng	Uncariae Ramulus Cumuncis	Gambir Plant	平肝熄风药	Liver-Pacifying Wind-Extinguishing Medicinal	2.06E-05	4.744e-04	1	By_ingredient	0.8091
SMHB00142	枸骨叶	Gouguye	Ilicis Cornutae Folium	Folium Ilicis Cornutae	清虚热药	Asthenic Heat Dispelling Drugs	7.52E-09	1.41E-06	0.00310815	By_ingredient	1.385
SMHB00143	枸杞子	Gouqizi	Lycii Fructus	fruit of Chinese Wolfberry	补阴药	Yin-Tonifying Medicinal	0.0125753	0.0513902	1	By_ingredient	0.6416
SMHB00144	谷精草	Gujingcao	Eriocauli Flos	Pipewort Flower	清热泻火药	Fire Purging Drugs	0.00229633	0.0127743	1	By_ingredient	0.2838
SMHB00145	骨碎补	Gusuibu	Drynariae Rhizoma	rhizome of Heartleaf Dryria	补阳药	Yang Reinforcing Drugs	0.00453951	0.0226325	1	By_ingredient	0.4937
SMHB00147	瓜蒌	Gualou	Trichosanthis Fructus	Trichosanthes Kirilowii Maxim	化痰药	Phlegresolving Medicine	0.00731137	0.0269533	1	By_ingredient	0.4697
SMHB00148	关黄柏	Guanhuangbo	Phellodendri Amurensis Cortex	Phellodendri Amurensis Cortex	清热燥湿药	Heat-Clearing And Dampnessdrying Medicinal	8.147e-04	0.00627668	1	By_ingredient	0.4591
SMHB00149	贯叶金丝桃	Guanyejinsitao	Hyperici Perforati Herba	Herbahypericiperforati	收敛止血药	Astringent Hemostatic Medicinal	9.37E-06	5.497e-04	1	By_ingredient	0.7107
SMHB00150	绵马贯众	Mianmaguanzhong	Dryopteridis Crassirhizomatis Rhizoma	root of Chinese Angelica	清热解毒药	Antipyretic Detoxicate Drugs	0.0063734	0.0213584	1	By_ingredient	0.315
SMHB00151	广藿香	Guanghuoxiang	Pogostemonis Herba	Pogostemon Cablin (Blanco) Benth.	化湿药	Dampness Removing Drugs	6.31E-05	0.00106876	1	By_ingredient	0.629
SMHB00152	广枣	Guangzao	Choerospondiatis Fructus	fruit of Axillary choerospondias	活血祛瘀药	Blood Activating Stasis Removing Drugs	7.15E-05	0.0025666	1	By_ingredient	0.7681
SMHB00153	鬼箭羽	Guijianyu	Ramulus Euonymi	twig of Winged Euonymus	活血祛瘀药	Blood Activating Stasis Removing Drugs	5.21E-05	9.300e-04	1	By_ingredient	1.0123
SMHB00154	鬼针草	Guizhencao	Herba Bidentis	all-grass of Spanishneedles	清热解毒药	Antipyretic Detoxicate Drugs	0.00143671	0.0195475	1	By_ingredient	0.4526
SMHB00155	桂枝	Guizi	Cinnamomi Ramulus	Cassia Twig	辛温解表药	Pungent-Warm Exterior-Releasing Medicinal	1.54E-06	1.310e-04	0.637097	By_ingredient	1.3681
SMHB00157	海金沙	Haijinsha	Lygodii Spora	spore of Japanese climbing Fern	利水渗湿药	Diuretic Dampness Excreting Drugs	0.00550661	0.0197561	1	By_ingredient	0.319
SMHB00158	海桐皮	Haitongpi	Cortex Erythrie	bark of Oriental Variegated Coralbean	祛风湿清热药	Wind-Dampnessdispelling And Heatclearing Medicinal	4.400e-04	0.00918083	1	By_ingredient	0.5042
SMHB00159	海藻	Haizao	Sargassum	seaweed	化痰药	Phlegresolving Medicine	0.0687876	0.0924027	1	By_ingredient	0.1491
SMHB00161	荷叶	Heye	Nelumbinis Folium	Lotus leaf	清热解毒药	Antipyretic Detoxicate Drugs	4.05E-05	0.00170611	1	By_ingredient	0.7198
SMHB00162	核桃仁	Hetaoren	Juglandis Semen	English Walnut Seed	补阳药	Yang Reinforcing Drugs	0.00570138	0.0385322	1	By_ingredient	0.4776
SMHB00163	鹤虱	Heshi	Carpesii Fructus	Common Carpesium Fruit	驱虫药	Antiparasitic Drugs	0.0223279	0.0420173	1	By_ingredient	0.1516
SMHB00164	黑豆	Heidou	Sojae Semen Nigrum	Black bean	补阴药	Yin-Tonifying Medicinal	1.763e-04	0.00257987	1	By_ingredient	0.7698
SMHB00165	黑老虎	Heilaohu	None	Kadsurae Coccineae Radix Et Caulis	理气药	Qi Regulating Drugs	0.00567527	0.0201754	1	By_ingredient	0.3269
SMHB00168	红花	Honghua	Carthami Flos	Safflower	活血祛瘀药	Blood Activating Stasis Removing Drugs	3.226e-04	0.00330073	1	By_ingredient	0.531
SMHB00169	红芪	Hongqi	Hedysari Radix	Hedysari Radix	补气药	Qi Reinforcing Drugs	0.00242602	0.0132035	1	By_ingredient	0.418
SMHB00170	猴枣	Houzao	None	Macacae Calcalus Gasteris	化痰药	Phlegresolving Medicine	0.00247548	0.0263583	1	By_ingredient	0.3215
SMHB00172	胡黄连	Huhuangl1an	Picrorhizae Rhizoma	Picrorhizae Rhizoma	清虚热药	Asthenic Heat Dispelling Drugs	0.0195239	0.0386412	1	By_ingredient	0.2988
SMHB00173	胡椒	Hujiao	Piperis Fructus	Pepper Fruit	温里药	Warming Interior Drugs	0.0132207	0.038405	1	By_ingredient	0.537
SMHB00174	胡芦巴	Huluba	Trigonellae Semen	Trigonellae Semen	补阳药	Yang Reinforcing Drugs	1.42E-06	6.37E-05	0.1255	By_ingredient	0.8701
SMHB00175	槲寄生	Hujisheng	Visci Herba	Colored Mistletoe Herb	祛风湿筋骨药	Wind-Damp-Dispelling And Sinew-Bone-Strengthening Medicinal	0.011794	0.0289767	1	By_ingredient	0.4957
SMHB00176	虎耳草	Huercao	Herba Saxifragae	stem or leaf of Fung Waxplant	清热解毒药	Antipyretic Detoxicate Drugs	0.0800634	0.104143	1	By_ingredient	0.1289
SMHB00177	虎杖	Huzhang	Polygoni Cuspidati Rhizoma Et Radix	rhizome of Gaint Knotweed	利水渗湿药	Diuretic Dampness Excreting Drugs	6.76E-06	2.104e-04	0.597449	By_ingredient	1.1341
SMHB00178	花椒	Huajiao	Zanthoxyli Pericarpium	Pricklyash Peel	温里药	Warming Interior Drugs	0.0140109	0.0321924	1	By_ingredient	0.2901
SMHB00179	花生衣	Huashengyi	Testa Arachidis Hypogaeae	Semino-dermis Arachidis	收敛止血药	Astringent Hemostatic Medicinal	4.49E-12	1.83E-09	1.86E-06	By_ingredient	0.9914
SMHB00180	华山参	Huashanshen	Physochlainae Radix	Physochlaie Radix	止咳平喘药	Antitussive Antiasthmetics	0.0622842	0.0855707	1	By_ingredient	0.1433
SMHB00181	化橘红	Huajuhong	Citri Grandis Exocarpium	Pummelo Peel	理气药	Qi Regulating Drugs	0.162704	0.189233	1	By_ingredient	0.1339
SMHB00182	槐花	Huaihua	Sophorae Flos	flower bud of Japanese pagodatree	凉血止血药	Blood-Cooling Hemostatic Medicinal	2.367e-04	0.00601511	1	By_ingredient	0.8528
SMHB00183	槐角	Huaijiao	Sophorae Fructus	pod of Japanese pagodatree	凉血止血药	Blood-Cooling Hemostatic Medicinal	1.28E-06	5.91E-05	0.113126	By_ingredient	0.6288
SMHB00184	黄柏	Huangbo	Phellodendri Chinensis Cortex	Phellodendron bark	清热燥湿药	Heat-Clearing And Dampnessdrying Medicinal	0.00251583	0.0134533	1	By_ingredient	0.5958
SMHB00185	黄精	Huangjing	Polygonati Rhizoma	Rhizome of Fragrant Solomonseal	补阴药	Yin-Tonifying Medicinal	0.0160691	0.0567353	1	By_ingredient	0.2821
SMHB00186	黄连	Huanglian	Coptidis Rhizoma	rhizome of Chinese Goldthread	清热燥湿药	Heat-Clearing And Dampnessdrying Medicinal	0.103781	0.128837	1	By_ingredient	0.148
SMHB00187	黄芪	Huangqi	Astragali Radix	root of Membranous Milkvetch	补气药	Qi Reinforcing Drugs	7.217e-04	0.00755966	1	By_ingredient	0.8182
SMHB00188	黄芩	Huangqin	Scutellariae Radix	root of Baikal skullcap	清热燥湿药	Heat-Clearing And Dampnessdrying Medicinal	0.00875857	0.0449348	1	By_ingredient	0.6541

Herb id	Chinese name	Pinyin name	Latin name	English name	Class in Chinese	Class in English	P_value	FDR(BH)	FDR(Bonferroni)	Relationship	Inferred evidence score
SMHB00189	黄药子	Huangyaozi	Rhizoma Dioscoreae Bulbiferae	rhizome of Airpotato Yam	化痰药	Phlegresolving Medicine	0.00223457	0.0226325	1	By_ingredient	0.5127
SMHB00190	火麻仁	Huomaren	Cannabis Fructus	Hemp Seed	润下药	Laxatives	2.20E-07	2.65E-05	0.0910734	By_ingredient	1.4258
SMHB00191	鸡骨草	Jigucao	Abri Herba	Canton Love-pea Vine	利水渗湿药	Diuretic Dampness Excreting Drugs	0.00380906	0.0166498	1	By_ingredient	0.5276
SMHB00192	鸡冠花	Jiguanhua	Celosiae Cristatae Flos	Cockscomb flower	收敛止血药	Astringent Hemostatic Medicinal	6.010e-04	0.00504669	1	By_ingredient	0.3213
SMHB00193	鸡屎藤	Jishiteng	None	Paederiae Scandentis Herba	消食药	Digestants	4.53E-06	1.540e-04	0.400361	By_ingredient	0.8196
SMHB00194	鸡血藤	Jixueteng	Spatholobi Caulis	Suberect Spatholobus Stem	活血祛瘀药	Blood Activating Stasis Removing Drugs	8.83E-06	2.574e-04	0.780395	By_ingredient	0.9946
SMHB00195	积雪草	Jixuecao	Centellae Herba	Asiatic Pennywort Herb	清热解毒药	Antipyretic Detoxicate Drugs	2.51E-06	1.956e-04	0.631917	By_ingredient	0.9095
SMHB00196	急性子	Jixingzi	Impatientis Semen	seed of Garden Balsam	活血祛瘀药	Blood Activating Stasis Removing Drugs	0.00424304	0.0175455	1	By_ingredient	0.3412
SMHB00197	蒺藜	jili	Tribuli Fructus	Puncturevine Caltrop Fruit	平肝熄风药	Liver-Pacifying Wind-Extinguishing Medicinal	0.00128302	0.0181736	1	By_ingredient	0.5054
SMHB00199	降香	Jiangxiang	Dalbergiae Odoriferae Lignum	Odoriferous Rosewood	理气药	Qi Regulating Drugs	0.00188576	0.0228713	1	By_ingredient	0.6015
SMHB00200	绞股蓝	Jiaoguolan	None	Gynostemmae Pentaphylli Herba	补气药	Qi Reinforcing Drugs	0.311775	0.342116	1	By_ingredient	0.352
SMHB00202	金沸草	Jinfeicao	Inulae Herba	All - grass of Japanese Inula	化痰药	Phlegresolving Medicine	5.94E-05	0.00170372	1	By_ingredient	0.5772
SMHB00203	金果榄	Jinguolan	Tinosporae Radix	Tinosporae Radix	清热解毒药	Antipyretic Detoxicate Drugs	0.00454452	0.0182036	1	By_ingredient	0.302
SMHB00204	金莲花	Jinlianhua	Flos Trollii Chinensis	flower of Chinese Globeflower	清热解毒药	Antipyretic Detoxicate Drugs	6.79E-08	9.64E-06	0.0280942	By_ingredient	1.0101
SMHB00205	金钱草	Jinqiancao	Lysimachiae Herba	Christi Loosestrife	利水渗湿药	Diuretic Dampness Excreting Drugs	2.33E-05	5.198e-04	1	By_ingredient	0.7446
SMHB00206	金荞麦	Jinqiaomai	Fagopyri Dibotryis Rhizoma	Wild Buckwheat Rhizome	清热解毒药	Antipyretic Detoxicate Drugs	0.00582644	0.0239993	1	By_ingredient	0.4519
SMHB00207	金银花	Jinyinhua	Lonicerae Japonicae Flos	Honeysuckle Flower	清热解毒药	Antipyretic Detoxicate Drugs	1.07E-06	5.13E-05	0.0945666	By_ingredient	1.0146
SMHB00208	金樱子	Jinyingzi	Rosae Laevigatae Fructus	fruit of Cherokee Rose	收涩药	Astringent Medicinal	3.17E-09	4.84E-07	2.802e-04	By_ingredient	0.9758
SMHB00210	锦灯笼	Jindenglong	Physalis Calyx Seu Fructus	Calyx Seu Fructus Physalis	清热解毒药	Antipyretic Detoxicate Drugs	0.0012342	0.0176891	1	By_ingredient	0.4326
SMHB00212	荆芥	Jingjie	Schizonepetae Herba	all-grass of Fineleaf Schizonepeta	辛温解表药	Pungent-Warm Exterior-Releasing Medicinal	1.44E-06	6.44E-05	0.127267	By_ingredient	1.0932
SMHB00213	荆芥穗	Jingjiesui	Schizonepetae Spica	Schizonepetae Spica	辛温解表药	Pungent-Warm Exterior-Releasing Medicinal	1.95E-06	1.589e-04	0.597449	By_ingredient	0.7273
SMHB00214	景天三七	Jintiansanqi	None	Sedum Aizoon L.	化瘀止血药	Stasis-Resolving Hemostatic Medicinal	1.949e-04	0.00295341	1	By_ingredient	0.8889
SMHB00216	桔梗	Jiegeng	Platycodonis Radix	Platycodon Root	化痰药	Phlegresolving Medicine	0.0101859	0.0377156	1	By_ingredient	0.4851
SMHB00217	菊花	Juhua	Chrysanthemi Flos	Chrysanthemum Flower	辛凉解表药	Pungent Cool Diaphoretics	3.12E-06	1.163e-04	0.275746	By_ingredient	1.2132
SMHB00218	菊苣	Juju	Cichorii Herba Cichorii Radix	Chicory Herb	拔毒化腐生肌药	External Medicinal (Draw Out Toxin, Resolve Putridity)	3.38E-07	3.78E-05	0.139853	By_ingredient	1.1282
SMHB00220	橘红	Juhong	Citri Exocarpium Rubrum	Citri Exocarpium Rubrum	理气药	Qi Regulating Drugs	0.0299906	0.0718632	1	By_ingredient	0.2954
SMHB00221	橘络	Juluo	None	Citri Fructus Retinervus	理气药	Qi Regulating Drugs	0.0990858	0.124026	1	By_ingredient	0.1748
SMHB00222	橘叶	Juye	Folium Citri Tangerie	tangerine leaf	理气药	Qi Regulating Drugs	6.381e-04	0.0117804	1	By_ingredient	0.4697
SMHB00223	卷柏	Juanbai	Selaginellae Herba	aal-grass of Tamariskoid spikemoss	化瘀止血药	Stasis-Resolving Hemostatic Medicinal	3.92E-06	1.385e-04	0.34645	By_ingredient	0.6245
SMHB00224	决明子	Juemingzi	Cassiae Semen	Cassia Seed	清热泻火药	Fire Purging Drugs	0.00237155	0.0257383	1	By_ingredient	0.6791
SMHB00225	苦参	Kushen	Sophorae Flavescentis Radix	root of Lightyellow sophora	清热燥湿药	Heat-Clearing And Dampnessdrying Medicinal	1.48E-06	6.59E-05	0.130802	By_ingredient	1.1068
SMHB00227	苦丁茶	Kudingcha	None	Ilicis Cornutae Folium Immayuri	清热泻火药	Fire Purging Drugs	8.07E-06	2.401e-04	0.713227	By_ingredient	0.8002
SMHB00229	苦楝子	Kulianzi	None	Meliae Azedarach Fructus	驱虫药	Antiparasitic Drugs	0.0178995	0.0585784	1	By_ingredient	0.3119
SMHB00230	苦杏仁	Kuxingren	Armeniacae Semen Amarum	bitter Apricot seed	止咳平喘药	Antitussive Antiasthmetics	8.77E-06	5.220e-04	1	By_ingredient	0.8865
SMHB00231	款冬花	Kuandonghua	Farfarae Flos	flower bud of common coltsfoot	止咳平喘药	Antitussive Antiasthmetics	0.0313305	0.0522609	1	By_ingredient	0.3087
SMHB00232	昆布	Kunbu	Laminariae Thallus Eckloniae Thallus	Kelp or Tangle	化痰药	Phlegresolving Medicine	0.139	0.164917	1	By_ingredient	0.1482
SMHB00233	辣椒	Lajiao	Capsici Fructus	Hot pepper	拔毒化腐生肌药	External Medicinal (Draw Out Toxin, Resolve Putridity)	0.0383092	0.0798821	1	By_ingredient	0.5266
SMHB00234	莱菔子	Laifuzi	Raphani Semen	Radish Seed	消食药	Digestants	2.516e-04	0.00277379	1	By_ingredient	0.595
SMHB00235	老鹳草	Laoguancao	Erodii Herba Geranii Herba	All - grass of Common Heron's bill	祛风湿散寒药	Wind-Dampnessdispelling And Colddispersing Medicinal	8.82E-09	1.63E-06	0.0036492	By_ingredient	0.8713
SMHB00237	雷公藤	Leigongteng	Radix Tripterygii Wilfordii	root of common htreewingnut	祛风湿散寒药	Wind-Dampnessdispelling And Colddispersing Medicinal	0.00369906	0.0163544	1	By_ingredient	0.4874
SMHB00238	雷丸	Leiwan	Omphalia	Thunder Ball	驱虫药	Antiparasitic Drugs	8.548e-04	0.00650015	1	By_ingredient	0.4792
SMHB00239	荔枝核	Lizhihe	Litchi Semen	Litchi seed	理气药	Qi Regulating Drugs	0.0137563	0.0318317	1	By_ingredient	0.4236
SMHB00240	连钱草	Lianqiancao	Glechomae Herba	Glechomae Herba	利水渗湿药	Diuretic Dampness Excreting Drugs	2.39E-07	1.63E-05	0.0211228	By_ingredient	0.8997
SMHB00241	连翘	Lianqiao	Forsythiae Fructus	Weeping Forsythia Capsule	清热解毒药	Antipyretic Detoxicate Drugs	3.34E-08	3.38E-06	0.00295189	By_ingredient	1.2347
SMHB00242	莲房	Lianfang	Nelumbinis Receptaculum	Lotus Receptacle	收敛止血药	Astringent Hemostatic Medicinal	0.0172187	0.0360195	1	By_ingredient	0.1652
SMHB00243	莲须	Lianxu	Nelumbinis Stamen	Lotus stamen	收涩药	Astringent Medicinal	2.412e-04	0.00269347	1	By_ingredient	0.4396
SMHB00244	莲子心	Lianzixin	Nelumbinis Plumula	Lotus Plumule	收涩药	Astringent Medicinal	0.00276039	0.0141232	1	By_ingredient	0.3122
SMHB00245	两面针	Liangmianzhen	Zanthoxyli Radix	Shinyleaf Pricklyash Root	祛风湿散寒药	Wind-Dampnessdispelling And Colddispersing Medicinal	0.0360171	0.0774881	1	By_ingredient	0.3433
SMHB00247	蓼大青叶	Liaodaqingye	Polygoni Tinctorii Folium	Indigoplant Leaf	清热解毒药	Antipyretic Detoxicate Drugs	0.00133303	0.0186215	1	By_ingredient	0.5392
SMHB00248	灵芝	Lingzhi	Ganoderma	Lucid Ganoderma	补气药	Qi Reinforcing Drugs	0.371899	0.403259	1	By_ingredient	0.4851
SMHB00249	凌霄花	Lingxiaohua	Campsis Flos	flower of Common Trumpetcreeper	活血祛瘀药	Blood Activating Stasis Removing Drugs	3.290e-04	0.00334238	1	By_ingredient	0.7237
SMHB00250	零陵香	Linglingxiang	None	Lysimachiae Foenigraeci Herba	辛温解表药	Pungent-Warm Exterior-Releasing Medicinal	2.19E-05	4.959e-04	1	By_ingredient	0.8555
SMHB00251	龙胆	Longdan	Gentianae Radix Et Rhizoma	Gentiae Radix Et Rhozima	清热燥湿药	Heat-Clearing And Dampnessdrying Medicinal	9.75E-05	0.00246378	1	By_ingredient	0.8516
SMHB00252	龙葵	Longkui	None	Solanum Nigrum Linn.	清热解毒药	Antipyretic Detoxicate Drugs	2.02E-05	0.00101114	1	By_ingredient	0.8077
SMHB00254	漏芦	Loulu	Rhapontici Radix	Uniflower Swisscentaury Root	清热解毒药	Antipyretic Detoxicate Drugs	0.038548	0.08001	1	By_ingredient	0.2946
SMHB00255	芦根	Lugen	Phragmitis Rhizoma	Reed rhizome	清热泻火药	Fire Purging Drugs	9.312e-04	0.00689836	1	By_ingredient	0.4605
SMHB00256	芦荟	Luhui	Aloe	Aloe	攻下药	Offensive Purgative Medicinal	0.0100114	0.0474436	1	By_ingredient	0.4285
SMHB00257	鹿衔草	Luxiancao	Pyrolae Herba	all-grass Chinese pyrola	祛风湿散寒药	Wind-Dampnessdispelling And Colddispersing Medicinal	1.31E-09	2.22E-07	1.158e-04	By_ingredient	1.3531
SMHB00258	路路通	Lulutong	Liquidambaris Fructus	fruit of beautiful sweetgum	祛风湿散寒药	Wind-Dampnessdispelling And Colddispersing Medicinal	4.905e-04	0.00438814	1	By_ingredient	0.5186
SMHB00259	罗布麻叶	Luobumaye	Apocyni Veneti Folium	Apocyniveneti Folium	平肝熄风药	Liver-Pacifying Wind-Extinguishing Medicinal	8.46E-05	0.00290844	1	By_ingredient	0.561
SMHB00260	罗汉果	Luohanguo	Siraitiae Fructus	Grosvenor Momordica Fruit	化痰药	Phlegresolving Medicine	0.00250526	0.0264981	1	By_ingredient	0.6037
SMHB00261	络石藤	Luoshiteng	Trachelospermi Caulis Et Folium	stem of Chinese starjasmine	祛风湿清热药	Wind-Dampnessdispelling And Heatclearing Medicinal	0.00105071	0.016139	1	By_ingredient	0.4265
SMHB00262	绿豆	Lvdou	Semen Phaseoli Radiati	mung bean	清热解毒药	Antipyretic Detoxicate Drugs	0.00237723	0.0257383	1	By_ingredient	0.4804
SMHB00263	葎草	Lvcao	Herba Humuli Scandentis	all-grass of japanese hop	清热解毒药	Antipyretic Detoxicate Drugs	5.54E-06	3.670e-04	1	By_ingredient	1.0246
SMHB00264	麻黄	Mahuang	Ephedrae Herba	Ephedra	辛温解表药	Pungent-Warm Exterior-Releasing Medicinal	1.15E-08	1.42E-06	0.00101637	By_ingredient	1.5406
SMHB00265	麻黄根	Mahuanggen	Ephedrae Radix Et Rhizoma	Ephedra Root	收涩药	Astringent Medicinal	1.29E-07	1.01E-05	0.011401	By_ingredient	0.8135
SMHB00266	马鞭草	Mabiancao	Verbenae Herba	European Verbe Herb	活血祛瘀药	Blood Activating Stasis Removing Drugs	4.27E-08	4.16E-06	0.00377383	By_ingredient	0.9838
SMHB00268	马齿苋	Machixian	Portulacae Herba	all-grass of Purslane	清热解毒药	Antipyretic Detoxicate Drugs	3.72E-09	5.53E-07	3.288e-04	By_ingredient	0.8746
SMHB00270	马钱子	Maqianzi	Strychni Semen	Nux Vomica	祛风湿散寒药	Wind-Dampnessdispelling And Colddispersing Medicinal	0.116187	0.141685	1	By_ingredient	0.1275
SMHB00271	马尾莲	Maweilian	Radix Et Rhizoma Thalictri	root and rhizome of Manyleaf Meadowrue	清热燥湿药	Heat-Clearing And Dampnessdrying Medicinal	0.0316906	0.0526158	1	By_ingredient	0.3013
SMHB00272	麦芽	Maiya	Hordei Fructus Germinatus	Germited Barley	消食药	Digestants	2.68E-05	0.00124997	1	By_ingredient	0.7208
SMHB00273	满山红	Manshanhong	Rhododendri Daurici Folium	Dahurian Rhododendron Leaf	止咳平喘药	Antitussive Antiasthmetics	2.466e-04	0.00273814	1	By_ingredient	0.9877
SMHB00274	蔓荆子	Manjingzi	Viticis Fructus	Shrub Chastetree Fruit	辛凉解表药	Pungent Cool Diaphoretics	5.35E-06	2.297e-04	0.671688	By_ingredient	1.1505
SMHB00275	猫眼草	Maoyancao	None	Lunulatae Herba	化痰药	Phlegresolving Medicine	1.765e-04	0.00216907	1	By_ingredient	0.4442
SMHB00277	没药	Moyao	Myrrha	Myrrh	活血祛瘀药	Blood Activating Stasis Removing Drugs	0.00970195	0.0470129	1	By_ingredient	1.027
SMHB00278	玫瑰花	Meiguihua	Rosae Rugosae Flos	Rose Flower	理气药	Qi Regulating Drugs	0.00309207	0.0299052	1	By_ingredient	0.5931
SMHB00279	梅花	Meihua	Mume Flos	Plum Flower	理气药	Qi Regulating Drugs	0.00216866	0.0123174	1	By_ingredient	0.7024
SMHB00280	猕猴桃根	Mihoutaogen	None	Actinidia Chinensis Planch	清热解毒药	Antipyretic Detoxicate Drugs	1.17E-06	1.043e-04	0.26514	By_ingredient	0.9321
SMHB00281	密蒙花	Mimenghua	Buddlejae Flos	flower of Pale Butterflybush	清热泻火药	Fire Purging Drugs	1.330e-04	0.00303048	1	By_ingredient	0.6147
SMHB00283	墨旱莲	Mohanlian	Ecliptae Herba	all-grass of Yerbadetajo	补阴药	Yin-Tonifying Medicinal	3.00E-06	1.128e-04	0.26514	By_ingredient	0.8916

Herb id	Chinese name	Pinyin name	Latin name	English name	Class in Chinese	Class in English	P_value	FDR(BH)	FDR(Bonferroni)	Relationship	Inferred evidence score
SMHB00284	牡丹皮	Mudanpi	Moutan Cortex	Tree Peony Bark	清热凉血药	Heat Clearing Blood Cooling Drugs	5.75E-06	3.781e-04	1	By_ingredient	1.2121
SMHB00285	牡荆叶	Mujingye	Viticis Negundo Foliun	Hempleaf Negundo Chastetree Leaf	辛凉解表药	Pungent Cool Diaphoretics	0.0288388	0.0708579	1	By_ingredient	0.2909
SMHB00286	牡荆子	Mujingzi	None	Viticis Canbifoliae Fructus	止咳平喘药	Antitussive Antiasthmetics	0.282176	0.311832	1	By_ingredient	0.273
SMHB00287	木鳖子	Mubiezi	Momordicae Semen	Momordicae Semen	拔毒化腐生肌药	External Medicinal (Draw Out Toxin, Resolve Putridity)	0.00229633	0.0127743	1	By_ingredient	0.3169
SMHB00288	木瓜	Mugua	Chaenomelis Fructus	fruit of Common Floweringquince	祛风湿散寒药	Wind-Dampnessdispelling And Colddispersing Medicinal	4.893e-04	0.00437909	1	By_ingredient	0.6303
SMHB00289	木蝴蝶	Muhudie	Oroxyli Semen	Indian Trumpetflower Seed	清热解毒药	Antipyretic Detoxicate Drugs	2.79E-05	7.674e-04	1	By_ingredient	0.663
SMHB00290	木棉花	Mumianhua	Gossampini Flos	Flower of Common Bombax	拔毒化腐生肌药	External Medicinal (Draw Out Toxin, Resolve Putridity)	2.14E-05	4.875e-04	1	By_ingredient	1.0161
SMHB00292	木香	Muxiang	Aucklandiae Radix	Common Aucklandia Root	理气药	Qi Regulating Drugs	0.0222568	0.0419611	1	By_ingredient	0.4694
SMHB00293	木贼	Muzei	Equiseti Hiemalis Herba	Common Scouring Rush Herb	辛凉解表药	Pungent Cool Diaphoretics	2.82E-10	5.86E-08	2.49E-05	By_ingredient	1.2197
SMHB00294	墓头回	Mutouhui	None	Et Radix Patriniae Rhizoma	清热解毒药	Antipyretic Detoxicate Drugs	0.0108635	0.0277956	1	By_ingredient	0.2981
SMHB00296	南鹤虱	Nanheshi	Carotae Fructus	Fructus Carotae	驱虫药	Antiparasitic Drugs	1.74E-05	4.201e-04	1	By_ingredient	0.6958
SMHB00297	南沙参	Nanshashen	Adenophorae Radix	Fourleaf Ladybell Root	补阴药	Yin-Tonifying Medicinal	0.171429	0.198212	1	By_ingredient	0.1741
SMHB00298	南蛇藤果	nshetengguo	None	Celastri Orbiculati Fructus	安神药	Tranguilizing Medicinal	2.758e-04	0.00666189	1	By_ingredient	0.5838
SMHB00299	南五味子	Nanwuweizi	Schisandrae Sphenantherae Fructus	Schisandrae Sphentherae Fructus	收涩药	Astringent Medicinal	0.0107264	0.048976	1	By_ingredient	0.4389
SMHB00300	闹羊花	Naoyanghua	Rhododendri Mollis Flos	Yellow Azalea Flower	祛风湿散寒药	Wind-Dampnessdispelling And Colddispersing Medicinal	0.00362813	0.026726	1	By_ingredient	0.6343
SMHB00301	牛蒡子	Niubangzi	Arctil Fructus	achene of Great Burdock	辛凉解表药	Pungent Cool Diaphoretics	0.0380604	0.0598164	1	By_ingredient	0.2908
SMHB00303	牛黄	Niuhuang	Bovis Calculus	Cow-bezoar	开窍药	Resuscitative Stimulant; Resuscitative Medicinal	1.39E-06	6.27E-05	0.122848	By_ingredient	0.6784
SMHB00304	牛膝	Niuxi	Achyranthis Bidentatae Radix	root of Twotooth Achyranthes	活血祛瘀药	Blood Activating Stasis Removing Drugs	0.00553508	0.019827	1	By_ingredient	0.4771
SMHB00305	糯稻根	Nuodaogen	Radix Oryzae Glutinosae	Glutinous Rice root	收涩药	Astringent Medicinal	0.167078	0.19368	1	By_ingredient	0.1529
SMHB00309	佩兰	Peilan	Eupatorii Herba	Fortune Eupatorium Herb	化湿药	Dampness Removing Drugs	0.128426	0.154193	1	By_ingredient	0.3199
SMHB00310	枇杷叶	Pipaye	Eriobotryae Folium	Loquat Leaf	止咳平喘药	Antitussive Antiasthmetics	0.00191347	0.0113437	1	By_ingredient	0.9157
SMHB00312	蒲黄	Puhuang	Typhae Pollen	pollen of longbract cattail	化瘀止血药	Stasis-Resolving Hemostatic Medicinal	3.44E-06	1.255e-04	0.304027	By_ingredient	0.5904
SMHB00313	荠菜	Jicai	Herba Capsellae	all-grass of Shepherdspurse	利水渗湿药	Diuretic Dampness Excreting Drugs	9.110e-04	0.0147947	1	By_ingredient	0.7274
SMHB00314	千斤拔	Qianjinba	Radix Moghaniae Philippinensis	root of Philippine Flemingia	祛风湿筋骨药	Wind-Damp-Dispelling And Sinew-Bone-Strengthening Medicinal	0.00308862	0.0298942	1	By_ingredient	0.4649
SMHB00318	牵牛子	Qianniuzi	Pharbitidis Semen	Pharbitis seed	通水药	Drastic Purgatives	0.0360171	0.0774881	1	By_ingredient	0.3384
SMHB00319	前胡	Qianhu	Peucedani Radix	root of Grand Hogfennel	化痰药	Phlegresolving Medicine	1.30E-05	7.121e-04	1	By_ingredient	1.0661
SMHB00320	芡实	Qianshi	Euryales Semen	seed of Gordon Euryale	收涩药	Astringent Medicinal	0.0111034	0.0497217	1	By_ingredient	0.2781
SMHB00321	茜草	Qiancao	Rubiae Radix Et Rhizoma	root of Tali Madder	化瘀止血药	Stasis-Resolving Hemostatic Medicinal	0.123856	0.149472	1	By_ingredient	0.3698
SMHB00322	羌活	Qianghuo	Notopterygii Rhizoma Et Radix	Root and rhizome of Incised Notoptergium	辛温解表药	Pungent-Warm Exterior-Releasing Medicinal	0.0946526	0.119357	1	By_ingredient	0.4878
SMHB00324	秦艽	Qinjiao	Gentianae Macrophyllae Radix	root of Largeleaf Gentian	祛风湿清热药	Wind-Dampnessdispelling And Heatclearing Medicinal	0.00501482	0.019008	1	By_ingredient	0.5414
SMHB00325	秦皮	Qinpi	Fraxini Cortex	bark of Largeleaf Chinese Ash	清热燥湿药	Heat-Clearing And Dampnessdrying Medicinal	0.159776	0.18622	1	By_ingredient	0.131
SMHB00327	青风藤	Qingfengteng	Sinomenii Caulis	Caulis Sinomenii	祛风湿散寒药	Wind-Dampnessdispelling And Colddispersing Medicinal	0.00424304	0.0175455	1	By_ingredient	0.3563
SMHB00328	青果	Qingguo	Canarii Fructus	Carii Fructus	清热解毒药	Antipyretic Detoxicate Drugs	0.00673643	0.0219822	1	By_ingredient	0.4931
SMHB00329	青蒿	Qinghao	Artemisiae Annuae Herba	Sweet Wormwood Herb	清虚热药	Asthenic Heat Dispelling Drugs	1.346e-04	0.001797	1	By_ingredient	0.763
SMHB00330	青皮	Qingpi	Citri Reticulatae Pericarpium Viride	Green Tangerine Peel	理气药	Qi Regulating Drugs	0.0752473	0.0992624	1	By_ingredient	0.1763
SMHB00331	青葙子	Qingxiangzi	Celosiae Semen	Celosiae Semen	清热泻火药	Fire Purging Drugs	0.0491449	0.0716039	1	By_ingredient	0.1766
SMHB00332	青叶胆	Qingyedan	Swertiae Mileensis Herba	All - grass of Mile Swertia	利水渗湿药	Diuretic Dampness Excreting Drugs	0.0687876	0.0924027	1	By_ingredient	0.366
SMHB00334	瞿麦	Qumai	Dianthi Herba	all-grass of Lilac Pink	利水渗湿药	Diuretic Dampness Excreting Drugs	0.120796	0.146409	1	By_ingredient	0.139
SMHB00335	拳参	Quanshen	Bistortae Rhizoma	rhizome of Bistort	清热解毒药	Antipyretic Detoxicate Drugs	0.0118747	0.0501663	1	By_ingredient	0.2895
SMHB00336	人参	Renshen	Ginseng Radix Et Rhizoma	Ginseng	补气药	Qi Reinforcing Drugs	0.00197454	0.0225739	1	By_ingredient	1.0038
SMHB00337	人参叶	Renshenye	Ginseng Folium	Ginseng Leaf	补气药	Qi Reinforcing Drugs	0.0130175	0.0434475	1	By_ingredient	0.4657
SMHB00338	肉苁蓉	Roucongrong	Cistanches Herba	Desertliving Cistanche	补阳药	Yang Reinforcing Drugs	3.197e-04	0.00327716	1	By_ingredient	0.511
SMHB00341	乳香	Ruxiang	Olibanum	Frankincense	活血祛瘀药	Blood Activating Stasis Removing Drugs	0.00150542	0.0107117	1	By_ingredient	0.5716
SMHB00342	蕤仁	Ruiren	Prinsepiae Nux	Hedge Prinsepia Nut	辛凉解表药	Pungent Cool Diaphoretics	7.84E-09	1.47E-06	0.00324334	By_ingredient	1.1061
SMHB00343	三白草	Sanbaicao	Saururi Herba	Chinese Lizardtail Rhizome or Herb	利水渗湿药	Diuretic Dampness Excreting Drugs	0.116187	0.141685	1	By_ingredient	0.1546
SMHB00344	三分三	Sanfensan	None	Anisodus Acutangulus C. Y. Wu Et C .Chen Ex C. Che	祛风湿散寒药	Wind-Dampnessdispelling And Colddispersing Medicinal	0.0816634	0.105919	1	By_ingredient	0.1458
SMHB00345	三棱	Sanleng	Sparganii Rhizoma	Common Burreed Tuber	活血祛瘀药	Blood Activating Stasis Removing Drugs	0.023886	0.065638	1	By_ingredient	0.2906
SMHB00346	三七	Sanqi	Notoginseng Radix Et Rhizoma	Sanchi	化瘀止血药	Stasis-Resolving Hemostatic Medicinal	0.00646269	0.0214953	1	By_ingredient	0.4731
SMHB00348	桑白皮	Sangbaipi	Mori Cortex	Cortex Mori	止咳平喘药	Antitussive Antiasthmetics	3.162e-04	0.00325066	1	By_ingredient	0.9107
SMHB00349	桑寄生	Sangjisheng	Taxilli Herba	Chinese Taxillus Herb	祛风湿筋骨药	Wind-Damp-Dispelling And Sinew-Bone-Strengthening Medicinal	3.35E-05	0.00147501	1	By_ingredient	0.813
SMHB00350	桑椹	Sangshen	Mori Fructus	Mulberry Fruit	补阴药	Yin-Tonifying Medicinal	1.854e-04	0.00509277	1	By_ingredient	0.7315
SMHB00351	桑叶	Sangye	Mori Folium	Mulberry Leaf	辛凉解表药	Pungent Cool Diaphoretics	5.771e-04	0.00489427	1	By_ingredient	0.8666
SMHB00352	桑枝	Sangzhi	Mori Ramulus	Mulberry Twig	祛风湿清热药	Wind-Dampnessdispelling And Heatclearing Medicinal	0.0162561	0.0377156	1	By_ingredient	0.5248
SMHB00353	沙棘	Shaji	Hippophae Fructus	fruit of seabuckthorn	化痰药	Phlegresolving Medicine	8.45E-07	4.31E-05	0.0746811	By_ingredient	1.3001
SMHB00354	沙苑子	Shayuanzi	Astragali Complanati Semen	Flatstem Milkvetch Seed	补阳药	Yang Reinforcing Drugs	3.076e-04	0.00319396	1	By_ingredient	0.6209
SMHB00355	砂仁	Sharen	Amomi Fructus	Villous Amomum Fruit	化湿药	Dampness Removing Drugs	2.209e-04	0.00573417	1	By_ingredient	0.915
SMHB00357	山豆根	Shandougen	Sophorae Tonkinensis Radix Et Rhizoma	Vietmese Sophora Root	清热解毒药	Antipyretic Detoxicate Drugs	6.05E-09	8.37E-07	5.347e-04	By_ingredient	1.1352
SMHB00358	山柰	Shannai	Kaempferiae Rhizoma	Galanga Resurrectionlily Rhizome	温里药	Warming Interior Drugs	0.100654	0.125611	1	By_ingredient	0.1763
SMHB00360	山楂叶	Shanzha	Crataegi Folium	Crataegi Folium	活血祛瘀药	Blood Activating Stasis Removing Drugs	7.94E-09	1.33E-06	9.368e-04	By_ingredient	1.3647
SMHB00361	山茱萸	Shanzhuyu	Corni Fructus	Asiatic Cornelian Cherry Fruit	收涩药	Astringent Medicinal	5.864e-04	0.0105079	1	By_ingredient	1.0294
SMHB00362	商陆	Shanglu	Phytolaccae Radix	Pokeberry Root	通水药	Drastic Purgatives	0.100654	0.125611	1	By_ingredient	0.1762
SMHB00364	射干	Shegan	Belamcandae Rhizoma	Blackberrylily Rhizome	清热解毒药	Antipyretic Detoxicate Drugs	3.63E-05	0.00157243	1	By_ingredient	0.8359
SMHB00366	升麻	Shengma	Cimicifugae Rhizoma	Largetrifoliolious Bugbane Rhizome	辛凉解表药	Pungent Cool Diaphoretics	0.00446693	0.0345697	1	By_ingredient	0.6806
SMHB00368	十大功劳根	Shidagonglaogen	None	Mahoniae Eadix	清热解毒药	Antipyretic Detoxicate Drugs	0.00523813	0.0369661	1	By_ingredient	0.2973
SMHB00370	石菖蒲	Shichangpu	Acori Tatarinowii Rhizoma	Grassleaf Sweetflag Rhizome	开窍药	Resuscitative Stimulant; Resuscitative Medicinal	0.0153146	0.0337508	1	By_ingredient	0.5162
SMHB00371	石见穿	Shijianchuan	None	Salviae Chinensis Herba	活血祛瘀药	Blood Activating Stasis Removing Drugs	6.44E-05	0.00108166	1	By_ingredient	0.4809
SMHB00372	石榴皮	Shiliupi	Granati Pericarpium	Pomegrate Rind	收涩药	Astringent Medicinal	3.64E-07	2.23E-05	0.0321703	By_ingredient	0.8881
SMHB00373	石上柏	Shishangbai	None	Selaginella Doederleinii Hieron	清热解毒药	Antipyretic Detoxicate Drugs	0.00143671	0.0151362	1	By_ingredient	0.5028
SMHB00374	石韦	Shiwei	Pyrrosiae Folium	Pyrrosiae Folium	利水渗湿药	Diuretic Dampness Excreting Drugs	1.95E-06	1.385e-04	0.34645	By_ingredient	0.8009
SMHB00376	柿蒂	Shidi	Kaki Calyx	calyx and receptacle of a Persimmon	理气药	Qi Regulating Drugs	3.67E-06	1.316e-04	0.324355	By_ingredient	0.6258
SMHB00377	熟地黄	Shudihuang	Rehmanniae Radix Praeparata	Prepared Rehmannia Root	补血药	Blood-Tonifying Medicinal	0.00275822	0.0280192	1	By_ingredient	0.5359
SMHB00378	鼠曲草	Shuqucao	None	Gphalii Affinis Herba	化痰药	Phlegresolving Medicine	1.04E-06	6.83E-05	0.136989	By_ingredient	0.8638
SMHB00379	水飞蓟	Shuifeiji	Silybi Fructus	Silybum Marianum	清热解毒药	Antipyretic Detoxicate Drugs	3.04E-07	3.45E-05	0.125753	By_ingredient	0.9303
SMHB00380	水红花子	Shuihonghuazi	Polygoni Orientalis Fructus	Prince's-feather Fruit	活血祛瘀药	Blood Activating Stasis Removing Drugs	2.412e-04	0.00269347	1	By_ingredient	0.444
SMHB00381	四季青	Sijiqing	Ilicis Chinensis Folium	Purpleflower Holly Leaf	清热解毒药	Antipyretic Detoxicate Drugs	6.76E-06	2.104e-04	0.597449	By_ingredient	0.5351
SMHB00382	松花粉	Songhuafen	Pini Pollen	Pine Pollen	收敛止血药	Astringent Hemostatic Medicinal	2.82E-05	0.0012971	1	By_ingredient	0.5091
SMHB00383	油松节	Songjie	Pini Lignum Nodi	Knotty pine wood, Pine nodular branch	祛风湿散寒药	Wind-Dampnessdispelling And Colddispersing Medicinal	0.012032	0.0293794	1	By_ingredient	0.3024
SMHB00384	松子仁	Songziren	None	Semen Pini Koraiensis	润下药	Laxatives	0.010366	0.0272241	1	By_ingredient	0.1557

Herb id	Chinese name	Pinyin name	Latin name	English name	Class in Chinese	Class in English	P_value	FDR(BH)	FDR(Bonferroni)	Relationship	Inferred evidence score
SMHB00385	苏合香	Suhexiang	Styrax	Storax	开窍药	Resuscitative Stimulant; Resuscitative Medicinal	0.0959421	0.120753	1	By_ingredient	0.334
SMHB00387	娑罗子	Suoluozi	Aesculi Semen	Buckeye Seed	理气药	Qi Regulating Drugs	8.47E-07	7.96E-05	0.350441	By_ingredient	0.7434
SMHB00388	太子参	Taizishen	Pseudostellariae Radix	Heterophylly Falsestarwort Root	补气药	Qi Reinforcing Drugs	3.01E-05	6.277e-04	1	By_ingredient	0.5837
SMHB00389	檀香	Tanxiang	Santali Albi Lignum	Sandalwood	理气药	Qi Regulating Drugs	0.00953232	0.0261822	1	By_ingredient	0.4621
SMHB00390	桃仁	Taoren	Persicae Semen	Peach Seed	活血祛瘀药	Blood Activating Stasis Removing Drugs	0.00661139	0.0287235	1	By_ingredient	0.4866
SMHB00391	天冬	Tiandong	Asparagi Radix	Cochinchinese Asparagus Root	补阴药	Yin-Tonifying Medicinal	0.0103552	0.0480349	1	By_ingredient	0.3005
SMHB00392	天花粉	Tianhuafen	Trichosanthis Radix	Trichosanthes root, Skegourd root	清热泻火药	Fire Purging Drugs	0.0341495	0.0554487	1	By_ingredient	0.145
SMHB00393	天葵子	Tiankuizi	Semiaquilegiae Radix	Muskroot-like Semiaquilegia Root	清热解毒药	Antipyretic Detoxicate Drugs	0.139	0.164917	1	By_ingredient	0.1615
SMHB00394	天南星	Tiannanxing	Arisaematis Rhizoma	Jackinthepulpit Tuber	化痰药	Phlegresolving Medicine	6.688e-04	0.0122155	1	By_ingredient	0.6124
SMHB00395	天山雪莲	Tianshanxuelian	Saussureae Involucratae Herba	Saussureae Involucratae Herba	补阳药	Yang Reinforcing Drugs	1.45E-05	3.680e-04	1	By_ingredient	0.5512
SMHB00397	天仙子	Tianxianzi	Hyoscyami Semen	Henbane Seed	止咳平喘药	Antitussive Antiasthmetics	0.114646	0.140111	1	By_ingredient	0.1758
SMHB00398	天竺黄	Tianzhuhuang	Bambusae Concretio Silicea	Chinese schinzostachyum Tabasheer	化痰药	Phlegresolving Medicine	0.0366435	0.0782226	1	By_ingredient	0.3091
SMHB00399	甜瓜子	Tianguazi	Melo Semen	muskmelon seed	化痰药	Phlegresolving Medicine	0.0425084	0.0644694	1	By_ingredient	0.1699
SMHB00401	铁线透骨草	Tiexiantougucao	None	Herba Clematidis Aethusaefoliae	祛风湿散寒药	Wind-Dampnessdispelling And Colddispersing Medicinal	1.00E-06	9.18E-05	0.414287	By_ingredient	0.5741
SMHB00402	葶苈子	Tinglizi	Descurainiae Semen Lepidii Semen	Lepidium seed, Descurainia seed	止咳平喘药	Antitussive Antiasthmetics	0.00828014	0.0242077	1	By_ingredient	0.3008
SMHB00405	土茯苓	Tufuling	Smilacis Glabrae Rhizoma	Glabrous Greenbrier Rhizome	清热解毒药	Antipyretic Detoxicate Drugs	5.45E-08	7.99E-06	0.0225185	By_ingredient	1.1874
SMHB00407	菟丝子	Tusizi	Cuscutae Semen	Dodder Seed	补阳药	Yang Reinforcing Drugs	3.28E-05	6.637e-04	1	By_ingredient	0.4555
SMHB00409	瓦松	Wasong	Orostachyis Fimbriatae Herba	Cotyledon Fimbriata Turcz.	清热凉血药	Heat Clearing Blood Cooling Drugs	1.69E-05	4.111e-04	1	By_ingredient	0.6194
SMHB00410	王不留行	Wangbuliuxing	Vaccariae Semen	Vacarria seed, Cow soapwort seed	活血祛瘀药	Blood Activating Stasis Removing Drugs	0.00963048	0.0469112	1	By_ingredient	0.2673
SMHB00411	威灵仙	Weilingxian	Clematidis Radix Et Rhizoma	Chinese Clematis Root	祛风湿散寒药	Wind-Dampnessdispelling And Colddispersing Medicinal	0.00848328	0.0246321	1	By_ingredient	0.4728
SMHB00412	委陵菜	Weilingcai	Potentiliae Chinensis Herba	Chinese Cinquefoil	清热解毒药	Antipyretic Detoxicate Drugs	4.92E-11	1.25E-08	4.35E-06	By_ingredient	1.1407
SMHB00413	乌敛莓	Wulianmei	None	Cayratiae Japonicae Herba	清热解毒药	Antipyretic Detoxicate Drugs	8.548e-04	0.00650015	1	By_ingredient	0.2717
SMHB00414	乌梅	Wumei	Mume Fructus	Smoked Plum	收涩药	Astringent Medicinal	6.60E-07	3.55E-05	0.0583308	By_ingredient	0.8318
SMHB00415	乌药	Wuyao	Linderae Radix	Combined Spicebush Root	理气药	Qi Regulating Drugs	0.188614	0.215946	1	By_ingredient	0.3406
SMHB00417	无患子皮	Wuhuanzipi	None	Sapindi Mukorossiperic Arpium	化痰药	Phlegresolving Medicine	0.00792313	0.0435365	1	By_ingredient	0.3008
SMHB00418	吴茱萸	Wuzhuyu	Euodiae Fructus	Medicil Evodia Fruit	温里药	Warming Interior Drugs	0.0369029	0.05851	1	By_ingredient	0.6237
SMHB00419	梧桐子	Wutongzi	None	Semen Firmiae	消食药	Digestants	0.044508	0.0860905	1	By_ingredient	0.1752
SMHB00421	西河柳	Xiheliu	Tamaricis Cacumen	Chinese Tamarisk Twig	辛温解表药	Pungent-Warm Exterior-Releasing Medicinal	7.15E-05	0.0025666	1	By_ingredient	0.7528
SMHB00422	西红花	Xihonghua	Croci Stigma	Saffron	活血祛瘀药	Blood Activating Stasis Removing Drugs	0.0063734	0.0213584	1	By_ingredient	0.559
SMHB00423	西洋参	Xiyangshen	Panacis Quinquefolii Radix	American Ginseng	补气药	Qi Reinforcing Drugs	0.226026	0.254331	1	By_ingredient	0.1869
SMHB00425	细辛	Xixin	Asari Radix Et Rhizoma	all-grass Manchurian wildginger	辛温解表药	Pungent-Warm Exterior-Releasing Medicinal	0.283434	0.313124	1	By_ingredient	0.3682
SMHB00426	夏枯草	Xiakucao	Prunellae Spica	fruit -spike of Common Selfheal	清热泻火药	Fire Purging Drugs	5.90E-08	5.40E-06	0.00521442	By_ingredient	0.7537
SMHB00428	仙鹤草	Xianhecao	Agrimoniae Herba	all - grss of Hairyvein Agrimonia	收敛止血药	Astringent Hemostatic Medicinal	8.29E-09	1.09E-06	7.327e-04	By_ingredient	0.8267
SMHB00430	仙桃草	Xiantaocao	None	Veronicae Peregrie Herba	活血祛瘀药	Blood Activating Stasis Removing Drugs	1.75E-05	4.215e-04	1	By_ingredient	0.363
SMHB00431	相思子	Xiangsizi	None	Abrus Precatorius L.	清热解毒药	Antipyretic Detoxicate Drugs	0.00165895	0.0214063	1	By_ingredient	0.4641
SMHB00432	香附	Xiangfu	Cyperi Rhizoma	rhizome of Nutgrass Galingale	理气药	Qi Regulating Drugs	3.07E-05	6.368e-04	1	By_ingredient	0.9647
SMHB00433	香加皮	Xiangjiapi	Periplocae Cortex	Cortex Periplocae Radicis	祛风湿散寒药	Wind-Dampnessdispelling And Colddispersing Medicinal	0.00807925	0.0239993	1	By_ingredient	0.4703
SMHB00434	香茅	Xiangmao	None	Cymbopon Citratus(D C.) Stapf	祛风湿散寒药	Wind-Dampnessdispelling And Colddispersing Medicinal	0.0441717	0.0663769	1	By_ingredient	0.1463
SMHB00435	香薷	Xiangru	Moslae Herba	all-grass of Haichow Elsholtzia	辛温解表药	Pungent-Warm Exterior-Releasing Medicinal	7.60E-06	2.297e-04	0.671688	By_ingredient	0.8672
SMHB00436	香橼	Xiangyuan	Citri Fructus	Citri Fructus	理气药	Qi Regulating Drugs	0.00245063	0.026213	1	By_ingredient	0.4694
SMHB00437	小茴香	Xiaohuixiang	Foeniculi Fructus	fennel fruit	温里药	Warming Interior Drugs	0.0752473	0.0992624	1	By_ingredient	0.1718
SMHB00438	小蓟	Xiaoji	Cirsii Herba	Field Thistle Herb	凉血止血药	Blood-Cooling Hemostatic Medicinal	1.58E-05	8.354e-04	1	By_ingredient	0.5679
SMHB00439	小通草	Xiaotongcao	Stachyuri Medulla Helwingiae Medulla	Stachyurus or Japanese Helwingia Pith	利水渗湿药	Diuretic Dampness Excreting Drugs	0.0391733	0.0609155	1	By_ingredient	0.1764
SMHB00440	薤白	Xiebai	Allii Macrostemonis Bulbus	bulb of longstamen onion	理气药	Qi Regulating Drugs	0.140501	0.166456	1	By_ingredient	0.1313
SMHB00442	徐长卿	Xuchangqing	Cynanchi Paniculati Radix Et Rhizoma	Paniculate Swallowwort Root	祛风湿散寒药	Wind-Dampnessdispelling And Colddispersing Medicinal	0.0254943	0.0672724	1	By_ingredient	0.3393
SMHB00443	续断	Xuduan	Dipsaci Radix	Himalayan Teasel Root	补阳药	Yang Reinforcing Drugs	0.00636861	0.0398514	1	By_ingredient	0.3039
SMHB00444	萱草根	Xuancaogen	None	Hemerocallis Radix	利水渗湿药	Diuretic Dampness Excreting Drugs	0.0441717	0.0663769	1	By_ingredient	0.1772
SMHB00445	玄参	Xuanshen	Scrophulariae Radix	Figwort Root	清热凉血药	Heat Clearing Blood Cooling Drugs	3.13E-05	0.00139923	1	By_ingredient	0.9119
SMHB00446	旋覆花	Xuanfuhua	Inulae Flos	Inula Flower	化痰药	Phlegresolving Medicine	9.95E-05	0.0014516	1	By_ingredient	0.6839
SMHB00448	鸦胆子	Yadanzi	Bruceae Fructus	Java Brucea Fruit	清热解毒药	Antipyretic Detoxicate Drugs	0.108453	0.133728	1	By_ingredient	0.3088
SMHB00449	鸭跖草	Yazhicao	Commelinae Herba	all-grass of Common Dayflower	清热泻火药	Fire Purging Drugs	5.63E-05	0.00216219	1	By_ingredient	0.6025
SMHB00451	延胡索	Yanhusuo	Rhizoma Corydalis,Corydalis Rhizoma	Corydalis tuber	理气药	Qi Regulating Drugs	0.0493143	0.0902498	1	By_ingredient	0.3209
SMHB00452	洋金花	Yangjinhua	Daturae Flos	Datura Flower	止咳平喘药	Antitussive Antiasthmetics	7.495e-04	0.00590754	1	By_ingredient	0.5186
SMHB00453	野菊花	Yejuhua	Chrysanthemi Indici Flos	Wild Chrysanthemum Flower	清热解毒药	Antipyretic Detoxicate Drugs	3.90E-09	7.97E-07	0.00161311	By_ingredient	1.1121
SMHB00454	一枝黄花	Yizhihuanghua	Solidaginis Herba	Solidaginis Herba	辛凉解表药	Pungent Cool Diaphoretics	0.00267921	0.0264223	1	By_ingredient	0.4774
SMHB00456	益母草	Yimucao	Leonuri Herba	motherwort	活血祛瘀药	Blood Activating Stasis Removing Drugs	9.39E-05	0.00139641	1	By_ingredient	0.4394
SMHB00457	益智	Yizhi	Alpiniae Oxyphyllae Fructus	Alpiniae Oxyphyliae Fructus	补阳药	Yang Reinforcing Drugs	0.00607645	0.0390382	1	By_ingredient	0.3195
SMHB00459	茵陈	Yinchen	Artemisiae Scopariae Herba	Virgate Wormwood Herb	利水渗湿药	Diuretic Dampness Excreting Drugs	0.0880363	0.112491	1	By_ingredient	0.1547
SMHB00460	银柴胡	Yincha	Stellariae Radix	Starwort Root	清虚热药	Asthenic Heat Dispelling Drugs	0.0213034	0.0631816	1	By_ingredient	0.3508
SMHB00461	银杏叶	Yinxingye	Ginkgo Folium	Ginkgo Folium	止咳平喘药	Antitussive Antiasthmetics	3.45E-05	0.0015067	1	By_ingredient	1.0396
SMHB00462	淫羊藿	Yinyanghuo	Epimedii Folium	Epimrdii Herba	补阳药	Yang Reinforcing Drugs	2.63E-06	1.020e-04	0.232439	By_ingredient	0.9576
SMHB00464	余甘子	Yuganzi	Phyllanthi Fructus	Emblic Leafflower Fruit	清热解毒药	Antipyretic Detoxicate Drugs	9.84E-06	5.723e-04	1	By_ingredient	0.7841
SMHB00465	鱼腥草	Yuxingcao	Houttuyniae Herba	all-grass of Heartleaf Houttuynia	清热解毒药	Antipyretic Detoxicate Drugs	3.965e-04	0.0085604	1	By_ingredient	0.6402
SMHB00466	禹州漏芦	Yuzhouloulu	Echinopsis Radix	Globethistle Root	拔毒化腐生肌药	External Medicinal (Draw Out Toxin, Resolve Putridity)	9.39E-05	0.00139641	1	By_ingredient	0.4959
SMHB00467	玉米须	Yumixu	Stigma Maydis	Corn stigma	利水渗湿药	Diuretic Dampness Excreting Drugs	0.00326253	0.0296664	1	By_ingredient	0.3964
SMHB00469	郁金	Yujin	Curcumae Radix	Tuber - root of Common Turmeric	活血祛瘀药	Blood Activating Stasis Removing Drugs	0.00187747	0.0228145	1	By_ingredient	0.8072
SMHB00470	郁李仁	Yuliren	Pruni Semen	Chinese Dwarf Cherry Seed	润下药	Laxatives	1.04E-06	5.02E-05	0.0919152	By_ingredient	0.4621
SMHB00471	预知子	Yuzhizi	Akebiae Fructus	Akebia Fruit	理气药	Qi Regulating Drugs	0.0107264	0.040038	1	By_ingredient	0.4813
SMHB00472	芫花	Yuanhua	Genkwa Flos	flower bud of Lilac Daphne	逐水药	Drastic Purgatives	4.54E-08	4.38E-06	0.00401245	By_ingredient	1.2013
SMHB00473	芫荽	Yuansui	None	Coriandri Sativi Herba	辛温解表药	Pungent-Warm Exterior-Releasing Medicinal	1.477e-04	0.00191081	1	By_ingredient	0.738
SMHB00474	月季花	Yuejihua	Rosae Chinensis Flos	Chinese rose flower	活血祛瘀药	Blood Activating Stasis Removing Drugs	2.857e-04	0.00304013	1	By_ingredient	0.6896
SMHB00477	皂角刺	Zhaojiaoci	Gleditsiae Spina	spine of Chinese Honeylocust	化痰药	Phlegresolving Medicine	2.503e-04	0.00627418	1	By_ingredient	0.4519
SMHB00478	泽兰	Zelan	Lycopi Herba	Hirsute Shiny Bugleweed Herb	活血祛瘀药	Blood Activating Stasis Removing Drugs	3.453e-04	0.00344911	1	By_ingredient	0.6798
SMHB00479	泽漆	Zeqi	None	Euphorbiae Helioscopiae Herba	利水渗湿药	Diuretic Dampness Excreting Drugs	3.448e-04	0.00344491	1	By_ingredient	0.4795
SMHB00480	泽泻	Zexie	Alismatis Rhizoma	rhizome of Oriental Waterplantain	利水渗湿药	Diuretic Dampness Excreting Drugs	0.0193274	0.0603893	1	By_ingredient	0.3321
SMHB00482	知母	Zhimu	Anemarrhenae Rhizoma	rhizome of Common Amarrhe	清热泻火药	Fire Purging Drugs	0.128426	0.154193	1	By_ingredient	0.3422
SMHB00483	栀子	Zhizi	Gardeniae Fructus	fruit of Cape Jasmine	清热泻火药	Fire Purging Drugs	2.33E-05	5.198e-04	1	By_ingredient	1.1951
SMHB00484	枳椇子	Zhijuzi	None	Hoveniae Dulcis Semen	利水渗湿药	Diuretic Dampness Excreting Drugs	3.86E-05	7.509e-04	1	By_ingredient	0.4561
SMHB00485	枳壳	Zhiqiao	Aurantii Fructus	fruit of Seville orange	理气药	Qi Regulating Drugs	0.0291002	0.0498827	1	By_ingredient	0.1689
SMHB00486	枳实	Zhishi	Aurantii Fructus Immaturus	immature fruit of Seville orange	理气药	Qi Regulating Drugs	1.603e-04	0.00201534	1	By_ingredient	0.4515

Herb id	Chinese name	Pinyin name	Latin name	English name	Class in Chinese	Class in English	P_value	FDR(BH)	FDR(Bonferroni)	Relationship	Inferred evidence score
SMHB00487	肿节风	Zhongjiefeng	Sarcandrae Herba	all-grass of Glabrous sarcandra	清热解毒药	Antipyretic Detoxicate Drugs	0.00825251	0.0441675	1	By_ingredient	0.3191
SMHB00489	猪牙皂	Zhuyazao	Gleditsiae Fructus Abnormalis	Chinese Honeylocust Abnormal Fruit	化痰药	Phlegresolving Medicine	0.0417844	0.0832496	1	By_ingredient	0.1834
SMHB00492	苎麻根	Zhumagen	Radix Boehmeriae	Ramine root	凉血止血药	Blood-Cooling Hemostatic Medicinal	0.00266861	0.0275998	1	By_ingredient	0.2938
SMHB00493	紫草	Zicao	Arnebiae Radix	Arnebia Root Gromwell Root	清热凉血药	Heat Clearing Blood Cooling Drugs	0.003532	0.0160937	1	By_ingredient	0.5162
SMHB00496	紫苏叶	Zisuye	Perillae Folium	None	辛温解表药	Pungent-Warm Exterior-Releasing Medicinal	0.00230003	0.0127879	1	By_ingredient	0.7039
SMHB00497	紫苏子	Zisuzi	Perillae Fructus	Perilia Fruit	止咳平喘药	Antitussive Antiasthmetics	0.0207198	0.0401485	1	By_ingredient	0.2736
SMHB00498	紫菀	Ziwan	Asteris Radix Et Rhizoma	root of tatarian aster	止咳平喘药	Antitussive Antiasthmetics	1.03E-08	1.30E-06	9.103e-04	By_ingredient	0.9938
SMHB00499	葛根	Gegen	Puerariae Lobatae Radix	root of lobed kudzuvine	辛温解表药	Pungent-Warm Exterior-Releasing Medicinal	4.393e-04	0.00405531	1	By_ingredient	0.4457
SMHB00509	大豆黄卷	Dadouhuangjuan	Sojae Semen Germinatum	None	辛凉解表药	Pungent Cool Diaphoretics	0.00567342	0.0383771	1	By_ingredient	0.1667
SMHB00515	山楂	Shanzha	Crataegi Fructus	None	消食药	Digestants	0.0323485	0.0742611	1	By_ingredient	0.3185
SMHB00516	千里光	Qianliguang	Senecionis Scandentis Hebra	None	清热解毒药	Antipyretic Detoxicate Drugs	6.069e-04	0.0114172	1	By_ingredient	0.4546
SMHB00521	广金钱草	Guangjinqiancao	Desmodii Styracifolii Herba	None	利水渗湿药	Diuretic Dampness Excreting Drugs	7.036e-04	0.0126459	1	By_ingredient	0.516
SMHB00544	龙眼肉	Longyanrou	Longan Arillus	None	补虚药	Tonifying And Replenishing Medicinal	0.0169252	0.0578922	1	By_ingredient	0.1831
SMHB00555	母丁香	Mudingxiang	Caryophylli Fructus	None	温里药	Warming Interior Drugs	0.00131934	0.0185234	1	By_ingredient	0.4808
SMHB00572	合欢花	Hehuanhua	Albiziae Flos	None	安神药	Tranguilizing Medicinal	0.0113151	0.0500184	1	By_ingredient	0.1478
SMHB00575	红花龙胆	Honghualongdan	Gentianae Rhodanthae Herba	None	清热燥湿药	Heat-Clearing And Dampnessdrying Medicinal	0.0363143	0.0779393	1	By_ingredient	0.1606
SMHB00595	补骨脂	Buguzhi	Psoraleae Fructus	None	补虚药	Tonifying And Replenishing Medicinal	0.0208018	0.0623731	1	By_ingredient	0.3883
SMHB00601	苦木	Kumu	Picrasmae Ramulus Et Folium	None	清热药	Heat-Clearing Medicinal	0.0271455	0.0692226	1	By_ingredient	0.3686
SMHB00606	岩白菜	Yanbaicai	Bergeniae Rhizoma	None	收涩药	Astringent Medicinal	0.0280513	0.0703407	1	By_ingredient	0.2145
SMHB00612	炉甘石	Luganshi	Calamina	None	拔毒化腐生肌药	External Medicinal (Draw Out Toxin, Resolve Putridity)	0.0197184	0.0607932	1	By_ingredient	0.1674
SMHB00626	重楼	Chonglou	Paridis Rhizoma	None	清热药	Heat-Clearing Medicinal	0.00193771	0.023268	1	By_ingredient	0.3399
SMHB00629	独一味	Duyiwei	Lamiophlomis Herba	None	活血祛瘀药	Blood Activating Stasis Removing Drugs	1.35E-05	7.383e-04	1	By_ingredient	0.6334
SMHB00631	洪连	Honglion	Lagotidis Herba	None	清热解毒药	Antipyretic Detoxicate Drugs	4.75E-06	3.242e-04	1	By_ingredient	0.4407
SMHB00639	粉葛	Fenge	Puerariae Thomsonii Radix	None	辛凉解表药	Pungent Cool Diaphoretics	0.00849821	0.0445421	1	By_ingredient	0.1509
SMHB00645	黄山药	Huangshanyao	Dioscorea Panthaicae Rhizoma	None	理气药	Qi Regulating Drugs	0.0335677	0.0752792	1	By_ingredient	0.1643
SMHB00646	炙黄芪	Zhihuangqi	Astragali Radix Praeparata Cum Melle	None	补虚药	Tonifying And Replenishing Medicinal	0.0113151	0.0500184	1	By_ingredient	0.1707
SMHB00647	黄蜀葵花	Huangshukuihua	Abelmoschi Corolla	None	清热燥湿药	Heat-Clearing And Dampnessdrying Medicinal	0.0169252	0.0578922	1	By_ingredient	0.1451
SMHB00664	雄黄	Xionghuang	Realgar	None	攻毒杀虫止痒药	Toxin-Attacking And Worm-Expelling And Itch-Relieving Medicinal	0.0225039	0.0646491	1	By_ingredient	0.1637
SMHB00679	蒲公英	Pugongying	Taraxaci Herba	None	清热药	Heat-Clearing Medicinal	0.0265903	0.0685779	1	By_ingredient	0.3295
SMHB00682	蜂胶	Fengjiao	Propolis	None	补气药	Qi Reinforcing Drugs	0.00203918	0.0236699	1	By_ingredient	0.7187
SMHB00684	蜂蜜	Fengmi	Mel	None	补气药	Qi Reinforcing Drugs	0.0390532	0.0805384	1	By_ingredient	0.2075
SMHB00686	裸花紫珠	Luohuazizhu	Callicarpae Nudiflorae Folium	None	解毒杀虫燥湿止痒药	Medicinal For Detoxification、Parasiticide、Drying Dampness And Relieving Itching	0.0499322	0.0907582	1	By_ingredient	0.2165
SMHB00696	僵蚕	Jiangcan	Bombyx Batryticatus	None	平肝熄风药	Liver-Pacifying Wind-Extinguishing Medicinal	0.00193771	0.023268	1	By_ingredient	0.2904
