# Supplementary material for: TCGA-based identification of prognostic biomarkers and candidate traditional Chinese medicine compounds in papillary thyroid carcinoma: An observational study
Source: Medicine (Baltimore). 2026 Sep 4;105(36):e50562. doi: 10.1097/MD.0000000000050562 (PMC13549628; doi:10.1097/MD.0000000000050562)
Supplement: Supplementary file 3 [file medi-105-e50562-s003.pdf]

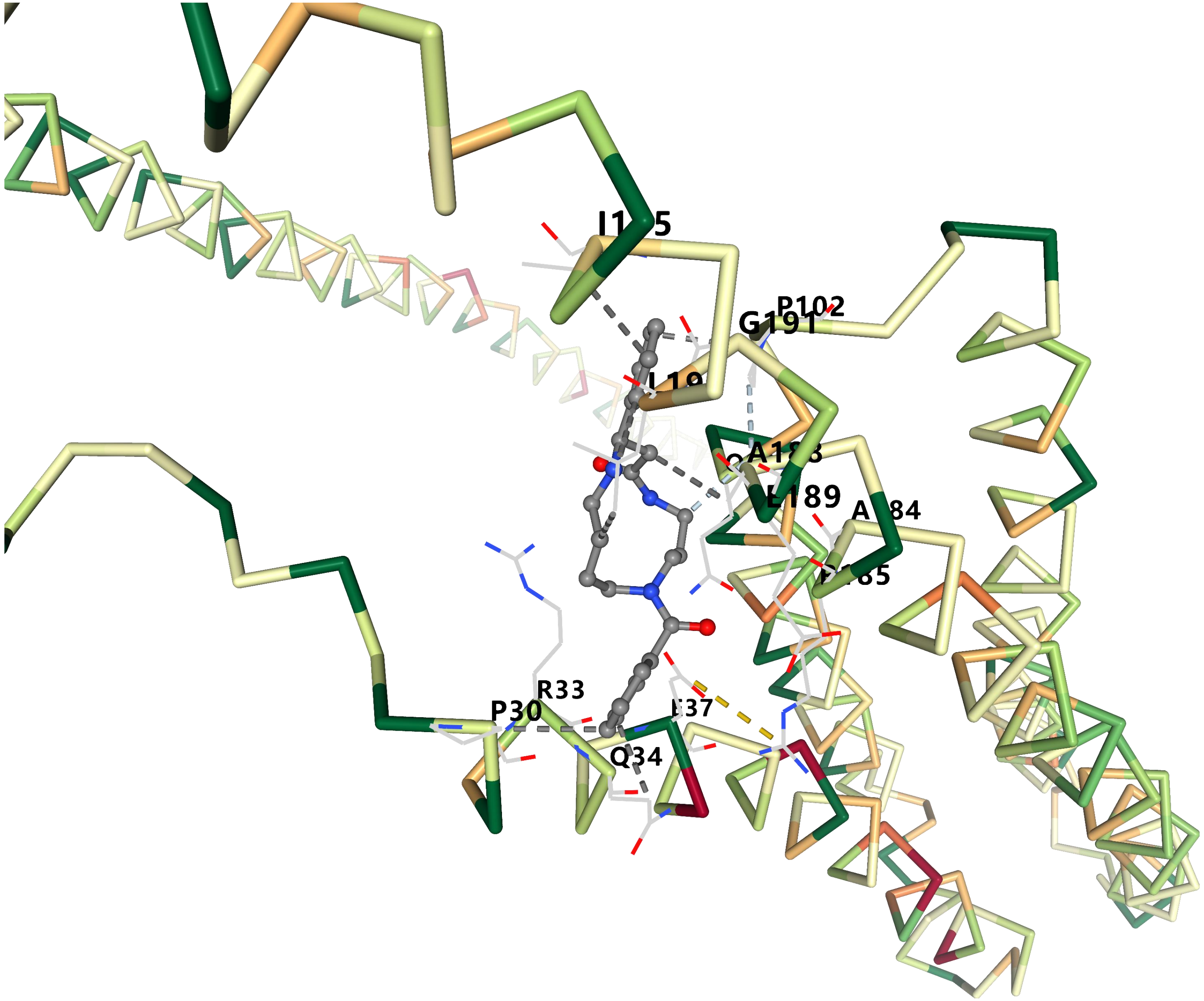

A. APOE–Ginseng   binding score -7.6

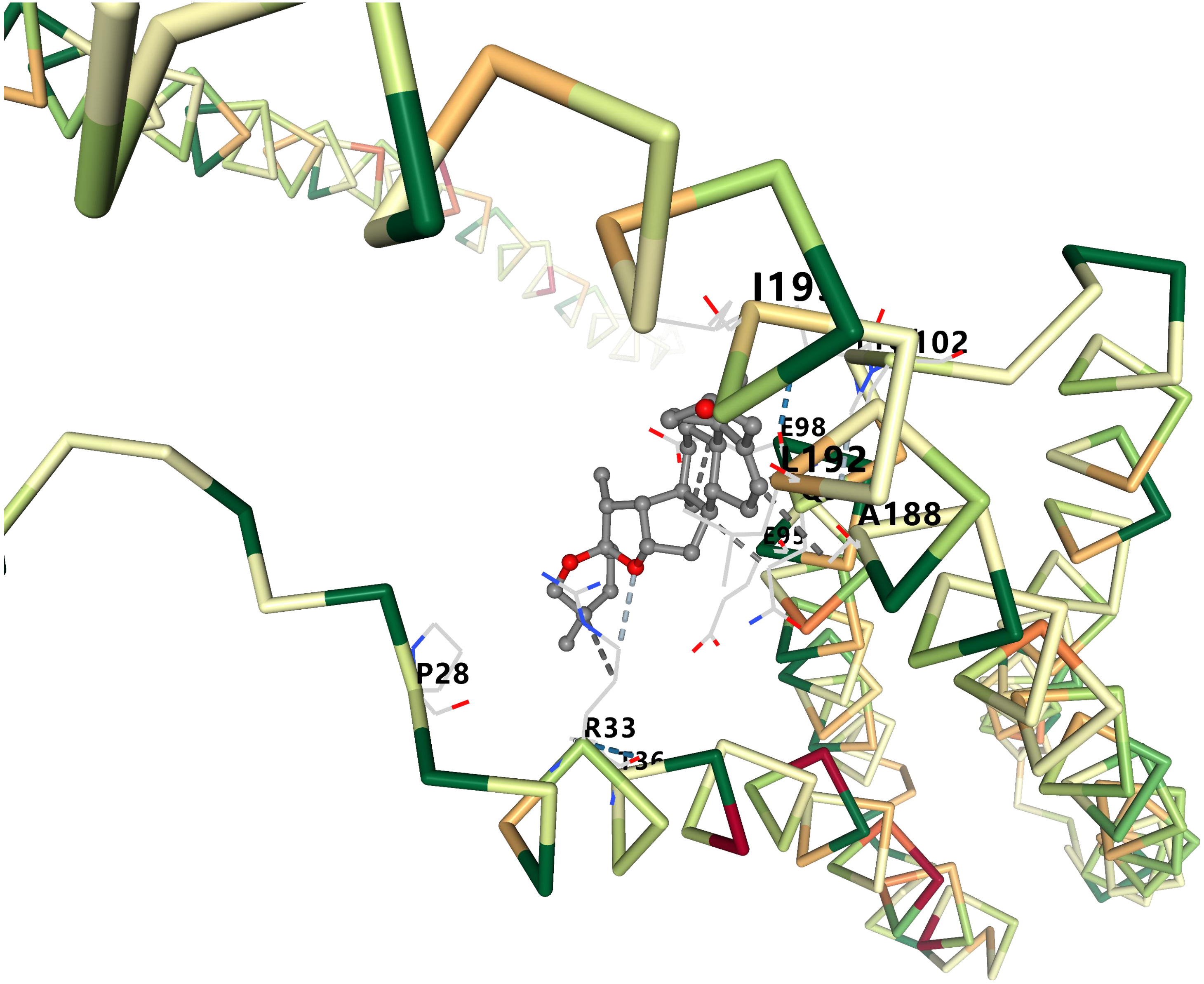

B. APOE–Smilax glabra binding score -8.0

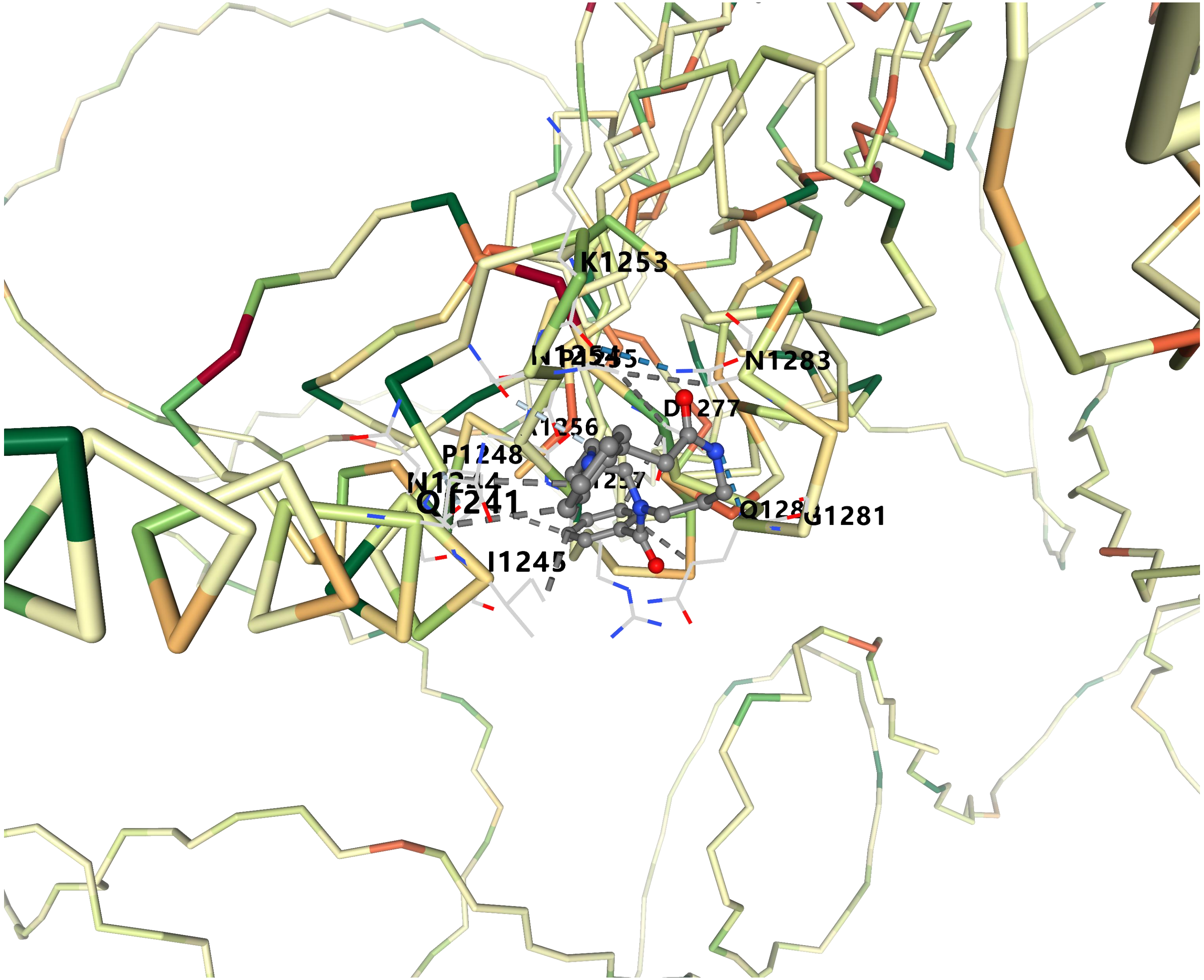

C. COL1A1–Ginseng   binding score -6.2

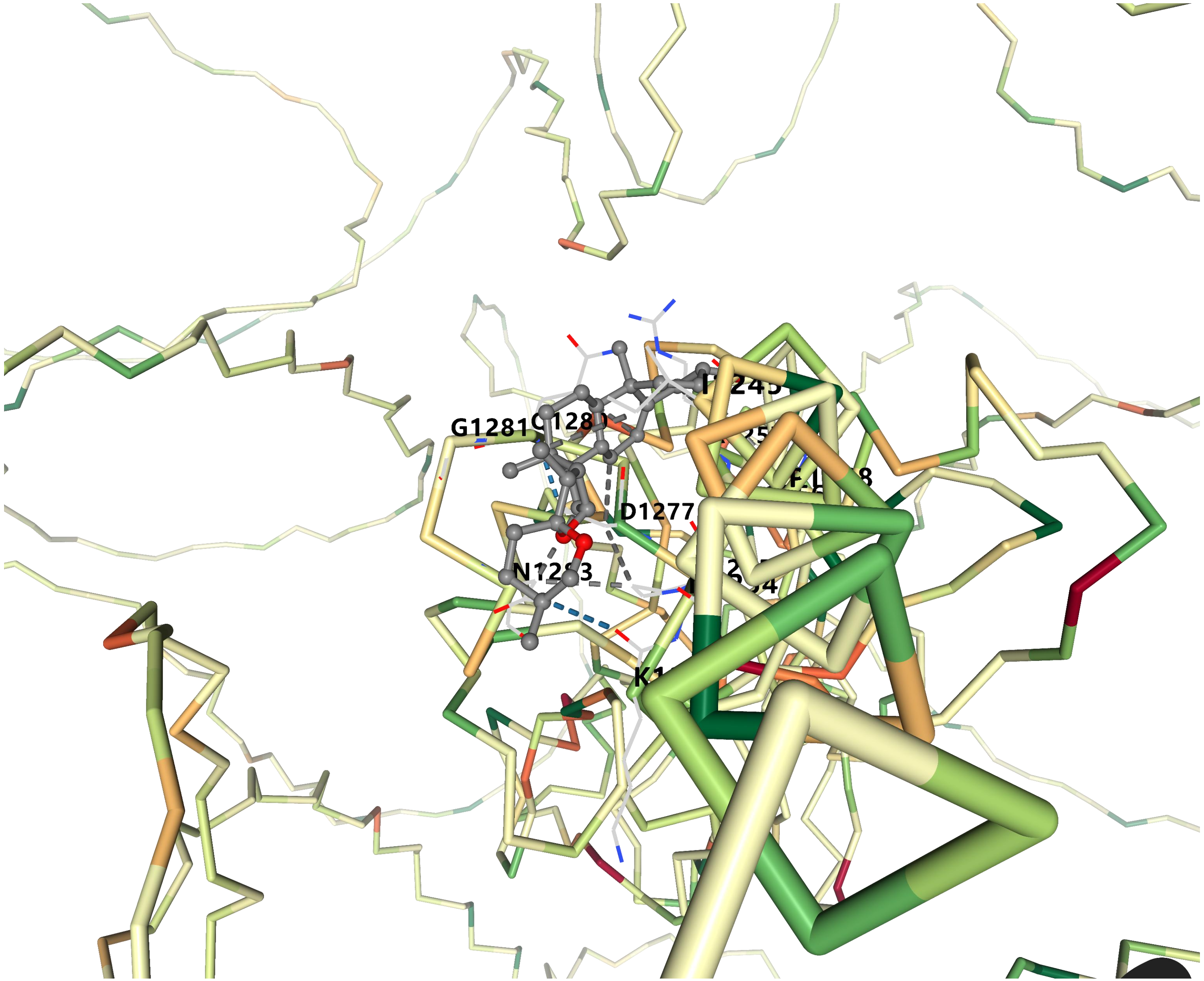

D. COL1A1–Smilax glabra   binding score -6.3

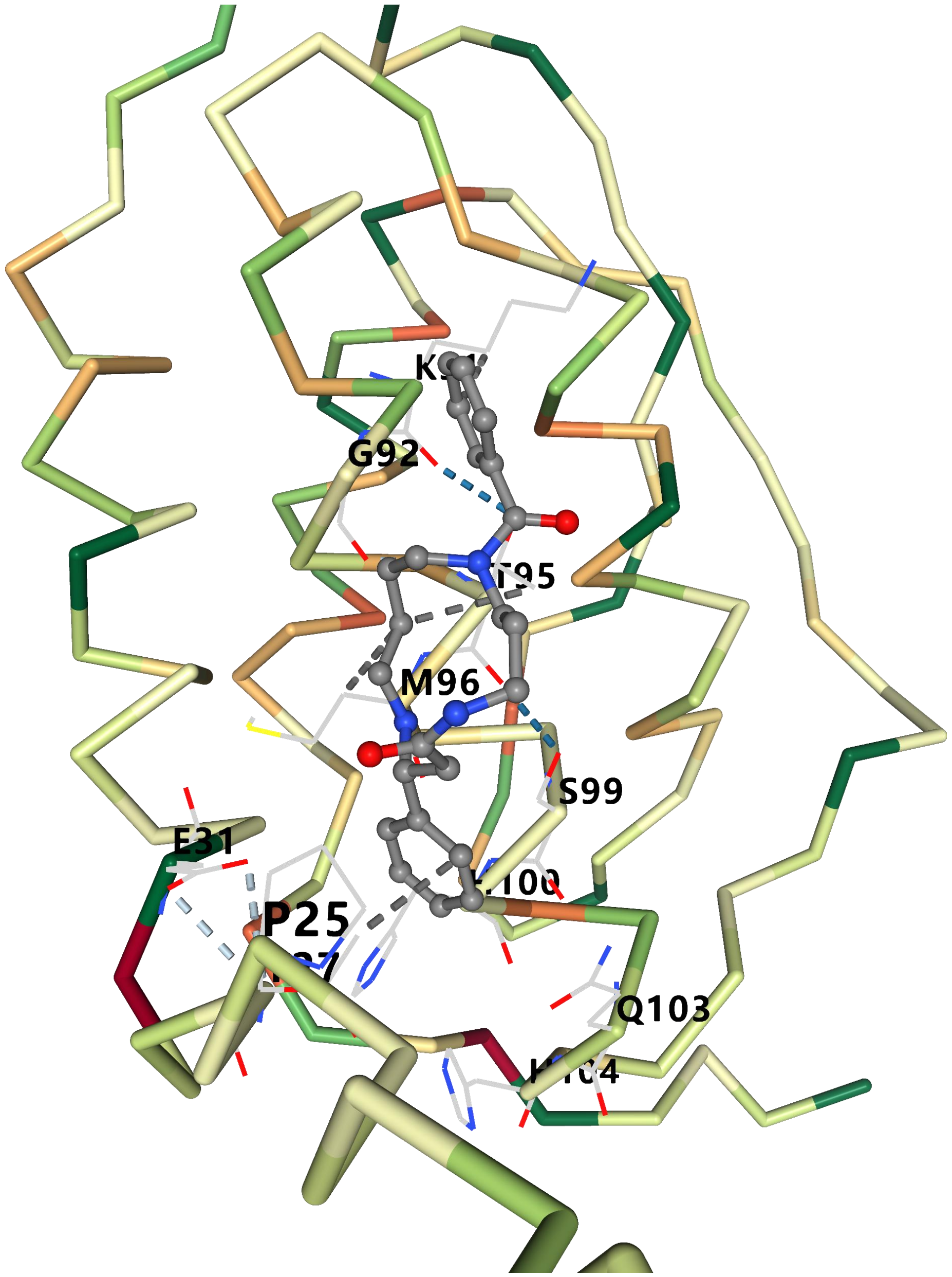

E. CSF2–Ginseng   binding score -6.7

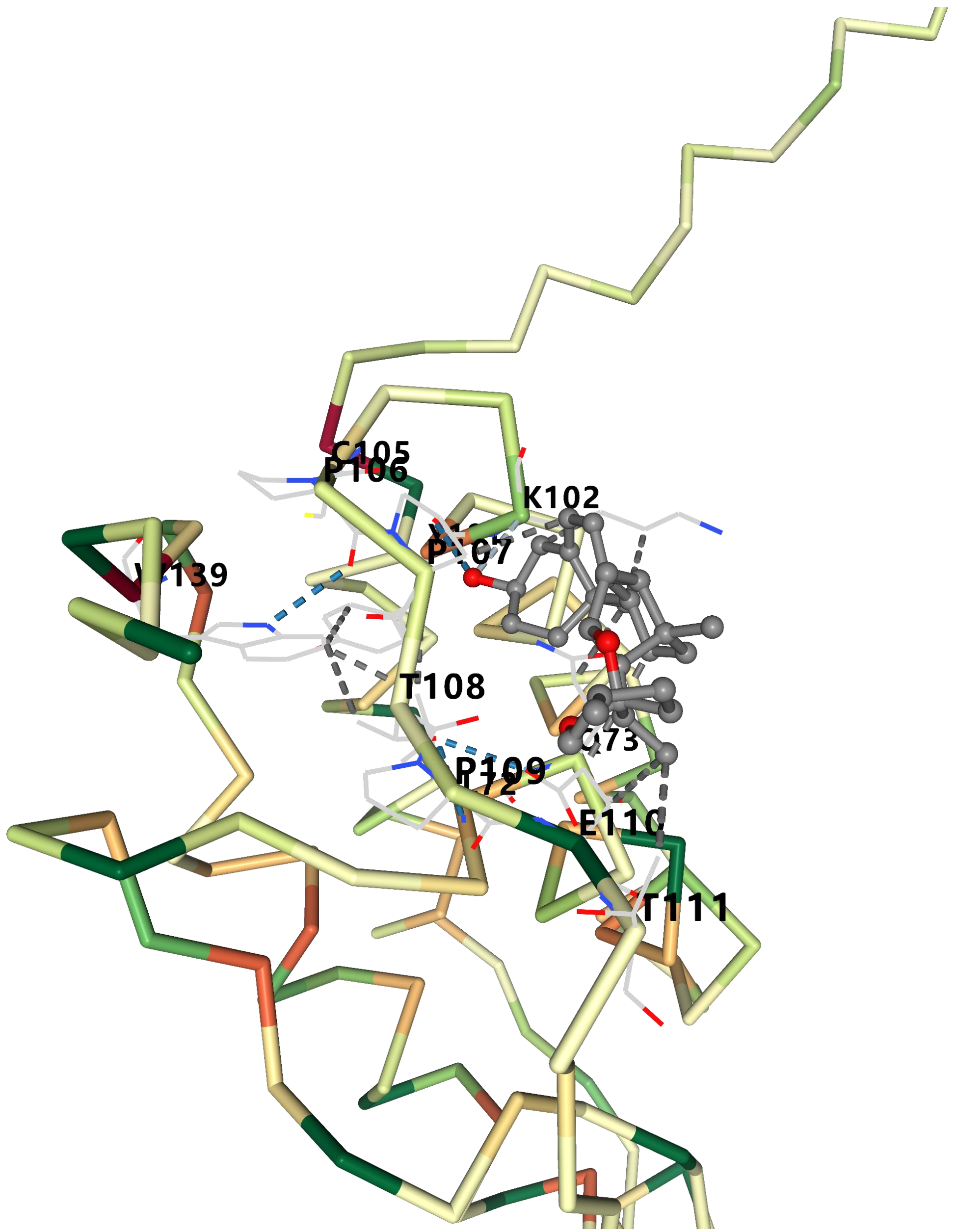

F. CSF2–Smilax glabra binding score -8.3
